# Conference Proceedings – 6th International Conference on Molecular Diagnostics and Biomarker Discovery (MDBD 2022): Building Resilience in Biomedical Research: Penang, Malaysia. 11-13 October 2022

**DOI:** 10.1186/s12919-022-00237-8

**Published:** 2022-12-21

## O-1 Tissue-based proteomics: insight into molecular mechanisms in cervical carcinogenesis

### Gaayathri Kumarasamy^1^, Mohd Nazri Ismail^1, 2^, Sharifah Emilia Tuan Sharif^3^, Christopher Desire^4^, Parul Mittal^4^, Peter Hoffmann^4^ and Gurjeet Kaur^1^

#### ^1^Institute for Research in Molecular Medicine (INFORMM), Universiti Sains Malaysia, 11800 Minden, Pulau Pinang, Malaysia; ^2^Analytical Biochemistry Research Centre (ABrC), Universiti Sains Malaysia, 11900 Bayan Lepas, Pulau Pinang, Malaysia; ^3^Department of Pathology, School of Medical Sciences, Universiti Sains Malaysia, 16150 Kubang Kerian, Kelantan, Malaysia; ^4^Clinical Health Sciences, University of South Australia, City West Campus, Adelaide, South Australia, 5000, Australia

##### **Correspondence:** Gurjeet Kaur (gurjeet@usm.my)

From The International Conference on Molecular Diagnostics & Biomarker Discovery 2022 (MDBD 2022)

Penang, Malaysia. 11 – 13 October 2022

**Background**

Tissue-based proteomics is an evolving tool used in cancer research to characterize the pathophysiology of disease. However, the proteome alterations involved in cervical carcinogenesis are not extensively studied. This study aims to elucidate the differentially expressed proteins and offer insights into the cellular processes and pathways involved in the development of cervical cancer.

**Methodology**

The pathological regions of interest in the cervical squamous epithelium were micro-dissected from formalin-fixed paraffin-embedded (FFPE) tissue sections of six normal cervix cases, five HPV-associated squamous intraepithelial lesion (SIL), and six squamous cell carcinomas (SCC). The samples were trypsin digestion and subjected to high throughput liquid chromatography-electrospray ionization-tandem mass spectrometry (LC/ESI-MS/MS) and trapped ion mobility time-of-flight-mass spectrometry (timsTOF-MS), followed by quantification with MaxQuant and Perseus software. Bioinformatics analyses were carried out using DAVID, ConsensusPathDB, and STRING.

**Results and Discussion**

We identified a total of 3597 proteins with 589, 550, and 1570 proteins unique to the normal cervix, SIL, and SCC groups, respectively, while 332 proteins were similar across all three groups. The predominant protein found was histone. Interestingly, the quantification results showed an upward trend for the up-regulated proteins and a downward trend for the down-regulated proteins in the progression from normal to SIL and SCC. The main molecular function was the binding process, and the top biological processes were chromatin silencing for SIL compared to the normal cervix and nucleosome assembly for the SCC compared to SIL group. The key pathways involved were viral carcinogenesis and necroptosis, reflecting their role in cell proliferation, migration, and metastasis.

**Conclusion**

The identification of proteins and their associated pathways provides a deeper understanding of the underlying molecular mechanisms involved in HPV-associated cervical cancer.

**Acknowledgement:** The work was funded by the Fundamental Research Grant Scheme (FRGS/1/2019/SKK13/USM/01/1) from the Ministry of Education, Malaysia.

**References**

[1] F. Bray, J. Ferlay, I. Soerjomataram, R.L. Siegel, L.A. Torre, A. Jemal, Global cancer statistics 2018: GLOBOCAN estimates of incidence and mortality worldwide for 36 cancers in 185 countries, CA. Cancer J. Clin. 68 (2018) 394–424.

[2] W.S. Jr, M.A. Bacon, A. Bajaj, L.T. Chuang, Cervical cancer: a global health crisis, (2017) 1–9.

[3] M. Vu, J. Yu, O.A. Awolude, L. Chuang, Cervical cancer worldwide, Curr. Probl. Cancer. 42 (2018) 457–465.

## O-2 The expression of apoB and 4HNE in overweight-and obese-related colorectal carcinoma tissues

### Ng Phei Ying^1^, Siti Norasikin Mohd Nafi^1, 4^, Nur Asyilla Che Jalil^1, 4^, Kueh Yee Cheng^3^, Lee Yeong Yeh^2, 4^ and Anani Aila Mat Zin^1, 4^

#### ^1^Department of Pathology, School of Medical Sciences, Health Campus, Universiti Sains Malaysia, 16150 Kubang Kerian, Kelantan, Malaysia; ^2^Department of Medicine, School of Medical Sciences, Health Campus, Universiti Sains Malaysia, 16150 Kubang Kerian, Kelantan, Malaysia; ^3^Biostatistics and Research Methodology Unit, School of Medical Sciences, Health Campus, Universiti Sains Malaysia, 16150 Kubang Kerian, Kelantan, Malaysia; ^4^Hospital Universiti Sains Malaysia, Health Campus, Universiti Sains Malaysia, 16150 Kubang Kerian, Kelantan, Malaysia

##### **Correspondence:** Siti Norasikin Mohd Nafi (snmn@usm.my)

From The International Conference on Molecular Diagnostics & Biomarker Discovery 2022 (MDBD 2022)

Penang, Malaysia. 11 – 13 October 2022

**Background**

Obesity has been found to be related to an increase in the incidence and progression of colorectal carcinoma (CRC) [1]. However, the relationship between obesity and CRC is still unclear. Low-density lipoprotein (LDL) is known for causing abnormal lipid metabolism and is usually measured in the blood [2]. Two LDL-related biomarkers, apoB and 4HNE, were chosen to investigate their expression in overweight-and obese-related CRC tissues, and their association with the clinicopathological data was also determined.

**Methodology**

Human ethical approval with a series code of USM/JEPeM 19060354 was obtained. This retrospective study involved overweight-and obese-related CRCs diagnosed in HUSM from January 2015 to December 2021. The classification of BMI followed the Asia Pacific BMI classification [3]. Clinicopathological data was retrieved from Laboratory Information System (LIS) and medical records at Pathology laboratory, HUSM. The CRC archival tissue blocks were then collected for Immunohistochemistry (IHC) staining. The data was then analysed using SPSS Version 26.

**Results and Discussion**

A total of 69 overweight-and obese-related CRCs were retrieved within seven years. The patients had median age of 61 years old with interquartile range of 21, common in age of more than 50 years old (73.9%) and in males (52.2%). Most of the patients were found with CRCs localised in sigmoid colon (30.4%) and rectosigmoid colon (24.6%), larger than 2cm of tumour (97.1%), presence of bowel wall invasion (94.2%), and absence of lymph nodes involvement (58.0%). The tumours were frequently classified as adenocarcinoma (91.3%) with moderate differentiation grade (82.6%) and under Modified Duke B class (55.1%). The IHC-stained CRC tissue slides showed high apoB expression (91.3%), whereas low 4HNE expression (82.6%). Significant associations were observed between apoB expression with age (*p* value=0.036), tumour site (*p* value=0.048), bowel wall invasion (*p* value=0.035), Duke classification (*p* value=0.002), while 4HNE expression with tumour size (*p* value=0.029). Majority of CRC tumours of more than 2cm showed low 4HNE expression. This may be explained by the protective effect of low 4HNE level that prevents the damage of the cancer cells [4].

**Conclusion**

In conclusion, the contrast tissue expression of apoB and 4HNE, and the significant associations obtained has shed light on the role of these LDL-related biomarkers in overweight-and obese-related CRC tissues. Exploration on the role of these biomarkers is recommended for *in vitro* study.

**Acknowledgement:** The work was funded by Long Term Research Grant Scheme (LRGS) LR001-2019 (203/PPSP/6720019) from the Malaysian Research University Network (MRUN).

**References**

[1] Ye PF, et al. Linking obesity with colorectal cancer: epidemiology and mechanistic insights. Cancers. 2020; 12:1408-27.

[2] Fang Z, et al. Serum lipid profiles and risk of colorectal cancer: a prospective cohort study in the UK Biobank. British J of Cancer. 2020; 124:663–70.

[3] WHO/IOTF/IASO. (2000) World Health Organization, International Obesity Task Force, International Association for the Study of Obesity., Hong Kong.

[4] Zhong HQ, et al. Role of lipid peroxidation derived 4-hydroxynonenal (4-HNE) in cancer: Focusing on mitochondria. Redox Biology. 2015; 4:193-9.

## O-3 Quantitative proteomics analysis of insecticide resistant *Ae*. *aegypti*

### Abubakar Shettima^1, 2^, Intan H Ishak^3, 4^, Benjamin Lau^5^, Hadura Abu Hasan^3,4^ and Noorizan Miswan^6^ and Nurulhasanah Othman^1^

#### ^1^Institute for Research in Molecular Medicine (INFORMM), Universiti Sains Malaysia, Penang, Malaysia; ^2^Department of Microbiology, University of Maiduguri, Maiduguri, Nigeria; ^3^School of Biological Sciences (SBS), Universiti Sains Malaysia, Penang, Malaysia; ^4^Vector Control Research Unit (VCRU), Universiti Sains Malaysia, Penang, Malaysia; ^5^ Malaysian Palm Oil Board (MPOB), Selangor, Malaysia; ^6^Center for Chemical Biology (CCB), Universiti Sains Malaysia, Penang, Malaysia

##### **Correspondence:** Nurulhasanah Othman (nurulhasanah@usm.my)

From The International Conference on Molecular Diagnostics & Biomarker Discovery 2022 (MDBD 2022)

Penang, Malaysia. 11 – 13 October 2022

**Background**

Synthetic insecticides are the main vector control method globally. However, the widespread use of insecticide is causing resistance in mosquitoes. Understanding the proteomics basis of insecticide resistance may provide novel opportunities to control mosquito vectors. Hence, this study aimed to elucidate proteins associated with permethrin resistant *Ae. aegypti* using quantitative proteomics.

**Methodology**

The study evaluated the resistance pattern of *Ae. aegypti* from dengue hotspot and non-hotspot areas of Penang Island. Permethrin 0.75% insecticide-impregnated papers were used to determine the resistance status in adult female *Ae. aegypti*. The mosquito proteins were analysed using LC–ESI–MS/MS for protein identification and quantification via label-free quantitative (LFQ) analysis. In this study, differential protein expression (DEP) analysis was carried out using Perseus 1.6.14.0 statistical software to perform ANOVA and student’s t-test. Protein-protein interaction (PPI) and functional ontology enrichment analyses were performed using STRING software.

**Results and Discussion**

The bioassay results showed 28% and 53% mortalities in mosquitoes exposed to permethrin from the hotspot and non-hotspot areas. Permethrin has been used extensively in Malaysia’s dengue vector control program. The mortality percentage has shown a high permethrin resistance rate in the field strain *Ae. aegypti* mosquito, including the strains from the dengue hotspot and non-hotspot areas. These results suggested high resistance status in the dengue hotspot and non-hotspot areas. The high resistant patterns identified in the field strain *Ae. aegypti* could be because of the widespread and indiscriminate permethrin use in the investigated areas, especially if a site was labeled as a dengue hotspot. The resistance in the non-hotspot area could be because the area was once a dengue hotspot. These areas received extra attention from vector control management and private contractors lead to insecticide selection pressure and produce offspring carrying insecticide-resistant genes. The LFQ analyses revealed 501 (q-value <0.05) DEPs. The t-test showed 114 upregulated and 74 downregulated proteins in resistant versus laboratory strain exposed to permethrin. The significant functional ontology enrichment of the DEPs indicated drug-metabolic process, small molecule metabolic process, hydrolase activity acting on ester bonds, and catalytic activity. The PPI of DEPs showed a p-value at <1.0 x 10^-16^ in permethrin-resistant *Ae. aegypti*. Significantly enriched pathways in DEPs revealed metabolic pathways, oxidative phosphorylation, carbon metabolism, biosynthesis of amino acids, glycolysis, and citrate cycle.

**Conclusion**

This study has revealed several DEPs and highlighted upregulated and downregulated proteins associated with insecticide resistance in *Ae. aegypti*.

## O-4 Clarithromycin resistance in *Helicobacter Pylori* is associated with genetic polymorphism of virulence factors

### Anis Rageh Al-Maleki and Naim Rosli

#### Department of Medical Microbiology, Faculty of Medicine, Universiti Malaya, Kuala Lumpur, Malaysia

##### **Correspondence:** Anis Rageh Al-Maleki (anisrageh@um.edu.my)

From The International Conference on Molecular Diagnostics & Biomarker Discovery 2022 (MDBD 2022)

Penang, Malaysia. 11 – 13 October 2022

**Background**

For a long time, the macrolide antibiotic clarithromycin was used to treat *Helicobacter pylori* (*H. pylori*) infections. The efficiency of traditional triple therapy for the eradication of *H. pylori* has recently been dismally reduced in many nations [1]. Clarithromycin resistance is spreading over the world and is the leading cause of *H. pylori* treatment failure [2]. We employed whole-genome sequencing (WGS) technology to identify genomic alterations related with the development of antibiotic resistance in *H. pylori* with induced resistance to Clarithromycin.

**Methodology**

The clarithromycin-sensitive *H. pylori* strains were induced to become resistant against clarithromycin. To induce resistance, the strains were exposed to gradually increased concentration of clarithromycin in vitro for 3-5 days on a chocolate agar plate (CAP). The identity between resistant strains and their corresponding parental sensitive strains before induction were verified by random amplification of polymorphic DNA polymerase chain reaction (RAPD-PCR). WGS for bacterial DNA was performed, using Illumina platform.

**Results and Discussion**

There were 113 distinct single nucleotide variants (SNVs) and 286 unique insertions and deletions (InDels) changes discovered among the induced-resistant *H. pylori* strains. Interestingly, among the induced-resistant *H. pylori* strains, mutations in *cag1*, *cag4*, *fliR*, *obgE*, *tlpA*, and *vacA* were detected, which are known virulence genes that may help in bacterial survival, indicating that those mutations may be associated with the emergence of resistant *H. pylori* [3]. Moreover, we also found that it is not possible to infer a Clarithromycin resistance phenotype based on the existence of distinct point mutations in A2143G and/or A2142G mutations in 23S rRNA and may not be sufficient for predicting Clarithromycin resistance in *H. pylori*.

**Conclusion**

As a result, we hypothesise that in these strains, alternative mechanisms unrelated to the 23S rRNA gene sequence, such as the existence of an efflux pump, may play a role in Clarithromycin resistance.

**Acknowledgement:** The work is supported financially by the Ministry of Higher Education Malaysia (MOHE) via Fundamental Research Grant Scheme (FRGS/1/2020/SKK0/UM/02/20), Project No. (FP105-2020).

**References**

[1] Giorgio F, Principi M, De Francesco V, Zullo A, Losurdo G, Di Leo A, Ierardi E. Primary clarithromycin resistance to Helicobacter pylori: Is this the main reason for triple therapy failure?. World journal of gastrointestinal pathophysiology. 2013 Aug 8;4(3):43.

[2] Graham DY. *Helicobacter pylori* update: gastric cancer, reliable therapy, and possible benefits. *Gastroenterology*. 2015;148(4):719-31. e3.

[3] Verstraeten N, Knapen WJ, Kint CI, Liebens V, Van den Bergh B, Dewachter L, et al. Obg and Membrane Depolarization Are Part of a Microbial Bet-Hedging Strategy that Leads to Antibiotic Tolerance. *Mol Cell*. 2015;59(1):9-21.

## O-5 Metabolome analysis of induced-resistance *Helicobacter pylori* against Clarithromycin

### Naim Rosli and Anis Rageh Al-Maleki

#### Department of Medical Microbiology, Faculty of Medicine, Universiti Malaya, Kuala Lumpur, Malaysia

##### **Correspondence:** Anis Rageh Al-Maleki (anisrageh@um.edu.my)

From The International Conference on Molecular Diagnostics & Biomarker Discovery 2022 (MDBD 2022)

Penang, Malaysia. 11 – 13 October 2022

**Background**

*Helicobacter pylori* (*H. pylori*) is a gram-negative bacterium that thrives in stomach mucus and epithelial mucosa, causing gastric ulcers which may develop into gastric cancer. One of the most prevalent causes of treatment failure is the emergence of antibiotic-resistant of *H. pylori* infection [1]. The goal of this work is to use a metabolomic method to discover the metabolites associated with Clarithromycin-resistance in *H. pylori*.

**Methodology**

Clarithromycin-sensitive *H. pylori* strains were induced to become resistant against Clarithromycin. To induce resistance, the strains were treated to 0.1x, 0.25x, 0.5x, 1X, 2x, 4x, 8x, 16x, and 32x MICs of Clarithromycin in vitro for 3-5 days. Bacterial metabolites were isolated using the Bligh and Dyer technique and analysed using liquid chromatography-mass spectrometry (LCMS) [2]. The MassHunter Qualitative Analysis and Mass Profiler Professional tools were used to process and analyse the data. Sensitive, pre-resistant, and induced-resistant are three separate categories based on global metabolomic patterns.

**Results and Discussion**

Using one-way ANOVA, a total of 982 molecular features were identified to be significantly different (p-value < 0.005) between sensitive, pre-resistant, and resistant *H. pylori* strains. Additionally, 432 molecular features matched the metabolites in the Agilent METLIN Accurate Mass-Personal Metabolite Database and Library (AM-PCDL) database based on accurate mass, isotope ratios, abundances and spacing. In contrast to sensitive strains, induced-resistant strains generated more metabolites (585 features). Further investigation was carried out in order to find metabolites that varied substantially (p-value <0.05) between sensitive, pre-resistant, and induced-resistant *H. pylori* strains. These metabolites include lipids and metabolites involved in the metabolism of fructose and mannose. Our data imply that D-Mannitol, L-Leucine, and Pyridoxine, which correlate with bacterial survival, may constitute a potential antibiotic mechanism [3].

**Conclusion**

Understanding the underlying metabolic variations between Clarithromycin-sensitive *H. pylori* strains and Clarithromycin-resistance *H. pylori* strains may be a promising technique for developing novel antibiotic candidates.

**Acknowledgement:** The work is supported financially by the Ministry of Higher Education Malaysia (MOHE) via Fundamental Research Grant Scheme (FRGS/1/2020/SKK0/UM/02/20), Project No. (FP105-2020).

**References**

[1] Kocsmár É, Buzás GM, Szirtes I, Kocsmár I, Kramer Z, Szijártó A, et al. Primary and secondary clarithromycin resistance in *Helicobacter pylori* and mathematical modeling of the role of macrolides. *Nat. Commun.* 2021;12(1):2255.

[2] Bligh EG, Dyer WJ. A rapid method of total lipid extraction and purification. *Can. J. Biochem. Physiol.* 1959;37(8):911-7.

[3] Barra ALC, Dantas LOC, Morão LG, Gutierrez RF, Polikarpov I, Wrenger C, et al. Essential Metabolic Routes as a Way to ESKAPE From Antibiotic Resistance. *Front. Public Health*. 2020;8:26.

## O-6 The antimicrobial effect of vinegar on *Escherichia coli* O157:H7 isolated from lettuce

### Yu Xiang Soo^1^ and Seow Hoon Saw^1,2^

#### ^1^Department of Allied Health Sciences, Faculty of Science, Universiti Tunku Abdul Rahman, 31900 Kampar, Perak, Malaysia; ^2^Centre for Research on Communicable Diseases, Faculty of Medicine and Health Sciences, Universiti Tunku Abdul Rahman, Jalan Sungai Long, Bandar Sungai Long, 43000 Kajang, Selangor, Malaysia

##### **Correspondence:** Seow Hoon Saw (sawsh@utar.edu.my)

From The International Conference on Molecular Diagnostics & Biomarker Discovery 2022 (MDBD 2022)

Penang, Malaysia. 11 – 13 October 2022

**Background**

Lettuce *(Lactuca sativa)* is a ready-to-eat (RTE) vegetable, which is popular among consumers due to its convenience and high nutritional value. Unfortunately, lettuce is frequently associated with *Escherichia coli* O157:H7 outbreaks that cause serious illnesses such as haemolytic uremic syndrome and haemorrhagic colitis [1]. Thus, a proper cleaning of the products is crucial to eliminate the risk of foodborne pathogens contamination. Acetic acid, such as vinegar, has shown its antimicrobial effect on inhibiting the growth of *E. coli* O157:H7 [2]. In addition, it is commonly used in the household settings for cooking and washing. Hence, the objectives for this study were identifying and verifying the presence of *E. coli* O157:H7 on lettuces purchased from retail market in Kampar Perak, using multiplex polymerase chain reaction (mPCR); followed by evaluating the antimicrobial activity of vinegar based on the analysis of log reduction in growth of *E. coli* O157:H7.

**Methodology**

A total of 20 samples of lettuce was purchased from a retail market in Kampar Perak. Homogenization was performed on 10 g of shredded lettuce, followed by incubation at 37°C for 24 h in Tryptic Soy Broth (TSB) to enrich the bacteria. Serial dilution was performed prior to plating on selective agar for *E. coli* O157:H7*.* Verification of colonies was done using multiplex polymerase chain reaction (PCR) with primer pairs targeting genes of *rfb*_*O157*_ (256 bp) and *flic*_*H7,*_ (625 bp). Both encodes for somatic and flagellar antigen, respectively. On the other hand, 1.0 McFarland ATCC 700728 *E. coli* O157:H7 was prepared and inoculated on lettuce at 5°C for 1 h. 5% of white vinegar was immersed into the ‘contaminated’ lettuce at 4 time points:10, 20, 30 and 40 mins. Antibacterial effect was evaluated by enumerating the reduction of bacteria colonies in log CFU/g. The significance of log reduction of inoculated bacteria was analysed quantitatively using One-Way ANOVA test (Graph Pad Prism 9.0) with 95 % confidence level.

**Results & Discussion**

None of the samples collected were found contaminated with *E. coli* O157:H7. This might be due to the safety measures taken by the Food Safety and Quality Division (FoSIM) in maintaining the quality of products in the retail market. However, in this study, 5 samples of lettuce (25%) showed presence of *flic*_*H7*_ gene but not *rfb*_*O157*_ virulence gene*.* This might postulate the presence of other *E. coli* serotypes such as O55:H7 which shares similarity in *flic* H7 gene. On the other hand, significant reduction of *E. coli* O157:H7 was observed after 40 min treatment with 5% vinegar. Organic acids exert their antimicrobial effect by altering the pH level of bacteria’s cytoplasm which then leads to cell lysis due to the disruption of enzymes’ and proteins’ structures [3]. Hence, acetic acid is the best alternative to chlorinated water because of its antimicrobial and consumer-friendly properties.

**Conclusion**

Lettuces purchased from Kampar are safe to be consumed. Vinegar is effective to reduce the growth of *E. coli O157:H7* on RTE vegetables after a period. Nevertheless, various parameters including pH of vinegar shall be studied to determine its optimum effectiveness towards eliminating bacteria.

**Acknowledgement:** Fundamental Research Grant Scheme: FRGS/1/2022/SKK06/UTAR/02/1 (8160/0013)

**References**

[1] Coulombe, G., et al. Outbreak of *Escherichia coli* O157:H7 infections linked to romaine lettuce in Canada from 2008 to 2018: An analysis of food safety context. Journal of Food Protection. 2020, 83(8): 1444-1462.

[2] Poimenidou, S.V., et al. Effect of single or combined chemical and natural antimicrobial interventions on *Escherichia coli* O157:H7, total microbiota and colour of packaged spinach and lettuce. International Journal of Food Microbiology. 2016, 220: 6–18.

[3] Warnecke, T. and Gill, R.T., 2005. Organic acid toxicity, tolerance, and production in Escherichia coli biorefining applications. Microbial Cell Factories, 2005, 4(1): 25-27.

## O-7 Raman-based spectroscopic techniques for *Leptospira* DNA detection

### Anis Athirah Abdul Razak^1^, Fatin Hamimi Mustafa^2^, Hui Yee Chee^3,4^, Mohd Adzir Mahdi^4^ and Fariza Hanim Suhailin^5^

#### ^1^Faculty of Science and Marine Environment, Universiti Malaysia Terengganu (UMT), Kuala Terengganu, Malaysia; ^2^Institute for Research in Molecular Medicine, Universiti Sains Malaysia Health Campus, Kubang Kerian, Malaysia; ^3^Department of Medical Microbiology, Faculty of Medicine and Health Sciences, Universiti Putra Malaysia (UPM), Serdang, Malaysia; ^4^Wireless and Photonic Networks Research Centre, Faculty of Engineering, UPM, Serdang, Malaysia; ^5^Physics Department, Faculty of Science, Universiti Teknologi Malaysia (UTM), Johor Bahru, Malaysia

##### **Correspondence:** Fariza Hanim Suhailin (farizahanim@utm.my)

From The International Conference on Molecular Diagnostics & Biomarker Discovery 2022 (MDBD 2022)

Penang, Malaysia. 11 – 13 October 2022

**Background**

Raman spectroscopy (RS) and surface enhanced Raman spectroscopy (SERS) are both promising techniques for biomolecule detection. In this article, we present our preliminary findings in the utilization of these techniques for *Leptospira* deoxyribonucleic acid (DNA) detection. A functional layer formed via a chemical organized monolayer (SAM) was used to immobilize DNA probe (pDNA) onto the metallic Raman layer/substrate. Successful hybridization between pDNA to the complementary DNA (cDNA) sequence was verified via vibrational features in Raman spectrum. The finding shows the potential use of Raman-based techniques as alternative diagnostic tools for leptospirosis.

**Methodology**

Two genes of *Leptospira*, i.e., *secY* and *lipL32*, were used. The following are the oligonucleotide sequences:

- *SecY* cDNA: 5’-TTT GAA GGG CAG GAA CAA G-3’
- *LipL32* cDNA: 5’-TTG TTT CCA TCG ACT AAA CCG TC-3’
- *SecY* pDNA: 5’-/5AmMC6/CTT GTT CCT GCC CTT CAAA-3’
- *LipL32* pDNA: 5’-/5AmMC6/GAC GGT TTA GTC GAT GGA AAC AA-3’

*SecY* gene is a housekeeping gene whereas *lipL32* gene is a marker for pathogenic gene. The pDNAs were terminated with amine for rapid attachment to metallic Raman layer/substrate. A 50 nm gold (Au) thin-film and 80 nm in size bi-metallic gold- silver nanoparticles (Au-Ag NPs) were the metallic Raman layer/substrate for RS and SERS measurements, respectively. The Au thin-film was deposited onto a fused silicate layer by plasma sputtering, while the colloidal Au-Ag NPs were embedded onto the polymer-based photonic crystal substrate via polyethylenimine (PEI) adhesive. The metallic surfaces were SAM-modified; (1) for Au thin-film, 3-mercaptopropionic acid (MPA) and 1-ethyl-3-(3-dimethyl-aminopropyl) carbodiimide hydrochloride/N-hydroxyl succinimide (EDC/NHS) were used to achieve thiolate- and aminate-modified surfaces, (2) for Au- Ag NPs, the 4-aminothiophenol (4-ATP) was used to obtain the amine tail on the surface. After the functionalization process, the metallic Raman layer/substrate was incubated with 1 μM pDNA and cDNA in sequence. Raman spectrometer with 633 nm laser was used to investigate the vibration spectra from the samples.

**Results and Discussion**

Vibrational Raman peaks which correspond to chemical interactions and hybridization between pDNA and cDNA were observed from both RS and SERS measurements. A clear difference in Raman response can be seen on different surfaces. For Au thin-film, Raman peaks of phosphate backbone (PO ^-^) and adenine- (A), guanine- (G), thymine- (T) and cytosine- (C) nucleobases were observed to verify the hybridization of 1 nM *secY* cDNA. For Au-Ag NPs, the signal-to-noise ratio of SERS peaks after 1 μM *lipL32* cDNA hybridization is more prominent than the RS peaks. The characteristic of SERS intensity decreases with the presence of cDNA after hybridization.

**Conclusion**

The vibrational Raman spectra from *Leptospira* DNA was successfully detected via Raman spectrometer via RS and SERS techniques. SERS exhibits more intense DNA hybridization peaks in comparison to RS, thus offering better detection sensitivity.

**Acknowledgment:** Authors wish to thank UTM and UMT for supporting this work. This work was supported by the Ministry of Higher Education under Fundamental Research Grant Scheme (FRGS/1/2021/STG07/UTM/02/4) and Fundamental Research Grant Scheme for Research Acculturation of Early Career Researchers (RACER/1/2019/TK05/UMT//1).

## O-10 Performance evaluation of nested polymerase chain reaction (nPCR), light microscopy and *Plasmodium falciparum* histidine rich protein 2 rapid diagnostic test (*pfhrp*-2 RDT) in the detection of falciparum malaria in Akure, Nigeria

### Oluwaseun Bunmi Awosolu^1, 2^, Zary Shariman Yahaya^1^ and Meor Termizi Farah Haziqah^1^

#### ^1^School of Biological Sciences, Universiti Sains Malaysia, 11800 USM, Penang, Malaysia; ^2^Department of Biology, Federal University of Technology, Akure, Nigeria

##### **Correspondence:** Oluwaseun Bunmi Awosolu (obawosolu@student.usm.my)

From The International Conference on Molecular Diagnostics & Biomarker Discovery 2022 (MDBD 2022)

Penang, Malaysia. 11 – 13 October 2022

**Background**

Malaria remains a serious public health problem worldwide. In order to ensure early and accurate malaria diagnosis, the World Health Organization recommended confirmatory parasitological diagnosis of malaria by microscopy and malaria Rapid diagnostic test (RDT) prior antimalarial administration and treatment. This study was designed to evaluate the performance of nested polymerase chain reaction (nPCR), light microscopy and *Plasmodium falciparum* histidine rich protein 2 rapid diagnostic test (*pfhrp*-2 RDT) in the detection of falciparum malaria in Akure, Nigeria.

**Methodology**

A cross-sectional and hospital-based study involving 601 febrile volunteered participants was conducted in Akure, Nigeria. Approximately 2-3 mL venous blood sample was obtained from each study participant for standard parasitological confirmation by microscopy and RDT. Thick and thin films were prepared and viewed under the light microscope for parasite density quantification and species identification, respectively. Dry blood spot (DBS) samples were prepared on 3MM Whatman filter paper for molecular analysis through nested polymerase chain reaction (nPCR).

**Results and discussion**

The overall prevalence by microscopy, RDT and nPCR were 64.89% (390/601), 65.7% (395/601) and 67.39% (405/601), respectively. Obviously, the performance efficacy of microscopy was significantly higher than RDT considering PCR as the gold standard. The estimates of sensitivity, specificity, Positive Predictive Value (PPV), Negative Predictive Value (NPV), accuracy, Youden’s j index of microscopy and RDT were 96.30, 100.00, 100.00, 92.89, 97.50, 0.963 and 95.06, 94.90, 97.47, 90.29, 95.01, 0.899, respectively. Similarly, RDT recorded higher false negativity compared microscopy (4.94% vs 3.70%). A near perfect agreement was reported between microscopy and PCR, and between RDT and PCR with Cohen’s kappa value (k) of 0.94 and 0.88, respectively.

**Conclusions**

This study revealed that RDT and microscopy continue to remain highly efficacious, though lower than PCR. Thus, while RDT continues to complement microscopy as the gold standard in high malaria-endemic settings, the application of PCR for constant evaluation and monitoring of RDT and microscopy is highly imperative to inform appropriate policy on malaria diagnosis and interventions.

## O-11 Association of single nucleotide polymorphisms on iron- regulating genes with iron metabolising parameters

### Liew Jin Rou^1^, Loo Keat Wei^2, 3^ and Teh Lai Kuan^1,3^

#### ^1^Department of Allied Health Sciences, Faculty of Science, Universiti Tunku Abdul Rahman, Perak, Malaysia; ^2^Department of Biological Sciences, Faculty of Science, Universiti Tunku Abdul Rahman, Perak, Malaysia; ^3^Centre of Biomedical and Nutrition Research, Faculty of Science, Universiti Tunku Abdul Rahman, Perak, Malaysia

##### **Correspondence:** Teh Lai Kuan (tehlk@utar.edu.my)

From The International Conference on Molecular Diagnostics & Biomarker Discovery 2022 (MDBD 2022)

Penang, Malaysia. 11 – 13 October 2022

**Background**

Anaemia is a condition with haemoglobin concentration below the established cut-off level of 12 g/dL and insufficient to meet individual’s physiological demands of oxygen. This condition has become a worldwide public health problem. Other than gender, family history, physiological condition, dietary intake, genetic factors contribute to the development of anaemia to a significant extent. In Malaysia, the prevalence of anaemia was up to 13.8% and more than half of the cases were due to iron deficient. Thus, this study is to elucidate the underlying factors in leading to anaemia including the association of SNPs on iron- regulating genes to iron-metabolising parameters and its predisposition to anaemia.

**Methodology**

A total of 183 subjects aged between 18 to 35 were recruited at Universiti Tunku Abdul Rahman with informed consent. Anthropometric measurement and haemoglobin (Hb) level were measured. Demographic data, family history and current physiological condition were self-declared by respondents through a questionnaire. A volume of 6 mL of venous blood was taken for DNA extraction while the isolated plasma and serum were used for detection of hepcidin and serum iron concentration. Eight genetic variants from 4 genes (rs10414846, rs10421768, rs855791, rs4820268, rs1799852, rs12769, rs1799945 and rs1800562) were genotyped using tetra primer- ARMS PCR. Statistical analysis was done using SPSS version 22.

**Results and Discussion**

The anaemia prevalence rate in this study population was found to be 14.75% (27/183). Women were found to have lower haemoglobin, hepcidin and serum iron level compared to men. Individuals currently menstruating or having the experience of menorrhagia presented with lower haemoglobin, hepcidin as well as serum iron. A lower hepcidin level is necessary to increase iron absorption to replenish iron loss through menstruation. Higher body mass index (BMI) was associated significantly with higher haemoglobin level, probably due to higher dietary iron intake among overweight and obese individuals. Individuals with a family history of anaemia presented with lower haemoglobin levels. The genetic variant of *TF* gene (rs12769) was associated with higher hepcidin and serum iron level while homozygous A allele in rs10421768 was associated with higher serum iron level. Other genetic variants were found to have no association with the iron-metabolising parameters that were analysed.

**Conclusion**

Anaemia is a multifactorial disease which could be due to gender, dietary intake, physiological condition as well as genetic factors. SNP in *TF* and *HAMP* gene modulates the hepcidin concentration as well as serum iron level. Findings can be further concluded in a larger sample size.

**Acknowledgement:** The work was funded by UTAR Research Fund (IPSR/RMC/UTARRF/2020-C1/T03) from Universiti Tunku Abdul Rahman, Malaysia.

## O-12 Stable expression of anti-BmR1 IgG4 antibody

### Jacqueline Kar Kei Mark and Gee Jun Tye

#### Institute for Research in Molecular Medicine (INFORMM), Universiti Sains Malaysia, Penang, Malaysia

##### **Correspondence:** Gee Jun Tye (geejun@usm.my)

From The International Conference on Molecular Diagnostics & Biomarker Discovery 2022 (MDBD 2022)

Penang, Malaysia. 11 – 13 October 2022

**Background**

Lymphatic filariasis is a parasitic disease and one of the most disabling in the world. Thus, WHO called for elimination of this disease as a public-health problem in 1997. Diagnostic kits were made and distributed worldwide to address this problem, and one of the kits is the Brugia Rapid test (Reszon Diagnostic International Sdn. Bhd., Malaysia) that uses recombinant Brugia malayi antigen BmR1 which is highly specific and sensitive for antifilarial igG4 antibodies in patients with brugian filariasis. In the manufacturing of rapid tests, quality control (QC) is necessary to ascertain the reactivity and specificity of the tests [1]. An anti-BmR1-IgG4 antibody which was synthesized and verified as a QC reagent was produced through transient expression [2]. However, transient expression has its downsides of being laborious, having low productivity and high cost in the long term as compared to stable expression which requires much lower amount of plasmid DNA and act as a renewable source of expressing cells. Thus, this study investigates the conversion of transient to stable expression of the antibody.

**Methodology**

Stable cell lines were generated using Flp-In system and Flp-In 293 cell line (Thermo Fisher). DNA sequence of anti-BmR1 IgG4 was cloned into pcDNA5/FRT, which was then co- transfected with pOg44 into Flp-In 293 cell line using Lipofectamine 3000 (Thermo Fisher). True transfectants were selected based on Hygromycin B resistance and expanded to make cell stocks and working cells for antibody expression. Protein A affinity chromatography was used to purify the supernatant, which contained the protein, after the cells had been harvested. The respective recombinant antibodies were analysed qualitatively using

Western Immunoblot, as well as quantitively at 280nm using a nanodrop spectrophotometer.

**Results and Discussion**

Western immunoblot using anti-BmR1 IgG4 recombinant antibodies showed bands at the approximate molecular masses at 69.3kDa [2]. In 10 days, 1cm^2^ of cell culture surface area (approximately 1.5 x 10^5^ of cells) expresses up to 0.776μg of antibody. Transient or stable expression systems should be chosen based on throughput, the number and quality of resources required, and turnaround time.[3]. Stable cells are favoured for large-scale protein production because they offer high yields and reliable quality of no batch-to-batch variation. Site-specific recombination has the ability to consistently produce stable cells with high yields. The total surface area of a planar vessel can be increased to easily increase the stable cell pool.

**Conclusion**

Although transient system may be apt for initial studies, mass production of antibody would require a stable expression system. The stably expressed antibody functions similarly to its transiently expressed counterpart. The Flp-In system is able to stably express the anti-BmR1 IgG4 antibody consistently and sustainably in an infinite manner, saving cost long-term.

**References**

[1] Rahmah N, et al. Multicentre laboratory evaluation of Brugia Rapid dipstick test for detection of brugian filariasis. Trop Med Int Heal. 2003, 8:895–900.

[2] Rahumatullah A, et al. Applications of recombinant monoclonal antibodies against filarial antigen proteins. Am J Trop Med Hyg. 2020, 102:578–581.

[3] Hunter M, et al. Optimization of Protein Expression in Mammalian Cells. Curr Protoc Protein Sci. 2019, 95:1–28.

## O-13 The role of Pp4R1 in T leukemic cell’s survival

### Maryam Behjat and Mirna Mourtada-Maarabouni

#### School of Life Science, Keele university, Newcastle-under-Lyme, United Kingdom

##### **Correspondence:** Mirna Mourtada-Maarabouni (m.m.maarabouni@keele.ac.uk)

From The International Conference on Molecular Diagnostics & Biomarker Discovery 2022 (MDBD 2022)

Penang, Malaysia. 11 – 13 October 2022

**Background**

Reversible phosphorylation is one of the critical post-translational cell events, controlled by protein kinases and protein phosphatases. Protein kinases are involved in many human diseases including cancers. Protein phosphatases regulate cellular events by reversing protein kinases activity. The serine/threonine protein phosphatase 4 (PP4) is an essential protein for nucleation, growth, and stabilization of microtubules in centrosomes/spindle bodies during cell division and survival of T cells. It consists of catalytic subunit (PP4c) interacting with four different regulatory subunits (PP4R1, PP4R2, PP4R3 and PP4R4). Previous studies showed that PP4c has an important tumour suppressor function and plays crucial role in the control of cell fate in leukemic T-cells and untransformed human peripheral blood T-cells. The present study investigates the role of the protein phosphatase 4 regulatory subunit 1 (PP4R1) in leukaemia.

**Methodology**

Two different leukemic T cell lines Jurkat and CEM-C7 were transfected with two different PP4R1 specific siRNAs to study the effects of PP4R1 down-regulation on cell survival or with a plasmid encoding PP4R1 (pcDNA3.1-PP4R1) to investigate the effects of PP4R1 up-regulation.

**Results and Discussion**

Western blot analysis confirmed both down-regulation and over-expression of PP4R1. Reduction of PP4R1 protein levels was associated with an increase in the number of both total and viable cells. Increased levels of PP4R1 led to a significant decrease in the total and viable cell number, increase in basal apoptosis and cell cycle arrest in G1.

**Conclusion**

Overall, the results show that together with PP4c, PP4R1 regulates cell survival and growth of Jurkat and CEM-C7 leukemic T cells and propose that it could play an important role in maintaining the balance between cancer cell survival and death and might clarify the distinct pathological mechanism of leukaemia.

**Reference**

Mourtada-Maarabouni, M., Gwyn, T.W. (2009) Protein phosphatase 4 regulates apoptosis in leukemic and primary human T-cells. Leukaemia Research 33, 1539-1551

## O-14 X-chromosome wide association in Thai SLE patients

### Krisana Jaiwan^1^, Yao Lei^2^, Manon Boonbangyang^3^, Punna Kunhapan^4^, Nusara Satproedprai^4^, Surakameth Mahasirimongkol^4^, Prapaporn Pisitkun^5^, Nattiya Hirankarn^6^, Wang Yong Fei ^2^ and Pattarin Tangtanatakul ^1^

#### ^1^Department of Transfusion Medicine and Clinical Microbiology, Faculty of Allied Health Sciences, Chulalongkorn University, Bangkok, Thailand; ^2^Department of Paediatrics and Adolescent Medicine, The University of Hong Kong, Hong Kong, China; ^3^National Biobank of Thailand (NBT), National Science and Technology Development Agency (NSTDA), Pathum Thani, Thailand; ^4^Department of Medical Sciences, Ministry of Public Health, Nonthaburi, Thailand; ^5^Section of Translational Medicine, Faculty of Medicine, Mahidol University, Ramathibodi Hospital, Bangkok, Thailand; 6Centre of Excellent in Immunology and Immune-Mediated Diseases, Department of Microbiology, Faculty of Medicine, Chulalongkorn University, Bangkok, Thailand

##### **Correspondence:** Pattarin Tangtanatakul (Pattarin.T@chula.ac.th)

From The International Conference on Molecular Diagnostics & Biomarker Discovery 2022 (MDBD 2022)

Penang, Malaysia. 11 – 13 October 2022

**Background**

A previous study has shown that the genetic components especially in X chromosome contributed to female-biased found in Systematic Lupus Erythematosus (SLE) patients [1]. A copy of X chromosome is relatively correlated with SLE development [2]. Due to the global hypomethylation or defective in X-Chromosome Inactivation (XCI), [2] the studies found that 23% of genes are able to escape from this mechanism leading to X-linked gene over-expression found in SLE [3]. Genetic variations are deviated in different populations and may influence the incidence and mortality rate. Therefore, our study aims to investigate a global variant on X chromosome in Thai population. This might help to address the high female to male incidence ratio found among SLE patients.

**Methodology**

All procedures are approved by the ethics committee at Faculty of Medicine, Chulalongkorn University (EC. 590223). Using Asian Screening Array results from previous study [4], We conducted genome-wide association study (GWAS) on X chromosome from 892 SLE samples compared with healthy controls data (n = 1,683). Low quality samples and bad SNP markers were excluded by quality control processes (QC). Inflation factors after QC process are 1.008. Next, haplotype estimating was completed by SHAPEIT to pre-phasing the SNPs before imputation step by IMPUTE v2.3.2 software using -chrX function. SNPs association analysis were analyzed using plink v1.9 with logistic correlation approach.

**Results and Discussion**

From our analysis, we characterized several known independent susceptibility SNPs in Thai SLE patients which include rs1059702 (G>A) (OR = 0.63, p-value = 9.30x10^-9^) located at *IRAK1- MECP2- TMEM187* region. Interestingly, the risk loci rs777448097 in *TLR7* (p-value 1.36 x 10^-5^, OR = 0.77) were found in Thai population while it was absent in Hong Kong and Central Chinese background [1]. Furthermore, we found novel suggestive signal (rs14229594, C>T) on *GAB3-CTAG1A loci* (OR = 0.71, P = 2.14x10^5^). This variant showed higher effect size in female SLE patients and located within the enhancer region of CD14+ Monocytes (ENCODE database).

**Conclusion**

In the present study, we report a number of known SLE susceptible loci, specifically in the Thai population. Although the suggestive novel risk loci on *GAB3-CTAG1A* regions have been identified, further studies are required to validate this result. Our finding may help address the female biased phenomenon associated with SLE in the Thai population.

**Acknowledgement:** This research was funded by Chulalongkorn University (ReinUni_65_01_37_02), Research Assistant Scholarship Foundation (GCUGE1725631001M) and partly supported by The Asahi glass foundation (EXG64394).

**References**

[1] Zhang H, et al. Meta-analysis of GWAS on both Chinese and European populations identifies GPR173 as a novel X chromosome susceptibility gene for SLE. Arthritis Res Ther. 2018 May 3;20(1):92.

[2] Tukiainen, T., Villani, AC., Yen, A. et al. Landscape of X chromosome inactivation across human tissues. Nature 2017, 550: 244–248.

[3] Tiao, J., et al., Using the American College of Rheumatology (ACR) and Systemic Lupus International Collaborating Clinics (SLICC) criteria to determine the diagnosis of SLE in patients with SCLE. J Am Acad Dermatol, 2016. 74(5): p. 862-9.

## O-15 The effect of mesenchymal stem cells-mediated macrophages activation on breast cancer progression

### Nur Ramziahrazanah Jumat, Muhammad Amir Yunus, Badrul Hisham Yahaya and Rafeezul Mohamed

#### Department of Biomedical Science, Advanced Medical and Dental Institute, Universiti Sains Malaysia, Penang, Malaysia

##### **Correspondence:** Rafeezul Mohamed (rafeezul@usm.my)

From The International Conference on Molecular Diagnostics & Biomarker Discovery 2022 (MDBD 2022)

Penang, Malaysia. 11 – 13 October 2022

**Background**

Breast cancer (BC) is the second most leading cause of cancer- related death among women worldwide and represent about 11.7% of all new diagnosed cancer cases in 2020 [1]. High population of tumor-associated macrophages (TAM) in tumor microenvironment (TME) which display M2-type macrophages promotes BC metastasis and correlates with poor clinical prognosis in BC patients. Reprogramming TAM into M1-type macrophages that capable of killing tumor cells has emerged as a favorable therapeutic target in BC. Mesenchymal stem cells (MSC) can stimulate immunomodulatory changes and skew naïve macrophages (M0) to M1-type macrophages [2]. Therefore, this study attempted to evaluate MSC-macrophages crosstalk on BC progression.

**Methodology**

THP-1 cell was incubated with PMA for 48 hours to differentiate into M0. M0 were co-cultured with MSC for 30 hours. Conditioned medium (CM) was collected to evaluate M1- and M2-type macrophage related cytokine while total RNA was extracted from co-cultured cells and then synthesised into cDNA to determine IRF-4 and IRF-5 mRNA gene expression using RT- PCR. Finally, MDA-MB-231 cells were cultured with CM of respective treatments for 24, 48 and 72 h to evaluate their cell viability using MTT assay.

**Results and Discussion**

Results showed that UC-MSC significantly increased IRF-4 expression in M0 and M0+LPS compared to M0 alone but no effect on IRF-5. In addition, UC-MSC did not influence the secretion of IL-10 and TNF-α in the supernatant of M0 and M0+LPS. Moreover, CM from M0, M0+UC-MSC, M0+LPS and M0+LPS+UC-MSC significantly reduced the proliferation of MDA-MB-231 cells at 72 h compared with 24 h treatment respectively. The results indicated that UC-MSC induced the polarisation of M0 into M2-type phenotype as IRF-4 regulates M2-type macrophage polarisation. M0 secretory products suppressed ER- breast cancer cells (MDA-MB-231) as observed in a previous study (3). UC-MSC and LPS may also stimulate the M0 to secrete a secretome that contains anti-cancer properties that hinders the proliferation of triple negative BC cells. This finding is consistent with a prior study that showed umbilical cord matrix derived MSC-CM inhibited the cell viability of BC cells.

**Conclusion**

Our findings demonstrate that UC-MSC may polarise naïve macrophages into M2-type macrophages. UC-MSC and LPS work in synergy in stimulating M0 macrophages to secrete anti- cancer products that suppress the growth of triple negative breast cancer cells.

**Acknowledgement:** The work was funded by the Research University Individual Grant Scheme (1001.CIPPT.8012328) from Universiti Sains Malaysia.

**References**

[1] Sung H., et al., Global cancer statistics 2020: GLOBOCAN estimates of incidence and mortality worldwide for 36 cancers in 185 countries. CA CANCER J CLIN 2021, 0, 1–41.

[2] Vasandan AB., et al. Human Mesenchymal stem cells program macrophage plasticity by altering their metabolic status via a PGE2-dependent mechanism. Sci Rep. 2016, 6:38308.

[3] Song W, et al. Conditioned medium from stimulated macrophages inhibits growth but induces an inflammatory phenotype in breast cancer cells. Biomed Pharmacother. 2018, 106:247-254.

## O-16 Therapeutic properties of Malaysian stingless bee pollen and its protective effect against DNA damage

### Nurdianah Harif Fadzilah and Wan Adnan Wan Omar

#### Advanced Medical and Dental Institute, Universiti Sains Malaysia, Bertam, Kepala Batas, Penang, Malaysia

##### **Correspondence:** Nurdianah Harif Fadzilah (nurdianaharif@usm.my)

From The International Conference on Molecular Diagnostics & Biomarker Discovery 2022 (MDBD 2022)

Penang, Malaysia. 11 – 13 October 2022

**Background**

Reactive Oxygen Species (ROS) can disturb cellular metabolism and damage cellular biomolecules, which could lead to DNA damage. Natural compounds including bee-collected pollen contain nutrient antioxidants that have therapeutic properties and protective effects against ROS. Bee pollen is known as a complete food since the food energy produced is relatively high, thus it serves as a source of nutrients for adult bees and larvae [1]. Studies demonstrated that 70% of bee pollen compositions are biologically active and exhibit numerous benefits including nutrition, cardioprotection, hepatoprotection, immunostimulant, antioxidation, anticarcinogen, antibacterial, antiosteoporosis, antiprostatitis, anti-anaemia, anti-ageing, and anti-inflammatory [2, 3]. This study aims to investigate the protective effect of stingless bee pollen against DNA damage.

**Methodology**

Bee pollen ethanolic extracts (BPEs) were prepared from three stingless bee species commonly domesticated in Malaysia: i.e., *Tetrigona apicalis, Heterotrigona itama*, and *Geniotrigona thoracica.* The methodologies used in this study were spectrophotometric method for total phenolic/flavonoid content and antioxidant activities, HPLC and GC-MS chromatographic techniques for phenolic compounds identification, trypan blue exclusion assay for antiproliferation test, and comet assay for DNA damage activities. MCF-7, MCF-10A and HT29 cell lines were used in this study.

**Results and Discussion**

In antioxidant assay, the result showed that BPE from Malaysian stingless bee species contained different phenolic and flavonoid contents, with each extract possessing different amounts of antioxidant activity. *G. thoracica* BPE possessed the highest capacity to neutralize DPPH radicals compared to *T. apicalis* and *H. itama*. HPLC and GC-MS analysis detected various polyphenol compounds and chemical groups in each species. The chemical compounds found in BPE have lots of biological activities with antioxidative potential to be explored as health supplements and medical treatment. In antiproliferation assay, *G. thoracica* exhibited the highest therapeutic index (TI=3.12), with the EC_50_ of 0.5 mg/mL in HT-29 cells. With the therapeutic potential, stingless bee pollen may provide a preventive function against ROS and the development of many lifestyle diseases. Analysis of DNA damage activity at 24 h of *G. thoracica* treatment showed a significant decrease in H_2_O_2_-induced DNA damage compared to the untreated cells (63.82% + 2.46 vs. 90.86% + 0.68) (p<0.01). Similarly, a significant reduction was also seen with the supplementation of caffeic acid (49.05% + 4.23) and quercetin (43.98% + 3.77) (p<0.01). More significant reduction of DNA damage was observed at 72 h of *G. thoracica BPE* (20.49% + 0.73), caffeic acid (5.65% + 0.35), and quercetin treatment (7.58% + 0.32) (p<0.01) on H_2_O_2_-exposed HT-29 cells. Due to its strong antioxidant activity, *G. thoracica* BPE gave a protective effect against H_2_O_2_-induced DNA damage.

**Conclusion**

Results of the present study discovered that Malaysian stingless bees, particularly *G. thoracica* species have a genoprotective effect that can protect cells from H_2_O_2_-induced oxidative DNA damage. Even though the exact mechanism concerning the protective effects of BPE is still unconfirmed, the bioactive phytochemicals and antioxidant capacity are believed to be responsible for the suppression of oxidative DNA damage and may confer protection from genetic instability.

**References**

[1] Kocot, J., et al. Antioxidant potential of propolis, bee pollen, and royal jelly: Possible medical application. Oxidative Medicine and Cellular Longevity. 2018, 7074209.

[2] Yang, K., et al. Characterization of chemical composition of bee pollen in China. Journal of Agricultural and Food Chemistry. 2013, 61(3):708–718.

[3] Rzepecka-Stojko, et al. Polyphenol content and antioxidant activity of bee pollen extracts from Poland. Journal of Apicultural Research. 2015, 54(5):482–490.

## O-18 Acute and sub-chronic toxicological evaluation of probiotic strain *Lactobacillus rhamnosus* GG in Sprague Dawley rats

### Venkata Kanthi Vaishnavi Vedam^1, 4^, Subramani Parasuraman^2^, Urmila Banik^3^ and Arun Kumar Adhikary^4^

#### ^1^Department of Oral Pathology, Faculty of Dentistry, AIMST University, Bedong, Semeling Kedah Darul Aman, Malaysia; ^2^Department of Pharmacology, Faculty of Pharmacy, AIMST University, Bedong, Semeling Kedah Darul Aman, Malaysia; ^3^Department of Pathology, Faculty of Medicine, AIMST University, Bedong, Semeling Kedah Darul Aman, Malaysia; ^4^Department of Microbiology, Faculty of Medicine, AIMST University, Bedong, Semeling Kedah Darul Aman, Malaysia

##### **Correspondence:** Venkata Kanthi Vaishnavi Vedam (vaishnavivedam@gmail.com)

From The International Conference on Molecular Diagnostics & Biomarker Discovery 2022 (MDBD 2022)

Penang, Malaysia. 11 – 13 October 2022

**Background**

Lactic acid bacteria are one of the major groups of gastrointestinal bacteria in healthy humans. A strong history of commensal status and long-term usage without any obvious adverse effects has classified them as ‘Generally Recognized as Safe’. In recent years, bacteriotherapy using probiotics as therapeutic agents has shown to be a promising emerging method for evasion of infectious diseases. *Lactobacillus* spp. as probiotics has been questioned for its safety due to recently reported unexpected reactions. These include *Lactobacillus*– associated systemic infections, metabolic reactions, immune disorders and gene transfer *etc.*]. Among the probiotics, opinions proposed by several organizations between countries remain still contradictory. Although *Lactobacillus* spp. as probiotics have been classified safe, *Lactobacillus rhamnosus* strain GG requires further surveillance with additional studies.

**Methodology**

Acute and sub-chronic toxicity studies of probiotic strain *Lactobacillus rhamnosus* GG in Sprague-Dawley rats (180 ± 20 g) as per OECD TG 423 and 407, respectively. Acute oral toxicity study was conducted by the administration of 1 × 10^8^, 1 × 10^9^ and 1 × 10^10^ cfu/ ml of *L. rhamnosus* (n = 3/ group). The animals were observed for their behavioral, neurological and autonomic functions continuously for 24 h and monitored 14 days thereafter for mortality. Sub-chronic toxicity study was conducted by the administration of 1 × 10^6^, 1 × 10^7^ and 1 × 10^8^ cfu/ ml of *L. rhamnosus* once daily for a 28 days period (n = 12/ group). During the study period, the animals were monitored for changes in body weight and behavioral functions. The blood sample was collected on day 14 and 28, used for biochemical and hematological parameters analysis. At the end of study the animals were sacrificed and pivotal organs were collected and used for histopathological analysis.

**Results and Discussion**

In acute toxicity studies, *L. rhamnosus* did not show any changes in behavioral, neurological and autonomic functions, and mortality. In sub-chronic study, *L. rhamnosus* did not show any significant changes in body weight gain, organ weight analysis and behavioral functions. *L. rhamnosus* at 1 × 10^6^ cfu/ml did not show any significant changes in biochemical and hematological parameters when compared with the control. Whereas *L. rhamnosus* at 1 × 10^7^ and 1 × 10^8^ cfu/ml showed dose-depended changes in elevated aminotransferase levels when compared with the control. In histopathological analysis, mild degeneration of hepatic cells and nephrons were observed in *L. rhamnosus* at 1 × 10^8^ cfu/ml administered group when compared with the control.

**Conclusion**

The probiotic *L. rhamnosus* GG did not show any significant toxic effect at 1 × 10^6^ cfu/ml and exhibited mild-to-moderate toxic effects at the dese levels of 1 × 10^7^ cfu/ml and 1 × 10^8^ cfu/ml. Pre-clinical toxicity testing helps to calculate “No Observed Adverse Effect Level [NOAEL]”.

**References**

[1] OECD Guidelines for the Testing of Chemicals, Section 4: Test No. 423: Acute Oral toxicity - Acute Toxic Class Method.

[2] OECD Guidelines for the Testing of Chemicals, Section 4: Test No. 407: Repeated Dose 28-day Oral Toxicity Study in Rodents.

[3] Parasuraman S. Toxicological screening. J Pharmacol Pharmacother. 2011;2(2):74-79.

## O-19 In-depth investigation of microRNA methylome signature in colorectal cancer

### Nurul Qistina Rus Bakarurraini ^1^, Rashidah Baharudin ^2^, Imilia Ismail ^3^, Nadiah Abu ^2^, Siti Aishah Sulaiman ^2^, Learn-Han Lee ^4^ and Nurul Syakima Ab Mutalib ^1, 4, 5^

#### ^1^Faculty of Applied Sciences, Universiti Teknologi Mara (UiTM), 40450 Shah Alam, Selangor, Malaysia; ^2^UKM Medical Molecular Biology Institute (UMBI), Universiti Kebangsaan Malaysia, 56000 Cheras, Kuala Lumpur, Malaysia. ^3^School of Biomedicine, Faculty of Health Sciences, Universiti Sultan Zainal Abidin (UniSZA), 21300, Terengganu, Malaysia. ^4^Novel Bacteria and Drug Discovery Research Group, Microbiome and Bioresource Research Strength, Jeffrey Cheah School of Medicine and Health Sciences, Monash University Malaysia, 47500 Subang Jaya, Selangor, Malaysia; ^5^Faculty of Health Sciences, Universiti Kebangsaan Malaysia, Kuala Lumpur 50300, Malaysia

##### **Correspondence:** Nurul Syakima Ab Mutalib (syakima@ppukm.ukm.edu.my)

From The International Conference on Molecular Diagnostics & Biomarker Discovery 2022 (MDBD 2022)

Penang, Malaysia. 11 – 13 October 2022

**Background**

Colorectal cancer (CRC) is one of the top causes of cancer-related deaths worldwide. Despite substantial breakthroughs in diagnostic services and patient care, various gaps remain to be filled, ranging from early detection to the identification of prognostic indicators, effective therapy for metastatic illness, and the implementation of personalised treatment methods. MicroRNAs, which are small non-coding RNAs, are unregulated in CRC and play an important role in its onset and progression. Despite this, microRNA research has traditionally relied on expression levels to assess biological importance [1]. The precise mechanism underlying microRNA dysregulation in cancer has yet to be determined, however multiple studies have shown that epigenetic mechanisms, notably DNA methylation, play essential roles in the regulation of microRNA production. Thus, we intend to investigate the miRNA methylome patterns and explore their roles CRC.

**Methodology**

Fifty-four pairs (n=108) of CRC and the respective adjacent normal tissues were collected from the UKM Medical Center, Malaysia. Methylation profiling was performed using the Infinium Human Methylation 450K beadchip, which covers 485,577 CpG dinucleotide sites distributed over the whole genome. The raw IDAT files were exported from the scanner, and quality control was performed using Genome Studio and were further analysed using the ChAMP R package.

**Results and Discussion**

We obtained 933 miRNA probes for the downstream analysis. These probes were further classified as hypermethylated or hypomethylated based on the absolute average β value difference (Δβ) at ≥0.2 between CRC and normal adjacent tissues. A total of 230 probes were identified. Distribution of hypomethylated miRNAs with respect to genomic regions revealed more than half of the miRNA probes were located in the TSS1500 (n = 130; 58%), followed by TSS200 (n=55; 24%) and body (n=40; 18%). On the other hand, the distribution of hypermethylated miRNA probes is almost equal in TSS200 (n=3; 60%) and TSS1500 (n=2; 40%). Meanwhile, categorization based on CpG islands (CGIs), 225 probes were hypomethylated and five probes were hypermethylated. The five hypermethylated miRNA probes were MIR1180_cg24553547, MIR124-3_cg02650317, MIR34B_cg22879515, MIR124-3_cg15699267 and MIR137_cg22333214. Among these, MIR1180_cg24553547 showed the most significant differentially hypermethylated delta β-value. Although atypical methylation status has been documented in tumour samples, the methylation profile of miR-1180, which is located on chromosome 17, has yet to be thoroughly explained in CRC. Prior research demonstrated a good association between hypermethylation of the miR-1180 gene and downregulation of its expression in tumour tissue when looking at its expression level in malignancies. Unlike other miRNAs, miR-1180 functions as both an oncogene and a tumour suppressor. Tan et al. [2] and Zhou et al. [3] previously demonstrated the carcinogenic activity of this miRNA in hepatocellular carcinoma by directly targeting both OTUD7B and TNIP2 to drive cell proliferation and apoptosis resistance.

**Conclusion**

Our findings imply the existence of new pathways involving methylation alterations impacting miRNA genes that control CRC development. The new knowledge from this study can be utilized for personalized health diagnostics, disease prediction, and monitoring of treatment.

**Acknowledgements:** This research was funded by Ministry of Higher Education Malaysia, grant number FRGS/1/2020/SKK0/UKM/02/11.

**References**

[1] Baharudin, R, et al. Methylome Signature and Their Functional Roles in Colorectal Cancer Diagnosis, Prognosis, and Chemoresistance. Int. J. Mol. Sci. 2022, 23, 1-16.

[2] Tan, G, et al. MiR-1180 promotes apoptotic resistance to human hepatocellular carcinoma via activation of NF-kappaB signaling pathway. Sci Rep. 2016, 6, 1-11.

## O-20 Identification of LRRC17 as colonic fibroblast activation marker and its potential role in colorectal cancer progression

### Sahira Syamimi Ahmad Zawawi and Marahaini Musa

#### Human Genome Centre, School of Medical Sciences, Universiti Sains Malaysia, Kelantan, Malaysia

##### **Correspondence:** marahaini.musa@usm.my)

From The International Conference on Molecular Diagnostics & Biomarker Discovery 2022 (MDBD 2022)

Penang, Malaysia. 11 – 13 October 2022

**Background**

Stromal fibroblast is linked with poor prognosis of colorectal cancer (CRC) [1]. Crosstalk between cancer-associated fibroblasts (CAFs) and cancer cells facilitated by growth factors drives the malignant progression. CAFs can be identified using conventional markers such as alpha-smooth muscle actin (α-SMA) although their expression can be heterogenous. Leucine Rich Repeat Containing 17 (LRRC17) is proposed to be an emerging biomarker to specifically identify activated fibroblasts and CAFs. This study was aimed to verify LRRC17 expression in different colonic fibroblasts and further characterize LRRC17 in CRC. This may provide insights on the underlying mechanisms and verify its potential as a specific marker for future prognostic purposes [2].

**Methodology**

LRRC17 and α-SMA expressions were analyzed using immunofluorescence staining on CCD-112CoN (MF cell line) and primary fibroblast lines derived from colorectal tumor and normal adjacent colon tissues (denoted as normal fibroblasts). SW620 (CRC epithelial cell line) was used as control. The cells were cultured under different conditions; a) serum-free medium (DMEM alone), b) DMEM + 10% fetal bovine serum (complete medium), c) conditioned medium (CM) of SW620, and d) DMEM + transforming growth factor β1 (TGF-β1). Fluorescence intensity was assessed via ImageJ 1.53k. Treated cells proliferation was assessed via MTT assay. All experiments were conducted in duplicates.

**Results and Discussion**

Positive LRRC17 and α-SMA expressions were observed in all fibroblast groups and CCD-112CoN except SW620. However, different co-localization patterns were shown between the proteins in treated cells. Both proteins expression was greater in complete medium and TGF-β1 as compared to serum free group, indicating more activated state of treated fibroblasts mimicking CAF property. Interestingly, higher LRCC17 expression whereas lower α-SMA expression in CM SW620 for primary cell lines. This may highlight the cancer secretome effects which trans-differentiate fibroblasts or change their molecular property. Different expression patterns seen in the present study may indicate different pathways involved in the regulation of these markers, suggesting further study. From MTT assay, highest proliferation was found in fibroblasts treated with complete medium. It was proposed that growth factors in serum support the cell growth [3].

**Conclusion**

LRRC17 can potentially serve as biomarker to identify and characterize CAFs of CRC.

**Acknowledgement:** The study was funded by the Short-Term Research Grant (304.PPSP.6315409) from Universiti Sains Malaysia.

**References**

[1] Takatsuna, et al. Myofibroblasts of the muscle layer stimulate the malignant potential of colorectal cancer. Oncology Reports. 2016, 36(3): 1251-1257.

[2] Hsia, et al. Myofibroblasts are distinguished from activated skin fibroblasts by the expression of AOC3 and other associated markers. Proc Natl Acad Sci USA. 2016; 113(15): E2162-E2171.

[3] Gstraunthaler G: Alternatives to the use of fetal bovine serum: serum-free cell culture. ALTEX. 2003; 20(4): 275-281.

## O-21 Effect of polyphenolic-rich fraction of cornsilk (*Stigma maydis*) in streptozotocin-induced diabetic rats

### Siti Azhani, Nor Syamim, Nur Fatihah and Sabreena Safuan

#### School of Health Sciences, University Science Malaysia, Kelantan, Malaysia

##### **Correspondence:** Sabreena Safuan (sabreena@usm.my)

From The International Conference on Molecular Diagnostics & Biomarker Discovery 2022 (MDBD 2022)

Penang, Malaysia. 11 – 13 October 2022

**Background**

Diabetes is a chronic condition that arises when the pancreas does not create enough insulin or when the insulin produced is not used efficiently by the body. Hyperglycemia, or elevated blood sugar, is a frequent complication of untreated diabetes and can cause catastrophic harm to several of the body's systems, including the neurons and blood vessels. Current available drug such as metformin which is widely used to treat hyperglycemia have some side effects such as diarrhea, vomiting and lactic acidosis. Currently, there is a keen interest in studying the benefits of food waste, hence researchers started to study the benefits of natural product food waste in treating many pathological conditions. Therefore, the aim of this research is to study the effects of polyphenolic-rich fraction of cornsilk in reducing the fasting blood glucose level in diabetic-induced rats.

**Methodology**

The effect of polyphenolic-rich fraction (PRF) of corn silk (CS) extract were studied on the normal cell line Human Umbilical Vein Endothelial Cells (HUVEC) and Streptozotocin-induced diabetic rats Streptozotocin-induceddiabetic rats.

**Results and Discussion**

The proliferation assay result of PRF of CS obtained shown significant percentage of cell viability when compared to metformin (p<0.05). The effects of PRF of CS extracts in fasting blood glucose level shown no significant different between treated and untreated group (p>0.05), might be due to the 14-day duration of this study. The effects of PRF of CS on the body weight of rats also shown no significant different when compared between treated and untreated group (p>0.05).

**Conclusion**

However, the histopathological study shows promising effect of PRF of CS in treating diabetes when compared to untreated diabetic group by assessing the morphology of the glomerulus, tubules and Bowman space in kidney and the morphology of centrilobular area, hepatocytes and sinusoidal space in the liver. The long-term impact of PRF on CS in diabetic models has to be studied further by repeating the research study over a longer time period.

## O-22 Phytochemical, anti-microbial activity and anti-proliferation test against human cancer-origin cell lines using water extracts of *Momordica cochinchinensis* (Gac fruit)

### J.T Priscilla^1^, Ming-Thong Ong^1^ and Sreeramanan S.^2, 3^

#### ^1^Institute for Research in Molecular Medicine (INFORMM), Universiti Sains Malaysia, Pulau Pinang, Malaysia; ^2^School of Biological Sciences, Universiti Sains Malaysia, 11800 USM, Pulau Pinang, Malaysia; ^3^Centre for Chemical Biology, Universiti Sains Malaysia, Bayan Lepas, 11900, Pulau Pinang, Malaysia

##### **Correspondence:** Ming-Thong Ong (omt@usm.my; ong.mt1@gmail.com)

From The International Conference on Molecular Diagnostics & Biomarker Discovery 2022 (MDBD 2022)

Penang, Malaysia. 11 – 13 October 2022

**Background**

*Momordica cochinchinensis* (Gac fruit) is a seasonal tropical fruit that is widely used in Southeast Asian countries [1]. However, it’s not fully exploited in Malaysia. The major component of this fruit that has been focused on is the aril, however the pulp, peel and seed are often discarded. Therefore, this study aims to scientifically validate the presence of phytochemicals composition, determine the antioxidant, antimicrobial and anti-proliferative activities of different parts (aril, pulp and seed) of Gac fruit grown in Malaysia.

**Methodology**

The fruit (aril, pulp and seed) was macerated and extracted using water following the traditional preparation approach. A toxicity test using brine shrimp (BSLT), phytochemical tests, antioxidant, antimicrobial, antifungal and cytotoxicity cell-based assays were conducted.

**Results and Discussion**

The crude extract showed 50% mortality in brine shrimps after 24 hours and its LC_50_-value was considered moderate toxic. Qualitative phytochemical analysis revealed the presence of alkaloids, flavonoids, saponins, volatile oils, glycosides and tannins. The highest scavenging activity of water extract from the pulp with 30.1 mg GAE/g FW and 0.012 mg GAE/g FW in both DPPH and FRAP, respectively. The pulp water showed high levels of total phenolic and flavonoid content of 0.0215 mg GAE/g FW and 0.083 mg QE/g FW, respectively. Moreover, pulp water extract displayed strong antimicrobial activity with MIC values ranging from 5-20 mg/ml and MBC values of 10-20 mg/ml on certain microbial strains. Cancer origin cells MCF7, HepG2, A549, HCT116 and HT29 were found to be more susceptible with the treatment of aril and pulp water extracts with the LC_50_ at 15.41, 5.87, 22.89, 1.10 and 0.03 μg/ml while pulp at higher values such as 269.40, 328.46, 34.90, 68.07 and 21.44 μg/ml at 72 hours post-treatment compared to other tested cell lines.

**Conclusion**

The results concluded that Gac fruit from crude extracts revealed chemical constituents (alkaloids, flavonoids, saponins, volatile oils, glycosides and tannins) contributing to their antioxidant and antimicrobial activity against *E.coli, P.euroginosa, B.cereus and S.flexneri.* Further studies on the mechanism pathways need to be explored since Gac fruit showed antiproliferative activity against breast, liver, lung and two colon cancer cell lines.

**References**

[1] Chuyen H,. Gac fruit (Momordica cochinchinensis Spreng.): a rich source of bioactive compounds and its potential health benefits Int. J. Food Sci**.***,*2015*,* 50:567-577.

## O-23 Conjugates between P. *marcocarpa* aqueous extract and TiO_2_ exhibited a synergistic antimicrobial effect

### Fanne Yeoh Fern Nii^1^, Ong Ming Thong^1^, Lim Gin Keat^2^ and Srimala Sreekantan^3^

#### ^1^Institute for Research in Molecular Medicine (INFORMM), Universiti Sains Malaysia, 11800 USM, Penang, Malaysia; ^2^ School of Chemical Sciences, Universiti Sains Malaysia, 11800 USM, Penang, Malaysia; ^3^ School of Materials & Mineral Resources Engineering, Universiti Sains Malaysia, Engineering Campus, 14300 Nibong Tebal, Penang, Malaysia

##### **Correspondence:** Fanne Yeoh Fern Nii (fannegirl@gmail.com)

From The International Conference on Molecular Diagnostics & Biomarker Discovery 2022 (MDBD 2022)

Penang, Malaysia. 11 – 13 October 2022

**Background**

*Phaleria marcocarpa* (P. *marcocarpa*), also known as Mahkota Dewa is a flowering plant of Thymelaeceae family. Originated from Indonesia, it thrives in tropical climate countries such as Malaysia. It has been reported to possess anti-diabetic and anti-bacterial properties and is often brewed as tea for consumption. However, the antimicrobial activities of P. *marcocarpa* are proven to be weak and limited to limited strains of microbes. Titanium dioxide nanoparticles (TiO_2_ NPs) are semiconducting transition metals with strong antimicrobial activities. P*. marcocarpa* aqueous extract was conjugated with TiO_2_ NPs in this study to determine the synergistic antimicrobial effect of the resultant conjugate against *Escherichia coli* (E*. coli*) and *Staphylococcus aureus* (S. *aureus*).

**Methodology**

The aqueous P. *marcocarpa* fruit extract was extracted through maceration for 24 hours. The extract was filtered through Whatman® filter paper No 1 and freeze dried. TiO_2_ NPs were synthesized through the sol-gel method. The conjugation between the aqueous extract and TiO_2_ NPs were carried out at various temperatures (30°C, 45°C and 60°C). The resultant conjugate was characterized through FTIR. The antimicrobial activity test was carried out through MTT assay against *E. coli* (ATCC 25922) and *S. aureus* (ATCC 6538). The assay was carried out using a 96-well sterile microtiter plate. The concentration of extract, TiO_2_ NPs and resultant conjugate used were from 125 to 15.6 μg/mL.

**Results and Discussion**

The FTIR results showed difference between the TiO_2_ NPs and resultant conjugate around the fingerprint region. Bands at 3305, 2129, 1637, 1538 and 1370 cm^-1^ have been shown by pure TiO_2_ NPs, while bands at 3305, 2144, 1637 cm^-1^ have been shown by the extract. Additional band at 1045 cm^-1^ was shown by the resultant conjugate while additional band at 792 cm^-1^ was observed for the conjugate prepared at 60°C. The 1045 cm^-1^ band represented anhydride and alcohol stretching while the 792 cm^-1^ band represented the bending of alkene. From the antimicrobial test against E. *coli* and S. *aureus*, the conjugates prepared at 30°C, 45°C, and 60°C has minimum inhibitory concentration (MIC) of 15.6 μg/mL. However, the conjugate at 45°C showed lowest CFU/mL of 3.5 x 10^6^ for E. *coli* and 9.6 x 10^6^ for S. *aureus* when compared to TiO_2_ NPs and other conjugates (30°C and 60°C) at the same concentration. Thus, it was proven that 45°C is the optimum temperature for TiO_2_ extract conjugation in view of growth inhibition for E. *coli* and S. *aureus* as it showed lower CFU/mL as compared to the pure TiO_2_ NPs.

**Conclusion**

In conclusion, the MTT assay was effectively used at evaluating the antimicrobial activity. The resultant conjugate showed the most remarkable synergistic antimicrobial effect of MIC at 15.6 μg/mL and 45°C for both E. *coli* and S. *aureus*.

## O-24 Cytotoxicity, proliferation and migration assessment of BHMC, the curcuminoid analogue on human liver cancer cells, HepG2

### Muhammad Aminuddin Mohd Shafiee^1^, Mohd Ashraf Muhamad Asri^1^, Nurul Asyikin Mahbud^1^, Nur ‘Aqilah ‘Inani Hanapi^1^, Marwah Salaebing^1^, Zulkefley Othman^1^, Armania Nurdin^1,2^ and Sharifah Sakinah Syed Alwi^1^

#### ^1^Department of Biomedical Science, Faculty of Medicine and Health Sciences, Universiti Putra Malaysia (UPM), 43400 UPM Serdang, Malaysia; ^2^Laboratory of UPM-MAKNA Cancer Research (CANRES), Institute of Bioscience, Universiti Putra Malaysia (UPM), 43400 UPM Serdang, Malaysia

##### **Correspondence:** Sharifah Sakinah Syed Alwi (sh_sakinah@upm.edu.my)

From The International Conference on Molecular Diagnostics & Biomarker Discovery 2022 (MDBD 2022)

Penang, Malaysia. 11 – 13 October 2022

**Background**

Over the years, natural bioactive compounds were acknowledged for their cytotoxic and anticancer capabilities. Curcumin is a bioactive compound derived from the rhizomes of *Curcuma longa* known to possess various pharmacological properties e.g., inhibiting cancer growth, exerting cytotoxic and inducing apoptosis [1]. Nonetheless, curcumin has some limitations that prevent it from reaching its full potential. Due to poor bioavailability, 2,6-bis(4-hydroxy-3-methoxybenzylidene)cyclohexanone (BHMC), a curcuminoid analogue was synthesised via chemical structure modification by eliminating the unstable β-diketone moiety and converting it into a conjugated double bond while retaining the phenolic hydroxyl group to overcome the limitation by exerting greater cytotoxic, growth suppressive effects with more selective on cancer cells [2]. Therefore, it would be beneficial to look at the cytotoxicity along with the anti-proliferation and -migration effect of BHMC on HepG2 cells.

**Methodology**

The HepG2 and Hs27 cells were treated with various concentrations ranging from 0.781-50μM for 24 and 48hrs via MTT assay. Based on the IC_50_ value obtained, several concentrations were selected to be used in Trypan Blue Exclusion (TBE) assay, Migration assay and Hoechst/Propidium Iodide (PI) staining. Data were analysed using GraphPad Prism software.

**Results and Discussion**

BHMC was observed to be approximately 3-5 times more toxic to HepG2 compared to curcumin with IC_50_ values of 16.73μM and 46.03μM at 24hrs while 4.77μM and 26.00μM at 48 hrs, respectively. However, BHMC was less toxic towards Hs27 cells with the IC_50_ value of more than 30μM at 24 and 48 hrs. BHMC significantly reduced HepG2 by 30-60% at 24hrs and 70-80% after 48hrs at lower concentration than curcumin. In contrast, BHMC only reduced 20-30% of Hs27 at 24hrs and 40-60% at 48hrs. Curcumin reduced 40-60% of HepG2 for 24 and 48 hrs but only reduced 30% of Hs27 cells at similar concentrations and timepoint. Although BHMC and curcumin reduced the percentage of HepG2 cell viability in a concentration- and time-dependent manner, the anti-proliferative effect exerted by BHMC was greater compared to curcumin. BHMC also showed a cytotoxic selective effect on Hs27 especially at 48 hrs with less cell death at lower concentrations compared to HepG2. It is suggested that BHMC was cytotoxic selective towards Hs27 like its parental compound, curcumin. BHMC and curcumin suppress the migration of HepG2 as compared to controls. However, below the IC_50_ value, BHMC exhibits a higher effect in suppressing HepG2 cell migration compared to curcumin. At lower concentrations, characteristics of apoptosis can be observed including cell shrinkage, membrane blebbing, chromatin condensation, DNA fragmentation and apoptotic bodies.

**Conclusion**

BHMC mediates a greater cytotoxic effect in HepG2 by reducing the cell viability, modulating a higher percentage of cell death at lower concentrations compared to curcumin, suppressing migration, and inducing apoptosis compared to Hs27 while cytotoxic selective towards Hs27 cells at similar concentrations.

**References**

[1] Shanmugam, M. K., et al. (2015). The multifaceted role of curcumin in cancer prevention and treatment. In Molecules (Vol. 20, Issue 2, pp. 2728–2769). Multidisciplinary Digital Publishing Institute.

[2] Syed Alwi, S. S. et al. (2019). Cytotoxic Effect of 2,6-bis(4-Hydroxy-3-Methoxybenzylidene) cyclohexanone (BHMC) and Curcumin on Human Liver Cancer Cells, HepG2. Malaysian Journal of Medicine and Health Sciences (MJMHS), 44–50.

## O-25 Unraveling the tumour-regulatory role of miR-3934 in human breast cancer

### Zhi-Xiong Chong^1^, Swee-Keong Yeap^2^, Chee-Mun Fang^1^ and Wan-Yong Ho^1^

#### ^1^Faculty of Science and Engineering, University of Nottingham Malaysia, 43500 Semenyih, Malaysia; ^2^China-ASEAN College of Marine Sciences, Xiamen University Malaysia, 43900 Sepang, Malaysia

##### **Correspondence:** Wan-Yong Ho (WanYong.Ho@nottingham.edu.my)

From The International Conference on Molecular Diagnostics & Biomarker Discovery 2022 (MDBD 2022)

Penang, Malaysia. 11 – 13 October 2022

**Background**

MicroRNAs (miRNAs) are short, single-stranded, and non-coding RNAs that could regulate the expressions of multiple downstream cellular targets post-transcriptionally. By altering the expressions of tumour-promoting and tumour-suppressing targets, miRNAs could act as tumour-modulatory miRNAs in different human cancer. The preliminary findings from our previous work suggested that miR-3934 may be responsible in promoting tumour growth and migration in human breast cancer cell lines [1]. Therefore, this mechanistic study aimed to elucidate the tumour-regulatory role of miR-3934 in human breast cancer cells using *in vitro* cellular assays.

**Methodology**

Triple-negative human breast cancer cell, MDA-MB-231, was stably transfected with miRNA expression plasmids to produce clones that overexpressed miR-3934 mimic, miR-3934 inhibitor, or their respective scrambled control sequences. All transfected and un-transfected cancer cell types were subjected to cell viability, invasion, and wound-healing assays to evaluate the tumour-regulatory role of miR-3934 in breast cancer cells.

**Results and Discussion**

Overexpression of miR-3934 significantly (p<0.05) promoted breast cancer cell proliferation and resistance to chemotherapeutic agent, cisplatin. Besides, miR-3934 also enhanced (p<0.05) the invasion and migration abilities of the MDA-MB-231 cell line as compared to un-transfected control and clones that expressed scrambled control sequences. In contrast to clones that overexpressed miR-3934, clones that overexpressed miR-3934 inhibitor were shown (p<0.05) to have lower proliferation rate, higher sensitivity to killing by cisplatin, and have decreased invasion and migration abilities *in vitro* as compared to all other cell groups. There were no statistically significant differences in the cell proliferation, chemosensitivity to cisplatin, invasion, and migration rates between the untransfected control and clones that expressed the scrambled control sequence. The tumour-promoting role of miR-3934 observed in the current study is consistent with the findings observed in two other miR-3934 studies that involved lung cancer [2] and neuroblastoma [3], in which miR-3934 was reported to downregulate the expression of tumour suppressor protein such as TP53INP1 [2,3]. However, to date, the tumour-modulatory role and downstream target of miR-3934 in breast cancer are still unclear and under-reported. The current study findings suggested that miR-3934 has the potential to be utilised as a prognostic breast cancer biomarker and the introduction of a miR-3934 inhibitor may potentially inhibit breast cancer development. To further confirm the tumour-promoting role of miR-3934, transcriptomics and *in vivo* studies will be conducted to unveil the molecular mechanism of how miR-3934 promotes breast cancer development.

**Conclusion**

Overexpression of miR-3934 was shown to promote the proliferation, chemoresistance, invasion, and migration in the triple-negative MDA-MB-231 cell line while the miR-3934 inhibitor may potentially halt the tumourigenesis process in breast cancer.

**Acknowledgements:** The work was funded by the Fundamental Research Grant Scheme (FRGS/1/2018/STG05/UNIM/02/1 & FRGS/1/2014/SG05/UNIM/02/1) from the Ministry of Higher Education, Malaysia.

**References**

[1] Koh MZ, et al. Regulation of cellular and cancer stem-cell related putative gene expression of parental and CD44+ CD24- sorted MDA-MB-231 cells by cisplatin. Pharmaceuticals. 2021, 14: 391.

[2] Ren A, et al. Downregulation of miR-3934-5p enhances A549 cell sensitivity to cisplatin by targeting TP53INP1. Exp. Ther. Med. 2019, 18: 1653-1660.

[3] Ye W, et al. Downregulation of microRNA-3934-5p induces apoptosis and inhibits the proliferation of neuroblastoma. Exp. Ther. Med. 2019, 18:3729-3736.

## O-27 Selection of T-cell receptor (TCR) like antibody against Human Leukocyte Antigen A-2 (HLA-A2) for cervical cancer diagnostics

### Rehasri Selva rajan, Sylvia Annabel Dass, Tye Gee Jun and Venugopal Balakrishnan

#### Institute for Research in Molecular Medicine (INFORMM), Universiti Sains Malaysia, Penang, Malaysia

##### **Correspondence:** Venugopal Balakrishnan (venugopal@usm.my)

From The International Conference on Molecular Diagnostics & Biomarker Discovery 2022 (MDBD 2022)

Penang, Malaysia. 11 – 13 October 2022

**Background**

Cervical cancer is a leading cause of death among women. Persistent human papillomavirus (HPV) contributes to the carcinogenesis of cervical cancer, in which 70% of all cervical cancer cases are linked to HPV 16 and 18. The available HPV vaccines effectively prevent cervical cancer but do not treat existing cervical cancer. Besides, cervical malignancy detection at early phases remains a challenge. Consequently, treatment begins at the late stages of the disease. Hence, new diagnostics and therapeutics methods are required to treat cervical cancer. In this study, a novel antibody class, known as T cell receptor (TCR)-like antibodies, specific to HPV 16 and 18 E7 oncopeptides, has been hypothesised to enhance cervical cancer diagnostic potentials. The dual functionality of the antibody in mimicking the function of T cell receptor (internal immunosurveillance) and simultaneously executes its antibody effector mechanisms to eliminate a disease effectively opens a new and promising pathway to target cervical cancer.

**Methodology**

Bioinformatics were used to select the target peptides specific to HLA-A2 of HPV 16 and 18 E7. Peptide-MHC (pMHC) complex was formed by refolding HPV peptides with HLA-A2 heavy chain and β2m light chain. Potential TCR-like antibodies were generated by panning the pMHC complexes against an antibody phage display library. The monoclones were analysed with monoclonal ELISA and comparative ELISA before sequence analysis.

**Results and Discussion**

The successful refolding of pMHC was analysed via ELISA with refolded β2m as positive control. A domain antibody library was used to select potential TCR-like antibodies specific to HPV 16 and 18 E7 pMHC by biopanning. Three rounds of biopanning were performed to get highly enriched antibody phages. Antibody phages from round three polyclonal panning were selected for monoclonal analysis because they have highest enrichment. For HPV 16 E7, 20 out of 92 clones were selected for monoclonal analysis and finally 9 clones were sent for sequencing. Meanwhile, for HPV 18 E7, 44 out of 92 clones were selected for monoclonal analysis and finally 17 clones were sent for sequencing. It was difficult to generate a novel group of TCR-like antibody until latest development in technologies like phage display techniques made it possible. The coat protein of a bacteriophage is fused with a protein/peptide and presented on a virion’s surface in phage display technique. Antibody phage display technology has emerged as a famous approach for production of antibodies against various targets including cancer. TCR-like antibody is development based on distinctive roles of T (T cell receptor) and B cells (antibody). Generally, T cell receptor recognises the antigenic peptide presented by MHC complex on all nucleated cell while antibodies generated by B cells can differentiate soluble or membrane bounded antigens which will result in the elimination of illness due to its wider effector functions. Hence, dual functionality of TCR-like antibody in combining both humoral and cell mediated immunity in a single approach makes it an appropriate candidate for cervical cancer diagnosis.

**Conclusion**

Two out of 9 clones and 7 out of 17 clones for HPV 16 E7 and HPV 18 E7, respectively had proper sequence which were analysed by VBASE2 and IMGT/V databases. In the downstream process the proteins of positive antibody clones will be expressed and purified. The purified antibodies will be tested for their binding ability using ELISA and western blot. Finally, the antibodies will be tested in cervical cancer cell lines.

## O-28 Development of T-cell receptor-like antibody against Human Leukocyte Antigen-A11 (HLA-A11) human papillomavirus (HPV) type 16 & 18 oncoprotein E7 for the diagnosis of cervical cancer

### Azrin Syazana Zulcafli^1^, Sylvia Annabel Dass^1^, Tye Gee Jun^1^ and Venugopal Balakrishnan^1^

#### ^1^Institute for Research in Molecular Medicine (INFORMM), Universiti Sains Malaysia, Penang, Malaysia; ^2^Institute, University, City, Country

##### **Correspondence:** Venugopal Balakrishnan (asyazana@student.usm.my / venugopal@usm.my)

From The International Conference on Molecular Diagnostics & Biomarker Discovery 2022 (MDBD 2022)

Penang, Malaysia. 11 – 13 October 2022

**Background**

Cervical cancer is the fourth most common cancer in women worldwide. Human papillomavirus (HPV) is the causative agent of cervical cancers with HPV 16 and 18 being the predominant types causing more than 70% of all cervical cancer cases. Cervical cancer can be prevented through HPV vaccination. However, diagnosing cervical cancer at an early stage remains a challenge. Therefore, a new diagnostic tool is required. In this study, TCR-like antibodies which are a novel class of antibodies that are specific to E7 oncoproteins of HPV 16 and 18 have shown to have the ability to enhance the diagnosis of cervical cancer. The ability of TCR-like antibodies in sandwiching the humoral and cell-mediated immunity in a single approach has brought light to a new promising pathway for targeting cervical cancer.

**Methodology**

Target peptides which are specific to Human Leukocyte Antigen A-11 (HLA-A11) HPV 16 and 18 E7 oncoproteins were identified and selected by bioinformatics. Peptide-MHC (pMHC) complex was formed by refolding the target peptides with heavy chain (HLA-A11) and light chain (β2m). Refolded pMHC were used to develop TCR-like antibodies via biopanning against the antibody phage display library. Positive monoclones obtained were sent for sequencing before being analyzed using VBASE2 and IMGTV databases.

**Results and Discussion**

The successful refolding of pMHC complexes for both HPV 16 E7 and HPV 18 E7 were analysed via ELISA using refolded β2m as the positive control. The refolded pMHC complex was subjected to three rounds of biopanning against antibody phage display library for selection. Phages from the third round of biopanning were selected for both HPV 16 E7 and HPV 18 E7based on the polyclonal ELISA results as they showed the highest enrichment compared to the first and second rounds. 94 single colonies for each HPV 16 E7 and 18 E7 were randomly selected from the phages’ titration for monoclonal biopanning. 10 out of 94 potential monoclones for HPV 16 E7 and 37 out of 94 potential monoclones for HPV 18 E7 were subjected to comparative biopanning based on the monoclonal ELISA results with a value after normalization of ≥0.3. A total of seven out of 10 potential monoclones for HPV 16 and 26 out of 37 potential monoclones for HPV 18 were sent for sequencing based on the monoclonal ELISA results with a value after normalization of ≥0.2. TCR-like antibodies are based on the distinct roles of T cell receptors that are involved in immunosurveillance and direct killing, and the B cell is involved in antibody production. TCR-like antibodies function by differentiating the antigenic peptide which is presented on the MHC molecules and establishing primary defence mechanisms of the antibody. Therefore, the duality of TCR-like antibodies’ function makes it a great candidate for cervical cancer diagnosis.

**Conclusion**

In conclusion, one out of seven clones for HPV 16 and three out of 26 clones for HPV 18, respectively had proper sequences. In the downstream process, the proteins of the positive clones will be expressed and purified. Later, the purified protein will be evaluated for its binding capabilities using Western blot and ELISA. Finally, the antibodies will be evaluated on the cervical cancer cell lines.

## O-29 High throughput molecular profiling of bacterial diversity in Johor mangroves

### Roshan Mascarenhas^1^, Oh Bi Han^1^, Ng Xin Yan^1^, Dominic Kay^1^, Kwa Yee Chu^1^, Pang Kok Lun^1^, James Sy-Keen Woon^1^, Nadine Nograles^1^ and Mahibub Mahamadsa Kanakal^2^

#### ^1^Biomedical Sciences, Newcastle University Medicine Malaysia (NUMed), Johor Bahru, Malaysia; ^2^Faculty of Pharmacy, University College MAIWP International, Kuala Lumpur, Malaysia

##### **Correspondence:** Roshan Mascarenhas (roshan.mascarenhas@newcastle.edu.my)

From The International Conference on Molecular Diagnostics & Biomarker Discovery 2022 (MDBD 2022)

Penang, Malaysia. 11 – 13 October 2022

**Background**

Biomedical research heavily relies on the discoveries of natural compounds and thus it is very important to safeguard nature to build resilience in biomedical research. Mangrove is the coastal ecosystem that has proven to harbour several beneficial microbial communities with a wide range of therapeutic molecules such as antimicrobial agents, anticancer compounds and enzymes. Mangrove provides habitat for several organisms including bacteria that are adapted to the microclimate with high salinity and tidal variation [1]. Some of these bacteria include actinobacteria which form the major source of antibiotics. It is important to identify and discover new antibiotics as there is an increasing threat of resistance to currently available antibiotics and limited discovery of new antibiotics [2]. Mangrove biodiversity in rapidly developing parts of the globe is severely threatened due to the destruction of habitat and thus there is a high threat to the bacterial community. One of the major hurdles in studying mangrove soil bacteria is that it is tedious to culture them and hence difficult to get a complete profile of bacterial diversity by conventional methods. Thus, it is important to adopt high throughput molecular techniques to rapidly identify bacteria to accelerate conservation efforts [3].

**Methodology**

DNA was isolated from mangrove soils sediments from eight mangrove sites around Johor, Malaysia that are grouped based on their vulnerability status. The library was prepared using the multiplexing method with 24 barcodes to distinguish samples and sequenced for the 16S gene using Oxford Nanopore Technologies MinION sequencing. Base-calling of sequencing reads was performed by the Guppy programme and analysed using EPI2ME software. Quantitative PCR was performed to verify linear amplification.

**Results and Discussion**

We have successfully sequenced the bacterial 16S gene in mangrove soil samples from Pulau Kukup, Sungai Pulai, Sungai Melayu, Sungai Danga, Sungai Skudai, Sungai Johor, Sungai Sedili Besar and Sungai Sedili Kechil. We show that the barcoding would enable parallel sequencing of samples from all sites in the same run and amplicons showed linear amplification. We achieved over 6 million high-quality reads per run in less than 24 hours. We report a widespread difference in bacterial diversity between protected mangroves compared to mangroves that have been exposed to anthropogenic activities. This is the first study to show the bacterial community profile of Johor mangroves.

**Conclusion**

MinION sequencing is a quick and efficient way to evaluate bacterial diversity in environmental samples. The findings of this study would enable the development of bacterial community mapping as a repository to help in conservation/rehabilitation as well as monitor the impact of mangrove loss on bacterial diversity.

**Acknowledgement:** The work was funded by the NUMed Internal Research Grant (226900-IG2) and Biomedical Sciences summer research project, NUMed, Malaysia.

**References**

[1] Liu M, *et al.* Microbial community structure of soils in Bamenwan mangrove wetland. Sci. Rep. 2019, 9:1-11.

[2] Azman AS, *et al.* Mangrove rare actinobacteria: taxonomy, natural compound, and discovery of bioactivity. Front. Microbiol. 2015, 6:1-15.

[3] Gong B, *et al.* High-throughput sequencing and analysis of microbial communities in the mangrove swamps along the coast of Beibu Gulf in Guangxi, China. Sci. Rep. 2019, 9:1-10.

## O-30 Ranking of the *Mycobacterium tuberculosis* T-cell epitopes using tabulated immuno-properties for potential Tuberculosis diagnosis and vaccine candidates

### Nurul Syahidah Shaffee^1^, Ezzeddin Kamil Mohamed Hashim^1^, Armando Acosta Dominguez^1^, Maria Elena Sarmiento Garcia San Miguel^1^, Siti Suraiya Md Noor^2^ and Norazmi Mohd Nor^1^

#### ^1^School of Health Sciences, Universiti Sains Malaysia (Health campus), Kelantan, Malaysia; ^2^School of Medical Sciences, Universiti Sains Malaysia (Health campus), Kelantan, Malaysia

##### **Correspondence:** Ezzeddin Kamil Mohamed Hashim (ezzeddin@usm.my, nsyahidahs@student.usm.my)

From The International Conference on Molecular Diagnostics & Biomarker Discovery 2022 (MDBD 2022)

Penang, Malaysia. 11 – 13 October 2022

**Background**

*Mycobacterium tuberculosis* (*Mtb*) is a causative agent for Tuberculosis (TB) with estimated a quarter of the world population has been infected and has the highest cumulative mortality rate among other infectious diseases with 1.3 million death in 2020 alone (WHO, 2021). The report also highlighted TB as the leading cause of mortality by single infectious agent. Ten percent of Latent TB Infection (LTBI) will develop into active infection. Existing diagnostic methods e.g. Tuberculin Skin Test and are not accurate. Newer technique i.e. Interferon Gamma Release Assay is expensive, thus hardly affordable especially in low to middle income countries with high TB incident rate. Currently, there are lack of cheap, sensitive, and specific diagnostic methods to diagnose individuals with LTBI and the current BCG vaccine has limited coverage against adult TB infection.

**Methodology**

Reversed vaccinology technique was applied to shortlist potential *Mtb* T-cell epitopes (TCEs) for diagnosis and vaccine candidates. Six immuno-criteria were selected to rank the TCEs i.e. matching to *Mtb* H37Rv antigens, coverage across 273 *Mtb* strains, association with experiment types of highly expressed *Mtb* genes, promiscuous epitopes in terms of loci and alleles, and population coverage. Each criterion dataset was obtained from a different database and individually pre-processed, analysed using a selected prediction method, and collated using an in- house script. Each factor was equally weighted, and all of the factor results were integrated into a relational table to be used as an interactive scoring platform. At the end, each TCE has six scores from the six factors, and they are combined to produce the final score.

**Results and Discussion**

Looking from the perspective of individual criterion: 1) almost all TCEs have 1-to-1 matching to *Mtb* H37Rv antigens; 2) They also have a high matching coverage across all 273 completed strains tested; 3) Majority of them matched to gene used in human-related experiment literature; 4) Almost all of the TCEs bind to at least a locus, in average a TCE bind to 3.4 (out of 8) loci, and restriction to A-B-C loci has the highest TCE count; 5) In average, each TCE is restricted to 26 (out of 143) alleles; 6) They were tested with their coverage in world, Asian, Southeast Asia and Malaysia populations; where more than half of TCEs cover more than 90% of the world population. Looking from the perspective of top scoring TCEs, top thirty TCEs were further analysed for their associations to cellular functional groups, sequence motifs, common genes used for diagnosis and vaccine and *Mtb* B-cell epitopes. Most of the shortlisted epitopes were found to be parts of potential genes (encoded for proteins) that are widely studied for their cellular and humeral body response i.e. ESAT-6 family (e.g. esxJ, esxK, esxM, esxP, esxW, espJ, and espK), Ag85B, Ag85C, and HSP16.3.

**Conclusion**

These genes have been showed to have potential in TB diagnosis (e.g. Interferon Gamma Release Assay technique) and vaccine candidates.

**Acknowledgement:** This work was supported by the Universiti Sains Malaysia grant numbers 304/PPSK/6315114.

**References**

[1] WHO, Global Tuberculosis report 2020. In *World Health Organization.* (2021). Retrieved from https://www.who.int/publications/i/item/9789240037021

## O-31 Desiccation tolerance mediates ST239-SCC*mec* type III-SCC*mercury* to ST22-SCC*mec* type IV MRSA clonal replacement in hospital settings

### Nurul Amirah binti Mohamad Farook^1^, Raja Mohd Fadhil Raja Abd Rahman^1^, Sharifah Azura Salleh^2^, Muttaqillah Najihan Bin Abdul Samat^2^ and Hui-min Neoh^1^

#### ^1^UKM Medical Molecular Biology Institute (UMBI), Universiti Kebangsaan Malaysia, Kuala Lumpur, Malaysia; ^2^Department of Medical Microbiology and Immunology, Faculty of Medicine, Universiti Kebangsaan Malaysia, Kuala Lumpur, Malaysia

##### **Correspondence:** Hui-min Neoh (hui-min@ppukm.ukm.edu.my)

From The International Conference on Molecular Diagnostics & Biomarker Discovery 2022 (MDBD 2022)

Penang, Malaysia. 11 – 13 October 2022

**Background**

ST239-SCC*mec* type III-SCC*mercury* (ST239-III) to ST22-SCC*mec* type IV (ST22-IV) clonal replacement in methicillin-resistant *Staphylococcus aureus* (MRSA) has been reported globally in many hospitals during the first decade of the new millennium, including in Hospital Canselor Tuanku Muhriz (HCTM), Kuala Lumpur, Malaysia. It was hypothesized that the clonal replacement occurred due to faster growth rate of ST22-IV [1]. To confirm this, we studied and compared the competitive fitness of representative strains from the two clones, ST239-III and ST22-IV isolated from HCTM via broth co-culture and desiccation experiments.

**Methodology**

The strains used in this study were M57/2005 and M222/2009 (both ST239-III), M181/2017 and M080/2017 (both ST22-IV). Chloramphenicol (20ug/ml) susceptibility was used to differentiate the STs of tested strains; ST239 strains were resistant, ST22-IV strains were susceptible. In broth co-culture experiments, pairs of tested strains from different STs were cultured together for 48 hours in brain heart infusion (BHI) broth and platted on drug-free and chloramphenicol-supplemented BHI agar. In desiccation experiments, pairs of tested strains from different STs were exposed to desiccation for 48 hours on petri plates prior rehydration of the cultures with normal saline, followed by drug-free and chloramphenicol-supplemented BHI agar platting and incubation at 37^o^C overnight. Numbers of colonies on agar plates for both STs in both experiments were enumerated. SCC*mec* typing of representative colonies was also performed for ST confirmation.

**Results and Discussion**

For broth co-culture experiments, mean of 194.5, (135-272) ST239-III colonies and 31.5, (4-58) ST22-IV colonies were observed at 10^-6^ serial dilution. On the other hand, ST22-IV produced a higher number of surviving colonies on desiccation assays (29, 5-58) compared to ST239-III (2.5, 0-5) at 10^-6^ serial dilution. While ST239-III outcompeted ST22-IV in all tested pairs for the broth co-culture studies, the latter clone had better tolerance to desiccation. As MRSA is a nosocomial pathogen with clonal dissemination in hospitals [2], higher desiccation tolerance could have provided ST22-IV an edge to survive and disseminate, ultimately replacing ST239-III in the hospital environment.

**Conclusion**

ST22-IV was found to have higher desiccation tolerance compared to ST239-III. This edge in bacterial fitness might be the cause of ST239-III to ST22-IV clonal replacement in nosocomial MRSAs; further studies will be required to confirm this.

**Acknowledgment:** This research was funded by research university grant GUP-2020-079 from Universiti Kebangsaan Malaysia.

**References**

[1] Knight, et al. Shift in dominant hospital-associated methicillin-resistant *Staphylococcus aureus* (HA-MRSA) clones over time. *J Antimicrob Chemother.* 2012; 67(10), 2514–2522.

[2] Tan XE et al. Clonal distribution and possible microevolution of methicillin-resistant *Staphylococcus aureus* strains in a teaching hospital in Malaysia, *Asian Pac J Trop Biomed*. 2013; 3(3):224-8.

## O-33 Ligand-based pharmacophore modeling, molecular docking and molecular dynamics study targeting prolyl oligopeptidase enzyme for effective treatment for Parkinson’s disease: Computational approach

### Yahaya Sani Najib^1, 2^, Yusuf Oloruntoyin Ayipo^1, 3^, Waleed Abdullah Ahmad Alananzeh^1^ and Mohd Nizam Mordi^1^

#### ^1^Centre for Drug Research, Universiti Sains Malaysia, USM 11800, Pulau Pinang, Malaysia; ^2^Department of Pharmaceutical and Medicinal Chemistry, Bayero University Kano, PMB 3011, Kano Nigeria; ^3^ Department of Chemistry, Kwara State University, Malete, PMB 1530, Ilorin, Nigeria

##### **Correspondence:** Yahaya Sani Najib (najibsani62@gmail.com)

From The International Conference on Molecular Diagnostics & Biomarker Discovery 2022 (MDBD 2022)

Penang, Malaysia. 11 – 13 October 2022

Prolyl oligopeptidase (POP) are serine protease enzymes implicated in the pathogenesis of Parkinson’s disease (PD) through the increased aggregation of α-synuclein protein in the brain. The current treatment options for PD are only symptom-targeted, while an effective therapeutic strategy remains a challenge. The study aimed to identify potent anti-PD drugs with inhibitory potentials against POP using ligand-based receptor modelling, Glide XP docking, molecular dynamics and pk-CSM pharmacokinetics ADMETprediction parameters. Results indicated that the ligand-based (LB) model generated pharmacophore with 6 features, having 1 hydrophobic, 1 positive ionizable group, 2 aromatic rings, and 2 hydrogen bond acceptors. A total of 23 hits with a Gunner-Henry (GH) score of 0.7 and enrichment factor (EF) of 30.24 were obtained as validation protocols, making it an ideal model. The LB model retrieved 177 hit compounds from 69,543 natural Interbioscreen databases virtually. Interestingly, ligands 1, 2, 3, 4 and 5 demonstrated higher binding affinity to the enzyme with Glide XP docking of -9.004, -8.818, -8.749, -8.708, -8.707 kcal/mol than GSK552 and ZPP with -8.187, and -6.835 respectively. Similarly, their binding free energies were -48.424, -51.879, -51.684, -45.191, and -49.567 kcal/mol respectively. Molecular dynamics indicated that the ligands 1, 2, & 4 demonstrated better stability than GSK552. Pharmacokinetic profiles of the ligands indicated their druggability and low toxicity profile. The ligands are recommended as adjuvant /single candidates as ant-PD candidates for translational study.

## O-34 Inhibitory effects of andrographolide in PC-3 cell line and the induction of apoptosis via the involvement of caspases activity

### Janany Manimaran and Daruliza Kernain Mohd Azman

#### Institute for Research in Molecular Medicine (INFORMM), Universiti Sains Malaysia, Penang, Malaysia

##### **Correspondence:** Daruliza Kernain Mohd Azman (daruliza@usm.my)

From The International Conference on Molecular Diagnostics & Biomarker Discovery 2022 (MDBD 2022)

Penang, Malaysia. 11 – 13 October 2022

**Background**

Andrographolide is a labdane diterpenoid isolated from the plant *Andrographis paniculata*. This substance has numerous medicinal uses, notably anticancer effects. A previous study has revealed that andrographolide inhibits the growth of lung, brain, colon, and breast cancer cells [1]. Due to a lack of research, however, it is thought that the knowledge of andrographolide’s anti-cancer effects on prostate cancer cells is relatively poor. In the current study, andrographolide was assessed on PC-3 cells, an aggressive androgen-independent prostate cancer cell line.

**Methodology**

Cytotoxicity analysis is vital in drug discovery research for assessing the biocompatibility of the drug being used on cancer cells. This work used the WST-1 assay to determine the cell survival of PC-3 cancer cells and Hs27 normal cells exposed to varying doses of andrographolide (0-200 μM). Metastasis is essential for the disease to progress; hence, the scratch assay and the transwell invasion assay were used to test andrographolide on PC-3 cells. In order to study the cell death mechanism after the PC-3 cells are treated with andrographolide, DNA damage and apoptosis assay have been done. Finally, the caspase-mediated pathway is analysed by carrying out caspase 3, 8 and 9 assays upon the treatment of andrographolide.

**Results and Discussion**

The results indicate that andrographolide dose-dependently suppresses the viability of PC-3 cells but not Hs27 cells. The NCI considers the LC 50 value of 26.42 μM (after 48 hours of incubation) acceptable. In this study, three different concentrations of andrographolide were used: control, half LC 50, and LC 50 (0, 13.21, and 26.42 μM) in all subsequent analyses. The results showed that andrographolide inhibits both migration and invasion compared to the control. The presence of a comet tail has revealed that a 26.42 μM andrographolide treatment produces the maximum DNA damage to single cells, followed by 13.21 μM and 0 M. In addition, the maximum cell death activity was seen at 26.42 M andrographolide concentration, followed by 13.21 μM and the control. The activity of caspases 3 (executor caspase), 8 (intrinsic pathway), and 9 (extrinsic pathway) in mediating apoptosis increased significantly. The half LC 50 (13.21 μM) demonstrates significantly more activity in these caspases than the LC 50 (26.21 μM), in accordance with the new finding related to the caspase storm scenario [2]. This enables us to identify 13.21 μM as the andrographolide dosage that approaches the ideal range for caspase activity activation.

**Conclusion**

The ability of andrographolide to prevent the progression of cancer in PC-3 cancer cells has been proven through the regulation of caspase-mediated apoptosis, suppression of metastasis, and induction of DNA damage.

**References**

[1] Mishra, S. K., Tripathi, S., Shukla, A., Oh, S. H., & Kim, H. M. (2015). Andrographolide and analogues in cancer prevention. *Frontiers in Bioscience-Elite*, *7*(2), 293-304.

[2] Tao, Z., Goodisman, J., Penefsky, H. S., & Souid, A. K. (2007). Caspase activation by anticancer drugs: the caspase storm. Molecular Pharmaceutics, 4(4), 583-595.

## O-35 Lentiviral modification of hard-to-transduce NK-92MI cells

### Chin Ding Sheng and Tye Gee Jun

#### Institute for Research in Molecular Medicine (INFORMM), Universiti Sains Malaysia, Penang, Malaysia

##### **Correspondence:** Tye Gee Jun (geejun@usm.my)

From The International Conference on Molecular Diagnostics & Biomarker Discovery 2022 (MDBD 2022)

Penang, Malaysia. 11 – 13 October 2022

**Background**

Natural killer (NK) cells are lymphocytes that play an important role in both our innate and humoral immunities. NK cell functions are dictated by the myriad of receptors, regulating both activating and inhibitory signals. Studies found that through interactions with the CD16 receptor alone, NK cells can be activated for antibody-dependent cellular cytotoxicity (ADCC) to occur. The ADCC mechanism can be used to home NK cell killing towards designated targets through detection by target-specific antibodies. Unfortunately, NK cell studies are hampered by the tedious sourcing of primary NK cells. NK cell lines such as NK-92 and NK-92MI are now available but they lack certain features compared to primary cells. Furthermore, NK cells are less susceptible to genetic modifications such as electroporation and viral-based methods. Recently, lentiviral vectors were successfully used in the clinical setting for cell modifications. In this study, we used a third-generation lentiviral vector, termed RRL-lentivirus, to modify the NK-92MI cells for surface expression of CD16 receptors and possibly restore their ability for ADCC.

**Methodology**

High binding affinity 158V-CD16 and GFP gene sequences were molecularly cloned into RRL-lentiviral transfer vector. The transfer vector plasmid containing the gene of interest and three types of helper plasmids were simultaneously transfected into HEK-293T packaging cells by calcium phosphate transfection. After 48 hours, the supernatant containing the lentiviral particles was collected and filtered through a 0.45um CA filter. This was then concentrated using PEG-6000 precipitation method and stored in serum-free media at -80°C. Using K-562 cells as positive controls, NK-92MI cells were transduced using GFP-RRL lentivirus or CD16-RRL lentivirus. Transduction replicates were done with or without the addition of polybrene polycation. The expression of reporter GFP was monitored using fluorescence microscopy, and the levels of transgene expressions were further validated through flow cytometry using FITC-conjugated anti-human CD16 antibodies.

**Results and Discussion**

After GFP-lentivirus transduction was performed, the control K-562 samples showed GFP fluorescence on day 3, while the NK-92MI samples displayed GFP on day 5. The difference between the samples transduced with and without polybrene was compared through flow cytometry. At MOI of 1, GFP-transduced K-562 cells resulted in a GFP-positive population of 95.6% without polybrene and this improved to 99.5% when polybrene was added. At the same MOI, CD16-transduced K-562 did not result in the formation of two distinct CD16 populations. However, all transduced samples showed a significant positive shift in the detected FITC compared to negative controls. At the MOI of 10, GFP-transduced NK-92MI cells resulted in GFP-positive population of 3.5% without polybrene, and the population increased to 7.2% with polybrene added. CD16-transduced NK-92MI cells resulted in a CD16-positive population at 1.0%, while the addition of polybrene increased this expression to 3.1%.

**Conclusion**

This study demonstrated the feasibility of using a third generation RRL-lentiviral vector for NK-92MI cell transduction, but further optimisations will be required before *in vitro* assays such as natural cytotoxicity and ADCC assays can be performed using these modified cells.

**References**

[1] Chan, L., Hardwick, N., Darling, D., Galea-Lauri, J., Gäken, J., Devereux, S., ... & Farzaneh, F. (2005). IL-2/B7. 1 (CD80) fusagene transduction of AML blasts by a self-inactivating lentiviral vector stimulates T cell responses in vitro: a strategy to generate whole cell vaccines for AML. Molecular Therapy, 11(1), 120-131.

[2] Jochems, C., Hodge, J. W., Fantini, M., Fujii, R., Y Morillon Maurice, I. I., Greiner, J. W., ... & Schlom, J. (2016). An NK cell line (haNK) expressing high levels of granzyme and engineered to express the high affinity CD16 allele. Oncotarget, 7(52), 86359.

[3] Nordin, F., Hamid, Z. A., Chan, L., Farzaneh, F., & Hamid, M. K. (2016). Transient expression of green fluorescent protein in integrase-defective lentiviral vector-transduced 293T cell line. In Lentiviral Vectors and Exosomes as Gene and Protein Delivery Tools (pp. 159-173).

## O-37 Immunoinformatics analysis on human coronavirus spike protein for universal immunogen discovery

### Chin Peng Lim^1,2^, Boon Hui Kok^1^, Hui Ting Lim^1^, Chiuan Yee Leow^2^ and Chiuan Herng Leow^1^

#### ^1^Institute for Research in Molecular Medicine (INFORMM), Universiti Sains Malaysia, Penang, Malaysia; ^2^School of Pharmaceutical Sciences, Universiti Sains Malaysia, 11800 Gelugor, Penang, Malaysia

##### **Correspondence:** Chiuan Herng Leow (herng.leow@usm.my)

From The International Conference on Molecular Diagnostics & Biomarker Discovery 2022 (MDBD 2022)

Penang, Malaysia. 11 – 13 October 2022

**Background**

Coronaviruses are well-known to possess high mutation rate. The recent pandemic has demonstrated this fact that multiple SARS-CoV-2 variants have emerged since the first occurrence in 2019. Variants such as Beta, Delta and Omicron variant, even showed the ability of immune evasion in convalescent and vaccinated individuals [1], raising global concern about the efficacies of existing vaccines. Immunoinformatics approach is gaining traction in vaccine development due to significant time and cost reduction in immunogenicity studies and improved reliability [2]. Viral genome can be analysed for the mapping of potential T-cell and B-cell epitopes. Structural proteins, particularly spike protein (S) have been studied extensively as promising vaccine candidates. One rationale is that the RBD of SARS-CoV-2 attaches to the ACE2 receptor on the host cells to initiate infection. Taken together, a vaccine that offers protection over a wide spectrum of coronaviruses is crucially demanded.

**Methodology**

The sequences of S proteins of the SARS-CoV-2 variants as well as SARS-CoV and MERS-CoV were retrieved from the NCBI database. Multiple sequence alignment (MSA) was performed to identify the conserved regions among S proteins from different human coronaviruses. All conserved regions were analysed for the antigenicity through VaxiJen. The conserved regions that passed the threshold value of 0.5 were then analysed for T-cell and B-cell epitope predictions using NetMHCpan EL4.1, IEDB recommended 2.22 and BepiPred 2.0, respectively. The antigenic conserved regions were also used to construct 3D model using SWISS-MODEL followed by structural refinement using GalaxyRefine2. All refined models were validated using ERRAT and PROCHECK. Lastly, the docking of 3D models against TLR-3 was performed via PatchDock and FireDock.

**Results and Discussion**

Based on the MSA result, S protein contains 12 conserved regions, which were then subjected to further analyses. Altogether 9 conserved regions were above the antigenicity threshold value and therefore selected. In terms of epitope prediction, the antigenic conserved regions were predicted to contain a total of 69 MHC Class-I epitopes, 45 MHC Class-II epitopes and 5 linear B-cell epitopes. Furthermore, all antigenic conserved regions were sent for 3D model building followed by structural refinement. The best refined model for each conserved region was chosen based on the Galaxy energy. These refined models were validated and the results showed that the models were of good quality. Following that, the refined model for each conserved region was docked against TLR-3. The global energies of complexes formed between the conserved regions and TLR-3 fell within the range of -11.74 to -7.36 kcal/mol. This implies that the conserved regions have good binding affinities with TLR-3.

**Conclusion**

The identified conserved regions of S protein were predicted to have a significant number of epitopes and showed promising docking results. This study provides some insights about the interaction of conserved S peptides with TLR-3, contributing to the vaccine design. Still, further analyses such as molecular dynamics and immune simulation are required to polish the results. *In vitro* and *in vivo* validation are also essential to evaluate the immunological roles of designed universal coronavirus vaccine.

**Acknowledgement:** We would like to thank the funding sponsored by Ministry of Higher Education for Fundamental Research Grant Scheme (FRGS) with Project Code: FRGS/1/2022/SKK0/USM/02/5, and FRGS/1/2021/SKK06/USM/02/12 to complete this work.

**References**

[1] X. Zhang et al., “SARS-CoV-2 Omicron strain exhibits potent capabilities for immune evasion and viral entrance,” *Signal Transduct. Target*. *Ther*. 2021 61, vol. 6, no. 1, pp. 1–3, Dec. 2021, doi: 10.1038/s41392-021-00852-5.

[2] E. Raoufi et al., “Epitope prediction by novel immunoinformatics approach: A state-of-the-art review,” *Int J Pept Res Ther*, vol. 26, no. 2, pp. 1155–1163, 2020, doi: 10.1007/s10989-019-09918-z.

## O-38 Andrographolide induced apoptosis by enhancing c-Myc/p53 in human glioblastoma DBTRG-05MG cell line

### Nurul Syamimi Othman and Daruliza Kernain Mohd Azman

#### Institute for Research in Molecular Medicine (INFORMM), Universiti Sains Malaysia, Penang, Malaysia

##### **Correspondence:** Daruliza Kernain Mohd Azman (daruliza@usm.my)

From The International Conference on Molecular Diagnostics & Biomarker Discovery 2022 (MDBD 2022)

Penang, Malaysia. 11 – 13 October 2022

**Background**

Human glioblastoma multiforme (GBM) is one of the most malignant brain tumors. The conventional GBM treatment faces a problem due to the presence of the blood-brain barrier (BBB) and the post-treatment effect. Then, andrographolide has attracted many researchers because it can cross the BBB and easily distribute it into different brain regions [1]. Next, targeted therapy also is one of the treatments that can reduce the post-treatment effect because it can specifically target cancer cells without affecting normal cells. Recently, the small molecule targeted therapy by exploring on the molecular targets of oncogenes or tumor suppressor genes such as c-Myc and p53 in regulating the apoptosis signaling pathway still remains debatable [2]. Therefore, this study aimed to elucidate the mechanism action of andrographolide towards c-Myc/p53 induced apoptosis in DBTRG-05MG cell line.

**Methodology**

The cytotoxic effect of andrographolide was assessed by WST-1 assay in DBTRG-05MG and SVGp12 cell lines. Both cell lines were treated for 24, 48, and 72 hours with varying concentrations of andrographolide (0.781 – 200 μM). The WST-1 reagent was added to each well. Then, plates were incubated, and the absorbance (Abs.) was measured with an ELISA reader (Multiscan Spectrum) at wavelength 450 nm and the reference reading of 630 nm. The LC_50_ values were computed and will be further used as active doses and time in the subsequent experiments. Next, the apoptosis assay was assessed using annexin V-FITC / PI double staining. Cells pellet of DBTRG-05MG were collected after treatment with andrographolide and resuspended with V-FITC, and PI staining. The cells were incubated in the dark at room temperature for 30 minutes before analyzing the stained cells using a BD FACS CantoTM Flow Cytometer. The gene and protein expression level c-Myc and p53 signaling pathway were then assessed using qRT-PCR and western blot. The protein-protein interaction between c-Myc and p53 was determined by a reciprocal experiment of the co-immunoprecipitation (co-IP) using DBTRG-05MG total cell lysate.

**Results and Discussion**

Andrographolide has significantly reduced the viability of DBTRG-05MG cell lines in a concentration- and time-dependent manner. The recorded LC_50_ values for DBTRG-05MG cell lines for 24, 48 and 72 hours of treatment were 42.82 μM, 27.21 μM and 13.93 μM, respectively. While in the non-cancerous cell line, SVGp12 cannot be determined because the percentage cell viability for the normal cell remained ~80% after being treated with the highest concentration (200 μM) of andrographolide for 24h results. Andrographolide also induced apoptosis in the DBTRG-05MG cell line, by inducing c-Myc and p53 expression at the gene and protein level. The percentage of apoptotic cells in control cells was 0.01%, and after the cell lines were exposed to 13.95 μM and 27.9 μM of andrographolide for 72 hours, the percentage of the apoptotic cell increased to 5.2% and 16.5%, respectively. Western blot results demonstrated that c-Myc overexpression also increased the production of the anti-apoptotic protein p53. Our findings revealed that c-Myc and p53 positively interact in triggering the apoptotic signaling pathway when the lane immunoprecipitated (IP) result for p53 and probed with anti-c-Myc in western blot analysis, which showed the presence of the band for c-Myc protein (62kDA).

**Conclusion**

This study successfully discovered the involvement of c-Myc and p53 in the inhibition of DBTRG-05MG cell line via apoptosis following andrographolide treatment. However, further study needs to be extended to evaluate the cytotoxicity of andrographolide *in vivo* and *in vitro*. The function of other regulatory genes that may interact with the c-Myc signaling pathway must also be investigated intensively.

**Acknowledgement:** This research was supported by the Ministry of Education (MOE) under the Fundamental Research Grant Scheme (FRGS) 203/CIPPM/6711721.

**References**

[1] J. Lu, et al. A review for the neuroprotective effects of andrographolide in the central nervous system, Biomed. Pharmacother. 2019, 117.

[2] M. Ashrafizadeh, et al. C-Myc Signaling Pathway in Treatment and Prevention of Brain Tumors. Curr. Cancer Drug Targets. 2020, 21:2–20.

## O-39 *In vitro* uptake and activation of human dendritic cells by liposomes derived from total lipid of *Mycobacterium smegmatis*

### Nurawo Azlyna Ahmad Suhaimi^1^, Siti Suraiya Md Noor^2^, Rohimah Mohamud^1^, Nor Asyikin Nordin^1^ and Ramlah Kadir^1^

#### ^1^Department of Immunology, School of Medical Sciences, Universiti Sains Malaysia, 16150 Kubang Kerian, Kelantan, Malaysia; ^2^Department of Medical Microbiology and Parasitology, School of Medical Sciences, Universiti Sains Malaysia, Kota Bharu, Malaysia

##### **Correspondence:** Ramlah Kadir (ramlahkadir@usm.my)

From The International Conference on Molecular Diagnostics & Biomarker Discovery 2022 (MDBD 2022)

Penang, Malaysia. 11 – 13 October 2022

**Background**

Liposomes are self-assembled lipids that have attracted scientific attention as efficient drug delivery vehicles and adjuvants. They possess a unique vesicular structure and can be derived from natural and synthetic substances. Liposomes can mimic the biological membrane of drugs, thus extending the half-life and minimizing the toxicity levels while delivering them to the target organs. The adjuvant mechanism of liposomes has been intimately associated with the stimulation of desired immune responses upon the exposure of antigen and immune cell targeting. This current study mainly targets to investigate the uptake and activation of human dendritic cells (DCs) by liposomes derived from the total lipid of *Mycobacterium smegmatis.*

**Methodology**

Liposomes derived from *Mycobacterium smegmatis* were synthesized and characterized using Ziehl-Neelsen (ZN stain) and field emission scanning electron microscopy (FESEM). Fresh whole blood has been collected from three distinctive groups: TST-negative individuals, TST-positive individuals, and active pulmonary TB patients. Peripheral blood mononuclear cells (PBMCs) were separated by Lymphoprep density centrifugation and cultured with lipopolysaccharide (LPS) and liposomes. The activation of DCs by *M. smegmatis* liposomes has been analyzed through the expression level of DCs surface markers (HLA-DR, CD11c, CD123, CD86) in flow cytometry and the secretion of cytokines (IL-4, IL12p70, and IFN-γ) via ELISA.

**Results and Discussion**

The characterization of liposomes under FESEM demonstrated a size ranging from 20 nm^-135^ nm with spherical structures. The results showed levels of HLA-DR and CD86 expression reduced in the group of DCs of active pulmonary TB with liposomes compared to the group of TST-negative and TST-positive individuals. However, the interaction of DCs from active pulmonary TB patients with liposomes exhibited an increased level of HLA-DR with low expression of CD86 compared to other groups. IL-12p70 was a highly significant increase in active pulmonary TB group compared to the group of TST-negative and TST-positive individuals. The group of active pulmonary TB patients also showed a significant increase in IL-4 and IFN-γ than the group of TST-positive individuals. Meanwhile, the secretion of all cytokines was the lowest in the group of TST-positive individuals compared to the other groups. These events emphasized the malfunction of DCs in a TB condition and highlighted the capability of liposomes to support the antigen presentation by human DCs in TB infection.

**Conclusion**

Liposomes synthesized from *Mycobacterium smegmatis* had been successfully produced and classified as small unilamellar vesicles (SUVs). The exposure of DCs with liposomes particularly improved the antigen presentation activity with low activation of DCs in active pulmonary TB patients compared to the other study cohort. The results from this current study supported liposomes as potent vaccines and adjuvants for immunotherapy.

**Acknowledgement:** This work was funded by the Fundamental Research Grant Scheme (FRGS) (FRGS/1/2018/SKK08/USM/03/1) from the Ministry of Higher Education, Malaysia.

## O-40 Identification of medicinal fungi by molecular analysis

### Eng Wei Keat^1^, Leow Chiuan Yee^2^, Lai Ngit Shin^1^, Sasidharan Sreenivasan^1^ and Leow Chiuan Herng^1^

#### ^1^Institute for Research in Molecular Medicine (INFORMM), Universiti Sains Malaysia, Penang, Malaysia; ^2^School of Pharmaceutical Sciences, Universiti Sains Malaysia, Penang, Malaysia

##### **Correspondence:** Leow Chiuan Herng (herng.leow@usm.my)

From The International Conference on Molecular Diagnostics & Biomarker Discovery 2022 (MDBD 2022)

Penang, Malaysia. 11 – 13 October 2022

**Background**

Mushrooms or higher fungi are heterotrophic eukaryotic organisms on Earth. Fungi are also having its great medical and environmental importance. Hence, the precise and rapid identification of fungi species from specimens is important for the field applications, such as, sustainable use and conservation of biodiversity, prevention and control of fungal pathogens, human health and ecological monitoring [1]. DNA barcoding is a great tool for specimen identification that can be used for identifying and recognizing various types of fungi. The internal transcribed spacer (ITS) regions of the nuclear ribosomal RNA gene cluster were utilized as a universal DNA barcode marker for fungi specimens which was suggested by Schoch and his team [2]. In this study, we identified 11 randomized fungi specimens by using ITS primers.

**Methodology**

The fungi specimen grown on Petri plates containing PDA medium was obtained from the lab. A small number of mycelia of fungi specimens was picked using sterile forceps from the fully-grown culture and was transferred into a 1.5 mL microcentrifuge tube consisting of 600 μL of sterile distilled water. The mycelia were homogenized by using pipette tips. The mixture was vortexed thoroughly and centrifuged at 10,000 x g for 1 minute. The supernatant was discarded and continued by using the EasyPure Plant Genomic DNA kit according to the manufacturer’s instructions to extract fungi DNA. MyTaq HS Red Mix, 2x, universal ITS primer pairs, extracted fungi DNA sample template and sterile distilled water were used for performing PCR amplification using Agilent SureCycler 8800 with 35 cycles of amplification. The PCR products were undergone electrophoresis to separate DNA. The gel was observed and captured using a Syngene Bioimaging system. The EasyPure PCR purification kit was used to purify PCR products to remove primers and nucleotides according to the manufacturer’s instructions. The purified PCR products were sent for sequencing. The FinchTV software, BLASTn algorithm at the NCBI website and DNA Bold program were used to analyze the sequences data.

**Results and Discussion**

Eleven fungi specimens were determined by its size by using the Syngene Bioimaging System. The highest base pair of fungi specimens was Sample 10, which had 850 base pairs. The lowest base pair of fungi samples were Samples 1, 4, 5 & 6, which had 600 base pairs. The range of base pair of fungi specimens was from 600 bp to 850 bp. Therefore, 11 fungi specimens were identified by using the FinchTV software, BLASTn algorithm at the NCBI website and DNA Bold program through analyzing their sequences data. The outcomes of the analysis revealed these specimens were *Hypoxylon sp. LA01*, *Lignosus rhinocerotis*, *Ganoderma lucidum*, *Cordyceps militaris*, *Hemistropharia albocrenulata*, *Inonotus obliquus*, and *Hericum erinaceus*.

**Conclusion**

The DNA extracted from randomized fungi specimens were identified successfully using molecular analysis. The works presented here reveal this method will potentially aid in the authentication of medicinal fungi from unknown specimens.

**Acknowledgement:** We would like to thank funding sponsored by Ministry of Higher Education for Fundamental Research Grant Scheme (FRGS) with Project Code.: FRGS/1/2021/SKK06/USM/02/12, and FRGS/1/2022/SKK0/USM/02/5 to complete this manuscript.

**References**

[1] Xu, J. (2017). Fungal DNA barcoding. *The 6th International Barcode of Life Conference*, *01*(01), 913–932.

[2] Schoch, et al.(2012). Nuclear ribosomal internal transcribed spacer (ITS) region as a universal DNA barcode marker for Fungi. *Proceedings of the National Academy of Sciences of the United States of America*, *109*(16), 6241–6246.

## O-41 Effect of andrographolide on proliferation, migration, and invasion of MDA-MB-231 and MCF-7 cell lines

### Deeza Syafiqah Mohd Sidek and Daruliza Kernain Mohd Azman

#### Institute for Research in Molecular Medicine (INFORMM), Universiti Sains Malaysia, Penang, Malaysia

##### **Correspondence:** Daruliza Kernain Mohd Azman (daruliza@usm.my)

From The International Conference on Molecular Diagnostics & Biomarker Discovery 2022 (MDBD 2022)

Penang, Malaysia. 11 – 13 October 2022

**Background**

Breast cancer is the commonly diagnosed and one of the leading causes of death among women worldwide [1]. Current standard treatment includes chemotherapy, surgery, and radiotherapy. Some patient discontinued their treatment due to the side effects of the treatments which leads to relapse. Therefore, alternative medicines from natural plants with fewer side effects now widely studied. Andrographolide is a diterpene lactone derived from *Andrographis Paniculata.* Andrographolide had previously demonstrated to have anti-inflammatory, anti-cancer, anti-fungal, and anti-viral properties. This study is done to determine anti-cancer effects of andrographolide in breast cancer cells by evaluating the cell viability, migration, and invasion in two different cancer cell lines which are MDA-MB-231 (a triple negative cell line) and MCF-7 (ER positive cell line).

**Methodology**

MDA-MB-231 and MCF-7 cells were treated with a different concentration of andrographolide (0.781μM - 200 μM) for 24, 48 and 72 hours. The cell viability was observed using the WST-1 reagent. The LC_50_ and ½ LC_50_ values obtained from WST-1 were then used for scratch and transwell invasion assays to determine the rate of migration and invasion for both cell lines. The cells were both treated with LC_50_ and ½ LC_50_ concentration of andrographolide for a different timeline, MDA-MB-231 was treated for 72H and MCF-7 was treated for 48H before proceeding to scratch and invasion assay.

**Results and Discussion**

Andrographolide had shown a significant effect on MDA-MB-231 and MCF-7 cells by suppressing the cell viability in a concentration dependent manner. Our findings are consistent with the previous study that showed andrographolide reduced the cell viability in cancer cell but have a minimum effect on normal cells in a concentration dependent manner. Andrographolide inhibit the migration of MDA-MB-231 and MCF-7 cell lines at 72 and 48 hours, respectively. The wound gap area increased in both cells treated with andrographolide at two different concentrations compared to both untreated cells. Invasion assay showed that andrographolide inhibit the invasion of MDA-MB-231 and MCF-7 cell lines at a low concentration which aligned with the previous research that showed andrographolide inhibit the invasion of MDA-MB-231 [2].

**Conclusion**

Andrographolide showed the migration and invasion inhibition properties in a concentration dependent manner in MDA-MB-231 and MCF-7 cells. Therefore, andrographolide is shown to have a promising future to be develop as a potential treatment for breast cancer. Further research needs to be done to elucidate the mechanism of cell death and whether it involved in either cell cycle or apoptosis pathway.

**Acknowledgement:** This work was funded by the Ministry of Education (MOE) under the Fundamental Research Grant Scheme (FRGS) ,203/CIPPM/6711721.

**References**

[1] Sung, H, Ferlay, J, Siegel, RL, Laversanne, M, Soerjomataram, I, Jemal, A, Bray, F. Global cancer statistics 2020: GLOBOCAN estimates of incidence and mortality worldwide for 36 cancers in 185 countries. CA Cancer J Clin. 2021, 71, 209- 249.

[2] Zhai, Z.; Qu, X.; Li, H.; Ouyang, Z.; Yan, W.; Liu, G.; Liu, X.; Fan, Q.; Tang, T.; Dai, K.; et al. Inhibition of MDA-MB-231 breast cancer cell migration and invasion activity by andrographolide via suppression of nuclear factor-kappaB-dependent matrix metalloproteinase-9 expression. Mol. Med. Rep. 2015, 11, 1139–1145.

## O-42 Culture and biochemical testing versus 16S rRNA next- generation sequencing for bacterial identification from clinical samples: Practicability, cost and turn-around time in a Malaysian laboratory

### Nurnabila Syafiqah Muhamad Rizal^1^, Hui-min Neoh^1^, Ramliza Ramli^1^, Petrick @ Ramesh A/L K Periyasamy^1^, Alfizah Hanafiah^1^, Muttaqillah Najihan Abdul Samat^1^, Toh Leong Tan^1^, Kon Ken Wong^1^, Sheila Nathan^1^, Sylvia Chieng^1^, Seow Hoon Saw^2^ and Bee Yin Khor^3^

#### ^1^Universiti Kebangsaan Malaysia, Bangi, Malaysia; ^2^Universiti Tunku Abdul Rahman, Perak, Malaysia; ^3^BioEasy Sdn Bhd, Kuala Lumpur, Malaysia

##### **Correspondence:** Hui-min Neoh (hui-min@ppukm.com.my)

From The International Conference on Molecular Diagnostics & Biomarker Discovery 2022 (MDBD 2022)

Penang, Malaysia. 11 – 13 October 2022

**Background**

Bacterial pathogens are mostly identified via culture and biochemical testing (CBtest) in Malaysian clinical microbiology laboratories. In this study, we assessed the practicability, cost and turn-around-time (TAT) of utilising 16S rRNA next- generation sequencing (16SNGS) versus CBtest for bacteria identification from clinical samples collected at the Department of Diagnostic Laboratory Services, UKM Medical Centre, Kuala Lumpur.

**Methodology**

Twenty-four clinical samples (eight each from pus, respiratory and urine specimens) were included into the study. CBtest was carried out via microscopy and staining the samples, bacterial culture and isolation followed by biochemical tests for species identification. 16SNGS was performed via amplification of V3- V4 regions of 16S rRNA gene from total DNA extracted from each sample and sequenced using the MiSeq system; raw sequencing data was analyzed using the B.E Patho software. TAT was calculated from the initiation of sample processing to test report generation. Cost analysis included cost for reagents and disposable procurement as well as hands-on labour charges.

**Results and Discussion**

Via CBtest, 18 samples were reported as positive for single- bacterial infection (n = 5, 20.8%), mixed growth (n = 8, 33.3%) and normal flora (n = 5, 20.8%), respectively; remaining samples were culture-negative. 16SNGS detected the relative abundance of various bacteria genera in all tested samples. TAT of 16SNGS workflow (six days) was shorter compared to CBtest for identification of slow-growing, fastidious bacteria (72 days). Utilisation of the B.E. Patho “plug-and-play” bioinformatics suite allowed 16SNGS analysis to be performed by personnel without bioinformatics training. Nonetheless, identification cost per sample via 16SNGS (RM 1704.70) was very much higher than CBtest (RM 2.80 – RM 42.90) for all samples.

**Conclusion**

16SNGS allowed identification of various bacterial genera in clinical samples with a faster TAT for slow-growing and fastidious bacteria. Lowering of sequencing costs will facilitate future adoption of the platform in clinical microbiology laboratories.

**Acknowledgment:** This research was funded by research university grant GUP-2017-003 from Universiti Kebangsaan Malaysia

## O-43 Isolation and production of recombinant monoclonal antibody proteins against a *Toxocara canis* antigen using phage display technology

### Zamrina Baharudeen and Anizah Rahumatullah

#### Institute for Research in Molecular Medicine (INFORMM), Universiti Sains Malaysia, Penang, Malaysia

##### **Correspondence:** Anizah Rahumatullah (anizahrahumatullah@usm.my)

From The International Conference on Molecular Diagnostics & Biomarker Discovery 2022 (MDBD 2022)

Penang, Malaysia. 11 – 13 October 2022

**Background**

Toxocariasis is a neglected zoonotic parasitic disease which mainly caused by *Toxocara canis*, an intestinal parasitic roundworm of dog. Human toxocariasis has a global distribution, with people from socioeconomically deprived populations more adversely affected. The limitations of diagnosing human toxocariasis is the signs and symptoms of the disease are non-specific and similar to other helminth infection [1]. This may lead to misdiagnosis thus underestimating the actual global impact of the disease. Existing serology-based detection method mainly rely on IgG antibody-based assays that suffers from the difficulty in differentiating past and active infections. The assays also lack of high diagnostic specificity due to cross-reactivity with antibodies to other helminths [2]. In the present study, novel recombinant monoclonal antibodies were isolated and the binding of the proteins were verified.

**Methodology**

Monoclonal antibody against recombinant *Toxocara* excretory-secretory 26 antigen (rTES-26) was isolated utilizing previously reported helminth phage display library via biopanning. Polyclonal and monoclonal phage ELISA were carried out at the end of selection. Positive clones were sent for sequencing and the scFv antibody clones verified using IMGT/V-QUEST bioinformatics tool. Selected positive monoclonal antibody clones with complete scFv gene were subcloned into pET-51b(+) and transformed into SHuffle® T7 Express *Escherichia coli* host cell. Antigen-antibody binding assays were performed using rTES-26 and native *T. canis* antigens.

**Results and Discussion**

The significant increase in absorbance reading of polyclonal ELISA (OD_405_ ranging from 0.321 to 3.020) indicating rTES-26 specific phage antibodies successfully enriched. In total 384 individual clones were screened during monoclonal ELISA and five positive binders were identified with absorbance values ranging from 0.45 to 3.91. Three clones from distinct gene families (Ab 48: IgHV3-LV1; Ab 49:IgHV3-LV3; Ab 50: IgHV6-LV3) were selected, however only two clones: Ab 48 and Ab 49 demonstrated complete insertion of the full-length scFv antibody sequence following sub-cloning. Both clones were successfully expressed and purified at satisfactory levels in terms of purity and yield. The antigen-antibody binding analyses showed that both Ab 48 and Ab 49 able to bind the recombinant and native form of *Toxocara* protein. However, in total Ab 49 showed better performance in protein yield and binding affinity compared to Ab 48. Hence, the enriched monoclonal antibodies from the immune libraries shows to have varying binding affinities and specificities [3].

**Conclusion**

In summary, both antibody clones showed their diagnostic potential and the binding evaluation suggest that Ab 49 has the potential to be used as an efficient tool for human toxocariasis diagnosis.

**References**

[1] Noordin, R., et al. Serodiagnostic methods for diagnosing larval toxocariasis, Adv. in Parasit. 2020, 109: 131-152.

[2] Rostami, A., et al. Human toxocariasis – A look at a neglected disease through an epidemiological ‘prism’. Infect. Genet. Evol. 2019, 74:104002.

[3] Georgiou, G., et al. The promise and challenge of high-throughput sequencing of the antibody repertoire. Nat. Biotechnol. 2014, 32:158-168.

## O-44 Optimization of transient expression of recombinant IgG binding protein, FcγRIIa

### Shin Yi Gan, Gee Jun Tye, Ai Lan Chew and Ngit Shin Lai

#### Institute for Research in Molecular Medicine (INFORMM), Universiti Sains Malaysia, Penang, Malaysia

##### **Correspondence:** Ngit Shin Lai (laingitshin@usm.my)

From The International Conference on Molecular Diagnostics & Biomarker Discovery 2022 (MDBD 2022)

Penang, Malaysia. 11 – 13 October 2022

**Background**

IgG Fc binding proteins (FcγRs) are immunoglobulin receptors that particularly bind to the human IgG (hIgG) molecules through its Fc portion. Among the members of FcγRs, FcγRIIA is slowly gaining interest from scientific community due to its different isotype specificities and the significant binding affinity towards IgG2. Current technology has presented the production of recombinant proteins in mammalian cell lines to introduce appropriate protein folding and post-translational modifications [1]. However, the production of recombinant proteins using mammalian expression systems is laborious, time-consuming, and costly [1]. Therefore, this study provided a simple protocol for optimized transient transfection and expression of recombinant IgG binding proteins to achieve a high yield in a short span of time.

**Methodology**

FreeStyle™ 293-F cells in the prewarmed FreeStyle™ 293 Expression Medium were incubated in a 37°C incubator containing a humidified atmosphere of 5% CO_2_ in air on an orbital shaker platform rotating at 120 rpm. On the day of transfection, the cells were suspended in a volume of prewarmed medium to obtain a concentration of 1.0×10^6^ cells/mL. For each mL of transfection volume, 1μg of plasmid DNA and 2μL of 293fectin diluted in Opti-MEM were needed. After adding the DNA/293fectin mixture, the suspension cells were incubated for 96 hours. The cell pellets were harvested by spinning down the cells for 5 mins at 300 ×g and the intracellular protein was harvested by using lysis buffer. After the protein has been purified using immobilized metal affinity chromatography (IMAC), the protein was subjected to sodium dodecyl sulphate-polyacrylamide gel electrophoresis (SDS-PAGE) and Western Blot analyses for the visual evaluation of expression. The protein concentration was measured by using Nanodrop Spectrophotometer.produced in less than one week. In general, the recombinant proteins with expression levels up to 100 mg/L can be obtained. Since the expression level may differ depending on the nature of the recombinant proteins [2], the transfection method had been refined by varying the incubation time post-transfection, ending up with an ideal harvest time of 96 hours. The harvested protein was purified by using IMAC for downstream analysis. In SDS-PAGE analysis, an intense protein band at approximately 38 kDa had been observed, indicating the expression of the recombinant proteins. Meanwhile, in Western blot analysis, anti-histidine antibody was used to detect the hexa-histidine tagged recombinant protein.

**Conclusion**

An optimized protocol that serves as a general guide for the transient transfection and expression of recombinant IgG binding protein, FcγRIIA had been described. The simplicity of this protocol had exhibited an efficient transfection and a fast yet high-yield expression of proteins.

**Acknowledgement:** This work was funded by Malaysian Ministry of Education through the Higher Institution Centre of Excellence (HICoE) Grant No: 311/CIPPM/4401005.

**References**

[1] Hunter M, et al. Optimization of Protein Expression in Mammalian Cells. Curr. Protoc. Protein Sci. 2019, 95:e77.

[2] Vink T, et al. Robust and Highly Efficient Transient Expression System for Producing Antibodies. Methods. 2014, 65:5-10.

## O-45 Cloning of IgM Fc receptor for mammalian expression system

### Hai Shin Pung, Gee Jun Tye and Ngit Shin Lai

#### Institute for Research in Molecular Medicine (INFORMM), Universiti Sains Malaysia, Penang, Malaysia

##### **Correspondence:** Ngit Shin Lai (laingitshin@usm.my)

From The International Conference on Molecular Diagnostics & Biomarker Discovery 2022 (MDBD 2022)

Penang, Malaysia. 11 – 13 October 2022

**Background**

Mammalian expression system has become routine in the production of pharmaceutical protein as it offers post-translational modifications (PTMs) which allows production of complex proteins and glycosylated enzymes where their native structures and activities are preserved. In order to express the recombinant protein in mammalian cell lines efficiently, selection of an appropriate expression vector that carry the interested recombinant gene plays an important role. Polymeric immunoglobulin receptor (pIgR), an IgM Fc receptor is chosen to be expressed due to its various clinical potentials such as the possibility to serve as predictive biomarker target owing to its correlation with various cancer malignancies, potential role, and underlying mechanisms in body immunity [1, 2].

**Methodology**

The plasmids that carried *PIGR* gene were purified from the bacterial overnight culture, followed by polymerase chain reaction (PCR) to amplify the interested DNA segment. The PCR products and mammalian expression vector (pcDNA5/FRT) were then subjected to restriction digestion and ligation process prior to bacteria transformation. The final product was sent for sequencing before the mammalian cells transfection.

**Results and Discussion**

The presence of bacterial colonies in ampicillin-containing agar plate indicating the success of bacteria transformation. The cloning of the gene of interest into the mammalian expression vector (pcDNA5/FRT) was then verified through restriction digestion, showing two bands where both represented the vector backbone (5002bp) and *PIGR* gene sequence (2490bp) respectively. The sequence was checked for any point mutation through comparison of each nucleotide with the reference sequence. Flp-In System involves the introduction of Flp Recombination Target (FRT) site into genome of both expression vector and mammalian cell line of choice. The expression vector that carrying the gene of interest will integrate into the host cell genome through Flp recombinase-mediated DNA recombination at the FRT site. This system allows stable expression cell lines to be created, providing advantages such as rapid and efficient protein expression by the subsequent generation of Flp-In cell lines [3].

**Conclusion**

Gene cloning is an important step for the protein expression by living cells, especially for mammalian cells. The quality and sequences of the recombinant plasmid will impact the efficiency of protein expression. In future, the constructed recombinant plasmid (pcDNA5/FRT-PIGR) will be transfected into chosen mammalian cell line to express the recombinant protein as the sequence were checked accordingly. Antibiotic selection will be performed to select cells where the plasmids are integrated into genome successfully. Single cells that are able to survive upon transfection and selective growth will be expanded to generate a clonal population. Stable clones are selected after screening and ready for protein production.

**Acknowledgement:** This work was funded by Malaysian Ministry of Education through the Higher Institution Centre of Excellence (HICoE) Grant No: 311/CIPPM/4401005.

**References**

[1] Yue X, et al. Polymeric Immunoglobulin Receptor Promotes Tumor Growth in Hepatocellular Carcinoma. Hematology. 2017, 65:1948-1963.

[2] Zhou M, et al. Expression of polymeric immunoglobulin receptor and its biological function in endometrial adenocarcinoma. J. Can. Res. Ther. 2019, 15:420-425.

[3] Phan QV, Contzen J, Seemann P, Gossen M. Site-specific chromosomal gene insertion: Flp recombinase versus Cas9 nuclease. Sci Rep. 2017, 7.

## O-46 New β-carboline compound as a promising anticancer agent in chronic myelogenous leukemia (CML)

### Meroshine Nageswara Rao^1^, Mazlin Mohideen^2^, Leong Sze Wei^3^, Thiruventhan Karunakaran^4^ and Nur Azzalia Kamaruzaman^1^

#### ^1^ National Poison Centre, Universiti Sains Malaysia, 11800 USM Penang, Malaysia; ^2^ Faculty of Pharmacy and Health Sciences, Universiti Kuala Lumpur Royal College of Medicine Perak, 30450 Ipoh, Perak, Malaysia; ^3^ Chemistry Department, Faculty of Science, Universiti Malaya, 50603 Kuala Lumpur, Malaysia; ^4^ Centre for Drug Research, Universiti Sains Malaysia, 11800 USM Penang, Malaysia

##### **Correspondence:** Nur Azzalia Kamaruzaman (azzalia@usm.my)

From The International Conference on Molecular Diagnostics & Biomarker Discovery 2022 (MDBD 2022)

Penang, Malaysia. 11 – 13 October 2022

**Background**

Cancer is known as one of the serious causes of deaths among the world population and it is believed that the number of cases to rise over the years. Chemotherapy drugs are considered as the most reliable and common treatment for cancer. However, these drugs have limitations in terms of effectiveness and severe side effects. In addition, drug resistance is another factor that hinders successful treatment rate among patients. Therefore, finding a lead compound for cancer which addresses these issues are of utmost important. In the constant search for new anticancer agent alternatives, β-carboline has been identified as a good candidate due to its various reported health indications, particularly in anticancer. Therefore, the aims of the study are to investigate the potential of a newly synthesized β-carboline compound in eliciting anticancer activity in chronic myelogenous leukemia (CML) and to determine the apoptosis mechanism induced by the compound.

**Methodology**

β-carboline compound (M25) was synthesized and confirmed using gas chromatography-mass spectrometry (GC-MS) analysis. MTT assay was conducted to evaluate cytotoxicity effect of M25 against CML cells (K562 cell line). Toxicity of M25 was determined by calculating its selectivity index (SI), through human fibroblasts (Hs27 cell line) and mouse fibroblasts (BALB/c3T3). Mode of cell death induced by M25 was further evaluated through the apoptosis assay using flow cytometry analysis with Annexin V-FITC and Propidium iodide (PI). Lastly, *in silico* analysis was conducted to study the binding affinity between M25 and identified proteins involved in the apoptosis pathway using Discovery Studio software.

**Results and Discussion**

β-carboline compound M25 was synthesised with high purity and high yield. M25 induced cytotoxicity in K562 cell line with a low IC_50_ value of 0.8 μM. This indicated high potency of M25 in killing human CML cells. Based on IC_50_ values for non-cancer cells Hs27 and BALB/c3T3, the calculated SI were 16 and 25 respectively. The high selectivity showed that M25 is highly specific in killing CML cells, thus reflecting good safety profile for the compound. Flow cytometric analysis also showed that significant number cell death induced by M25 compound was due to apoptosis. In understanding proteins involved in the apoptosis pathway, molecular docking study investigated the binding affinity of M25 as ligand with apoptosis-related proteins such as apoptosis inducing factor (AIF), endonucleases G (Endo G), caspase 3 (CAS 3), caspase 9 (CAS 9) and caspase 8 (CAS 8). Interestingly, the highest binding affinity was recorded for AIF and Endo G, which suggested a unique caspase-independent pathway of apoptosis.

**Conclusion**

The newly synthesized M25 has excellent potential as an anti-leukemic agent as it has shown high cytotoxicity activity and selectivity for K562, thus indicating good effectiveness and safety profile. In addition, in silico study suggested that M25 induced caspase-independent apoptosis, with the possible involvement of AIF and Endo G. This unique pathway may address drug resistance issue which has become a hindrance for most conventional chemotherapeutics.

**Acknowledgement:** The study is financially supported by the Ministry of Higher Education Malaysia through Fundamental Research Grant Scheme (FRGS) (FRGS/1/2020/STG01/USM/02/9).

## O-47 Discovery of potent small molecule inhibitor for dengue through *in silico* and *in vitro* approaches

### Norshidah Harun^1, 3^, Leow Chuan Herng^1^, Kamarulzaman Ezatul Ezleen^2^, Abdul Wahab Habibah^2.,^, Ramachandran Vignesh^3^ and Lai Ngit Shin^1^

#### ^1^ Institute for Research in Molecular Medicine (INFORMM), Universiti Sains Malaysia, 11800, Penang, Malaysia; ^2^ School of Pharmaceutical Sciences, Universiti Sains Malaysia, 11800, Penang, Malaysia; ^3^ Universiti Kuala Lumpur-Royal College of Medicine Perak, 30450, Ipoh, Perak, Malaysia

##### **Correspondence:** Lai Ngit Shin (laingitshin@usm.my)

From The International Conference on Molecular Diagnostics & Biomarker Discovery 2022 (MDBD 2022)

Penang, Malaysia. 11 – 13 October 2022

**Background**

Dengue is a catastrophic arboviral disease, globally and locally. At present there are no effective antiviral drugs and only a limited approved vaccine available^1^. The much-needed treatment for dengue has pushed studies to look into strategizing and developing methods to search for prospective inhibitor candidates. Due to the pivotal role of dengue NS2B/NS3 viral protease in the viral replication, the inhibition of this enzyme is considered to be the key strategy for the development of new dengue antiviral drugs^2^. In this present study, we sought to identify and evaluate small molecules by adapting in silico and in vitro approaches in search of potent inhibitor against the dengue NS2B/NS3 protease.

**Methodology**

To that aim, total of 80 small molecules from the Riken NPDepo Authentic library were screened. Based on the highest percentage of inhibition exhibited, the hit compounds were chosen and molecular docking was performed to predict the binding model of the compounds to dengue NS2B/NS3 protease. Then, a chromogenic-based protease inhibition assay was carried out to evaluate whether the selected candidates inhibited dengue protease activity in vitro.

**Results and Discussion**

Based on *in silico* evaluation, computational simulation revealed the free energy of the compound I, II and III are comparable to that of quercetin, a known NS2B/NS3 protease inhibitor. Through the docking evaluation also showed that all three compounds bound to the DENV NS2B/NS3pro near the active site, hence confirming the possible interaction between the compounds and dengue NS2B/NS3pro. Interestingly, our results in the protease inhibition assay carried out indicated that compound I, II and III have the ability to inhibit dengue NS2B/NS3 proteolytic activity.

**Conclusion**

The *in vitro* inhibition results of the three compounds that we obtained from this study, agrees well with the docking outcome, hence, portrays a potential inhibition activity against dengue virus.

**Acknowledgement:** This work was funded by the Malaysian Ministry of Education through the Higher Institution Centre of Excellence (HICoE) Grant No: 311/CIPPM/4401005 and supported by Research University Individual grant from Universiti Sains Malaysia (1001/CIPPM/ 8012305).

**References**

[1] Obi, J. O., Gutiérrez-Barbosa, H., Chua, J. V., & Deredge, D. J. Current trends and limitations in dengue antiviral research. *Tropical Medicine and Infectious Disease*, (2021), *6*(4), 180.

[2] Aguilera-Pesantes, D., Robayo, L. E., Méndez, P. E., Mollocana, D., Marrero-Ponce, Y., Torres, F. J., & Méndez, M. A. Discovering key residues of dengue virus NS2b-NS3-protease: New binding sites for antiviral inhibitors design. *Biochemical and Biophysical Research Communications*, (2017), *492*(4), 631-642.

## O-48 Systematic review on preclinical reports: Titania Nanotube Arrays technology for medical orthopaedic screw implant application

### Tan Chia Yi^1^, Rabiatul Basria S. M. N. Mydin^1^ and Mohd Sharizal Abdul Aziz^2^

#### ^1^Department of Biomedical Science, Advanced Medical and Dental Institute, Universiti Sains Malaysia, 13200 Bertam, Kepala Batas, Pulau Pinang Malaysia; ^2^School of Mechanical Engineering, Engineering Campus, Universiti Sains Malaysia, 14300 Nibong Tebal, Seberang Perai Selatan, Penang, Malaysia

##### **Correspondence:** Rabiatul Basria S. M. N. Mydin (rabiatulbasria@usm.my)

From The International Conference on Molecular Diagnostics & Biomarker Discovery 2022 (MDBD 2022)

Penang, Malaysia. 11 – 13 October 2022

**Background**

Titania Nanotube Arrays (TNA) technology is a promising surface technology for medical implants, especially for orthopedic applications. The long-term stability of a surface technology on a medical implant is frequently associated with osseointegration. This review provides an overview on TNA technology for medical orthopaedic screw implant applications from preclinical reported studies.

**Methodology**

The review was conducted based on PRISMA-P protocol and pre-determined keywords such as “orthopedics implants screw”, “bone implant”, “Orthopaedic Screw Implant” “TNA surface”, “*In vitro”, “Ex vivo” “In vivo*” “Titania Nanotube Array”, “titanium dioxide nanotube arrays”, “TiO_2_ nanotube arrays”, “preclinical studies”, “preclinical testing” and “osseointegration”. Search engine database such as PubMed, Springer Link, Science Direct and Google Scholar were used. The pre-clinical studies published in English language within the past 5 years (2016 till 2021) were included. To broaden the search results, the terms were combined interchangeably by using the Boolena operators ‘and’ or ‘or’. The titles and abstracts of each article were initially pre-screened. Studies involving those in predatory or blacklisted journals were excluded. To ensure transparency, replicability, and reanalysis feasibility, every step of this approach was meticulously recorded. To prevent double duplication, Endnote software version X7 was used as a reference manager to combine the results of all extracted research.

**Results and Discussion**

The search has generally generated 778 articles. In further analysis, 30 articles were finalized according to inclusion and exclusion criteria. Data were further grouped into *in vitro, ex vivo, in vivo* studies. Most preclinical reports discussed the ideal TNA physiochemical properties that enhance cell proliferation, differentiation and surface properties that could contribute to long-term osseointegration activity*.*

**Conclusion**

The systematic reviews reported on TNA’s ideal physiochemical properties are crucial for long-term bone-implant interaction. Knowledge from this study may contribute to the cutting-edge development of biomedical implant technology.

**Acknowledgement:** The authors would like to thank Universiti Sains Malaysia (RUI EKSESAIS 2019 (No:1001/CIPPT/8012338) for sponsoring this work.

## O-49 Clinicopathological association of Chronic Rhinosinusitis with Nasal Polyp (CRSwNP) and periostin expression

### Sakinah Mohamad^1^, Baharudin Abdullah^1^, Wan Faiziah Wan Abdul Rahman^2^ and Najib Majdi Yaacob^3^

#### ^1^Department of Otorhinolaryngology-Head & Neck Surgery, School of Medical Sciences, Universiti Sains Malaysia Health Campus, Malaysia; ^2^Department of Pathology, School of Medical Sciences, Universiti Sains Malaysia Health Campus, Malaysia; ^3^Biostatistics and Research Methodology Unit, School of Medical Sciences, Universiti Sains Malaysia Health Campus, Malaysia

##### **Correspondence:** Sakinah Mohamad (msakinah@usm.my)

From The International Conference on Molecular Diagnostics & Biomarker Discovery 2022 (MDBD 2022)

Penang, Malaysia. 11 – 13 October 2022

**Background**

In the recent years, periostin (POSTN), a gene encoding an extracellular matrix protein with similarity to fasciclin family has emerged as a potential biomarker for various types of cancers. Besides that, POSTN also plays a role in stimulating eosinophil migration and activation in chronic inflammation and immune pathways. Chronic rhinosinusitis (CRS) with nasal polyp (NP) (CRSwNP) is mediated by Th2-/eosinophilic inflammation type of immune response [1]. POSTN has been proposed to be found in NP tissues and contribute to formation of NP in CRS patients. In this study, we investigated the POSTN protein expression in NP tissue and then we determined its association with the clinicopathological features of CRSwNP patients.

**Methodology**

Tissue samples were collected from 24 CRSwNP patients and their clinicopathological features such as demographic data (age, gender, race and smoking history), clinical (history of bronchial asthma and/or atopy, SNOT-22 scores, Lund-Kennedy scores, Lund-Mackay scores), haematological (percentage of serum eosinophils and total IgE) and pathological features (tissue eosinophil count, degree of inflammation, mucosal ulceration, squamous metaplasia, and fibrosis) were evaluated. POSTN protein expression was assessed by immunohistochemical analysis. A single linear regression analysis was performed to find the association of POSTN protein expression with the clinicopathological features.

**Results and Discussion**

Expression of POSTN protein was detected in all 24 NP samples. The mean IHC score POSTN is 7.31. There was no significant association of POSTN protein expression with clinicopathological features (gender, smoking history, history of bronchial asthma

and/or atopy, SNOT-22 scores, Lund-Kennedy scores, Lund-Mackay scores, percentage of serum eosinophils, total IgE, tissue eosinophil count and other histopathological features (*p* value of 0.66, 0.11, 0.64, 0.57, 0.76, 0.17, 0.09, 0.21, 0.66, and 0.57 respectively) except for age *(p*=0.045). Our findings of POSTN expressed in all NP tissue staining the area of subepithelial tissue and infiltrating inflammatory cells was consistent with other study [1-2]. POSTN is produced in fibroblasts, endothelial and epithelial cells by interleukin (IL)-4 and IL-13 stimulation. Fibroblasts is found to be abundant in NP tissue. Tissue eosinophilia (eosinophil count >10 per high power field) is known to be a hallmark for CRSwNP and a predictor for NP recurrence. Ninomiya et al. 2018 found that protein expression associated with severity of CRSwNP but not in our study. Even though we excluded those patients taking steroids which can suppress the periostin production, we should have exclude those who was asthmatic and on any immunomodulator. Lebrikizumab was shown to be effective in controlling exacerbation of asthmatic patients with high POSTN level in their serum [1]. A larger sample size study at multi-centre setting would be more representative of CRSwNP population.

**Conclusion**

Although presence of POSTN is detected within all nasal polyp tissues, however, POSTN protein expression was not significantly associated with clinicopathological features of CRSwNP. Therefore, tissue POSTN appears to have no role in evaluation of patients with CRSwNP.

**Acknowledgement:** The study was funded by the Fundamental Research Grant Scheme (FRGS) (Project number: 203/PPSP/6171237) from the Ministry of Education, Malaysia.

**References**

[1] Ninomiya T, et al. Periostin as a novel biomarker for postoperative recurrence of chronic rhinosinusitis with nasal polyps. Scientific reports. 2018; 8(1): 1-0

[2] Xu M, et al. The role of periostin in the occurrence and progression of eosinophilic chronic sinusitis with nasal polyps.. Scientific reports. 2017, 7(1):1-9

## O-50 Detection of herpes simplex virus-1 by direct immunofluorescence and viral isolation from cerebrospinal fluid

### Ummu Salamah Faisal^1,2^, Ummu Afeera Zainulabid^3, 4^ and Wong Kong Ken^2^

#### ^1^Department of Medical Microbiology, Faculty of Medicine, National Defense University of Malaysia; ^2^Department of Microbiology and Immunology, Faculty of Medicine, National University of Malaysia; ^3^Deparment of Internal Medicine, Faculty of Medicine, National University of Malaysia Hospital; ^4^Department of Internal Medicine, Kulliyyah of Medicine, International Islamic University Malaysia

##### **Correspondence:** Ummu Salamah Faisal (ummusalamahfaisal@gmail.com)

From The International Conference on Molecular Diagnostics & Biomarker Discovery 2022 (MDBD 2022)

Penang, Malaysia. 11 – 13 October 2022

**Background**

Herpes simplex virus (HSV) is the human herpesvirus that leads to herpes simplex encephalitis or meningoencephalitis and is frequently lethal if not treated properly. Here, we described a case of a 21-year-old man who presented with acute confusion and abnormal behaviour and was later diagnosed with HSV-1 meningoencephalitis based on immunofluorescence and viral isolation from cerebrospinal fluid.

**Methodology**

A lumbar puncture was performed immediately during admission to the ward. Cerebrospinal fluid was also sent for viral culture. The culture was inoculated into human cells in culture (HEp-2) cell monolayers and observed for cytopathogenic effect (CPE). Then the slide was prepared for direct immunofluorescence staining using fluorescein isothiocynate-conjugated HSV type 1 and HSV type 2 antisera. Positive findings would demonstrate cells with fluorescent staining, whereas negative specimens would demonstrate cells with a reddish-brown counterstain. Informed consent to publish had been obtained.

**Results and Discussion**

Brain MRI was performed for further evaluation, which showed a focal area of gyral thickening at the left frontoparietal lobes with leptomeningeal enhancement at the left Sylvian fissure, suggestive of meningoencephalitis, and no hydrocephalus was noted. The CSF results revealed 0 polymorphs cells/mm^3^ and lymphocyte count, with 0 pus cells. CSF biochemistry showed glucose of 1.67 mg/dl and a very high total protein of 1596 mg/dl. The results of viral culture were obtained on admission day ten. After 10 days of culture with daily CPE observation, CPE evidence of HSV was detected. The prepared slide was observed under the ultraviolet microscope and revealed positive for HSV-1 and negative for HSV-2. HSV-1 has accounted for more than 90% of all herpes simplex encephalitis cases in adults and children. It spreads by oral contact and primarily results in cold sores, while HSV-2 is sexually transmitted and causes genital herpes.

**Conclusion**

It has been proven that immunofluorescence antigen detection is a quick, accurate, and sensitive method for distinguishing HSV-1 and HSV-2 antigen in the cerebrospinal fluid of those infected individuals.

## O-51 Development of cisplatin-resistant urothelial cancer cells using pulse-shock treatment

### Siti Farizan Mansor^1, 2^, Abhi Veerakumarasivam^3^ and Badrul Hisham Yahaya^2^

#### ^1^Faculty of Health Sciences, Universiti Teknologi MARA, Cawangan Pulau Pinang, Kampus Bertam, 13200 Pulau Pinang, MALAYSIA; ^2^Department of Biomedical Sciences, Advanced Medical and Dental Institute (AMDI), Universiti Sains Malaysia, Kepala Batas Penang, 13200 MALAYSIA; ^3^Department of Biological Sciences, School of Medical and Life Sciences, Sunway University, MALAYSIA

##### **Correspondence:** Badrul Hisham Yahaya (badrul@usm.my)

From The International Conference on Molecular Diagnostics & Biomarker Discovery 2022 (MDBD 2022)

Penang, Malaysia. 11 – 13 October 2022

**Background**

Cisplatin has been used in chemotherapy of advanced urothelial carcinoma (UC) that is no longer amenable to local treatment such as surgery and radiotherapy. However, almost 30% of UC patients develop resistance to chemotherapy and recurrence within 2 years post- therapy. The understanding of the mechanism of cisplatin resistance in UC patients is scarce. In pulse-shock chemotherapy, cancer cells are subjected to chemodrug treatment in a short period of time.

**Methodology**

In this study, cisplatin-resistant UC model was developed and analysed for morphological and transcriptomic changes. Firstly, cisplatin IC50 dosage of a urothelial cancer cell line, 5637 cells was determined. Cells were then subjected to repeated pulse-shock treatment with the respective IC50 dosage and morphological changes will be recorded. At the end of the repeated treatment cycles, IC50 of the cisplatin-resistant cell was compared to its parental counterpart and transcriptomic changes in drug resistance genes were analysed.

**Results and discussion**

When pulse-shock treated for 2 hours, cisplatin IC50 dosage of 5637 cells was 11uM. After sixth treatment cycle, IC50 was significantly increased to 21uM, which was 2 times higher compared to parental cells. During the course of treatment, morphological changes vary from polyploid giant cells, MSC-like cell, parental cells and bizarre shaped cells. ABCA1, ABCG2, Bax and BIRC5 genes were found to be significantly upregulated in cisplatin-resistant 5637 compared to parental cells. Stress induction through pulse-shock cisplatin treatment promotes cancer plasticity through transformation into polyploid giant cells. These polyploid giant cells has been reported to drive cancer progression through activation of EMT, drug resistance, apoptotic inhibition and many more. Molecular mechanism and regulation of polyploid giant cells are not fully understood, hence, an exciting niche awaiting exploration.

**Conclusion**

The model has a potential to be used for evaluation of existing and novel targets for recurrent therapeutic regimes, thus making UC management more efficient, convenience and increased patients’ quality of life.

**Acknowledgement:** This study was funded by Ministry of Higher Education Malaysia through Fundamental Research Grant Scheme FRGS/1/2019/SKK15/UITM/03/1, Project ID 15531, RMC file number 600- IRMI/FRGS 5/3 (286/2019).

## O-56 Selection of ssDNA aptamers against Programmed Death-Ligand 1 (PD-L1)

### Muhammad Najmi Mohd Nazri, Khairul Mohd Fadzli Mustaffa and Noor Fatmawati Mokhtar

#### Institute for Research in Molecular Medicine (INFORMM), Health Campus, Universiti Sains Malaysia, 16150 Kubang Kerian, Kelantan, Malaysia

##### **Correspondence:** Noor Fatmawati Mokhtar (fatmawati@usm.my)

From The International Conference on Molecular Diagnostics & Biomarker Discovery 2022 (MDBD 2022)

Penang, Malaysia. 11 – 13 October 2022

**Background**

Recently, serum PDL1 expression has been demonstrated to be a potential biomarker for cancer patient’s treatment response and outcome or can even be used for early cancer detection. The most common biomarker detection approach utilizes antibody-based, but the use of antibody face numbers of drawback. Consequently, another potential alternative to antibody known as aptamers have become increasing popular. Aptamers exhibit characteristic that can overcome the drawbacks of antibody such as lower production cost, minimal batch-to-batch variation, possess better thermal stability and offers variety of chemical modification. Thus, this study aims to isolate ssDNA aptamers against recombinant human PD-L1 for future diagnostic purpose.

**Methodology**

In this study, we demonstrated the isolation of ssDNA aptamers against rhPD-L1 via agarose bead-based Systematic Evolution of Ligands by Exponential Enrichment (SELEX). The SELEX enrichment monitoring was determined by direct Enzyme-Linked Oligonucleotide Assay (ELONA). The enriched SELEX cycle was then cloned, followed by sanger sequencing to identify the sequence of potential aptamers. The sequences were analysed using MegaX software, online mFold software and online MEME (Multiple Em for Motif Elicitation).

**Results and Discussion**

Total of 9 SELEX cycle was performed and SELEX cycle 6 was selected as an enriched cycle. Pool of ssDNA from SELEX cycle six was cloned and a total of 36 positive colonies were sequenced. Phylogenetic analysis of the positive colonies yields 9 clusters distinct aptamers. Further analysis of Gibbs free energy, frequency of a sequence and significant motif of all the sequences revealed 3 potential aptamer sequence.

**Conclusion**

These 3 potential aptamer binding affinities should be characterized as it could be potential biorecognition element targeting the PD-L1 for the diagnostic application.

**Acknowledgement:** This research was funded by the Malaysian Ministry of Education through the Higher Institution Centre of Excellence (HICoE) Program (No. 311/CIPPM/4401005).

## P-1 Suppression of microRNA-9 promotes anti-tumour activity in KMS-28BM human multiple myeloma cells

### Ivyna Pau Ni Bong, Nor Soleha Mohd Dali, Norodiyah Othman^1^, Aliza Mohd Yacob^1^ and Ezalia Esa^1^

#### Haematology Unit, Cancer Research Centre, Institute for Medical Research, National Institutes of Health, Ministry of Health Malaysia

##### **Correspondence:** Ivyna Pau Ni Bong (ivyna@moh.gov.my)

From The International Conference on Molecular Diagnostics & Biomarker Discovery 2022 (MDBD 2022)

Penang, Malaysia. 11 – 13 October 2022

**Background**

Multiple myeloma (MM) is the second most common blood cancer characterised by clonal expansion of malignant plasma cells within the bone marrow [1]. Epigenetic aberrations such as microRNAs (miRNAs) dysregulation are of great significance in the development and malignancy of MM. MiRNAs are short, single stranded non-coding RNAs (~19-25 nucleotides) that play an important role in regulating post-transcriptional expression of target genes. Up-regulation of miR-9 has been reported in various cancers including MM; however, the mechanisms underlying its aberrant expression and functional alterations in MM are still unclear [2].

**Methodology**

This study aims to investigate the functional role of miR-9 in MM cells *in vitro*. KMS-28BM MM cells were transfected with miRNA-9 inhibitors or miRNA inhibitors scrambled negative control. The expression levels of miR-9 vs control were determined by RT-qPCR. The effect of miRNA-9 suppression on cell proliferation was assessed by MTS assay. Flow cytometry analysis using Annexin V-FITC/PI double staining was used to measure the proportion of apoptosis cells.

**Results and Discussion**

The RT-qPCR results demonstrated that miR-9 inhibitors were successfully delivered and suppressed the miR-9 expression level in KMS-28BM compared to the control (*P*<0.05). Furthermore, MTS and flow cytometry analysis revealed that suppression of miR- 9 expression significantly decreased cell growth and increased the number of early apoptosis cells in KMS-28BM, respectively (*P*<0.05). Our previous microarray-based miRNA expression profiling results showed that miR-9 is up-regulated in the majority of MM patients and cell lines, suggesting miR-9 plays a pivotal role, at least in part, in myelomagenesis. The present study findings further confirm that miR-9 functions as an oncogene in MM pathogenesis, and suppression or silencing of miR-9 inhibits cell proliferation and induces apoptosis in KMS-28BM MM cells.

**Conclusion**

Suppression of miR-9 promotes anti-tumour activity by inhibiting proliferation and inducing apoptosis in MM cells indicates that miR-9 may be a potential therapeutic target in MM.

**Acknowledgement:** This work was funded by a research grant from the Ministry of Health, Malaysia (NMRR-18-729-41414).

**References**

[1] Heider, et al. Multiple Myeloma: Molecular pathogenesis and disease evolution. Oncol Res Treat. 2021, 44(12):672-681.

[2] Khosravi, et al. The impact of mir-9 regulation in normal and malignant hematopoiesis. Oncol Rev. 2018, 12(1):348.

[3] Bong IPN, et al. microRNA expression patterns and target prediction in multiple myeloma development and malignancy. Genes Genomics. 2017, 39(5):533-540.

## P-2 Association of oxidative damage measured by 8-hydroxyguanosine formation with altered risks to hepatocellular carcinoma in Malaysian study population

### Zalinah Ahmad^1^ and Suzana Makpol^2^

#### ^1^Department of Pathology, Faculty of Medicine and Health Sciences, Universiti Putra Malaysia, 43400 UPM Serdang, Selangor; ^2^Department of Biochemistry, Faculty of Medicine, UKM Medical Centre, Jalan Yaakob Latif, Bandar Tun Razak, Cheras 56000 Kuala Lumpur

##### **Correspondence:** Zalinah Ahmad (zalinah@upm.edu.my)

From The International Conference on Molecular Diagnostics & Biomarker Discovery 2022 (MDBD 2022)

Penang, Malaysia. 11 – 13 October 2022

**Background**

Hepatocellular carcinoma (HCC) is the most devastating type of liver cancer. This disease is the third leading cause of cancer deaths with a 5-year survival rate of 7% [1]. Cancer is the pathogenesis product of DNA damage resulting from multiple factors which among others is oxidative stress. Oxidative stress can be detected by the DNA base damage, through the formation of 8-oxo-7, 8-dihydro-2’deoxyguanosine (8-oxodG) [2]. The 8-oxodG acts as oxidative stress indicator and has been essentially specified as a recognized initiator of the carcinogenic process and premutagenic injury in mammalian cells [3]. In this preliminary study, we investigated the possible association of oxidative DNA damage in a form of 8-oxodG in HCC patients in comparison with Malaysian healthy controls. We also analysed the effect of 8-oxodG in different races and gender in both groups.

**Methodology**

DNA of peripheral white blood cells was isolated from 91 HCC patients and 304 controls. The level of oxi dative DNA damage was determined by highly sensitive ELISA kit produced from Japan Institute for Control of Ageing (JaICA), Nikken Foods. Ltd. The determination of oxidative DNA level was digested by Ravanant (1998) and Evans (1999) methods with some modifications. The results were reported as 8-oxodG per 50 μL DNA sample in ng/ml.

**Results and Discussion**

Quantitative measurement of 8-oxodG was higher in HCC patients at mean value of 3.30 ± 2.32 ng/ml. In controls, the average value is 1.57 ± 1.92 ng/ml. There was a significant difference in the average value of 8-oxodG level between the controls and HCC patients where *p* < 0.001. Comparison between gender showed that there was a significant difference observed in the level of 8-oxodG between male and female in controls (*p* < 0.05). The level of 8-oxodG was higher in male (1.736 ± 2.033) ng/mL than in female controls (1.087 ± 1.433) ng/mL. From previous studies, it was reported that smoking, alcohol consumption and bad sleeping pattern were the sporadic factors for male tendency to get the oxidative damage [4]. Hepatocarcinogenesis has been reported to be associated with DNA damage through the process of oxidation. Oxidation in turn, leads to the huge amount of transversion from G □ T in tumor genes such as p53 gene [5]. In this study, however, Malays and no-Malays showed no significant difference in their level of 8-oxodG indicating no disparity.

**Conclusion**

HCC patients showed greater oxidative damage to DNA as compared to controls. This suggests that oxidative DNA damage may contribute to the pathogenesis of HCC. Since 8-oxodG was higher in males, it is indicating that the males are at a greater risk of developing HCC than the females.

**References**

[1] F. X. Bosch, J. Ribes, M. Díaz, and R. Cléries, “Primary liver cancer: worldwide incidence and trends,” Gastroenterology, vol. 127, no. 5 Suppl 1, 2004, doi: 10.1053/J.GASTRO.2004.09.011.

[2] X. Qing, D. Shi, X. Lv, B. Wang, S. Chen, and Z. Shao, “Prognostic significance of 8-hydroxy-2′-deoxyguanosine in solid tumors: A meta-analysis,” BMC Cancer, vol. 19, no. 1, pp. 1–15, Oct. 2019, doi: 10.1186/S12885-019-6189-9/FIGURES/8.

[3] I. Liguori et al., “Oxidative stress, aging, and diseases,” Clin. Interv. Aging, vol. 13, pp. 757–772, Apr. 2018, doi: 10.2147/CIA.S158513.

[4] H.-Y. Huang, K. J. Helzlsouer, and L. J. Appel, “The Effects of Vitamin C and Vitamin E on Oxidative DNA Damage: Results from a Randomized Controlled Trial1,” Cancer Epidemiol. Biomarkers Prev., vol. 9, no. 7, pp. 647–652, 2000.

[5] M. L. Hamilton et al., “A reliable assessment of 8-oxo-2-deoxyguanosine levels in nuclear and mitochondrial DNA using the sodium iodide method to isolate DNA,” Nucleic Acids Res., vol. 29, no. 10, p. 2117, May 2001, doi: 10.1093/NAR/29.10.2117.

## P-3 Mechanisms of optical light properties using optical spectroscopy for various honey detection

### Nurul Syazana Ramlee^1^, Nur Izzah Atirah^1^, Irneza Ismail^1^, Fatin Hamimi Mustafa^2^, Wan Zakiah Wan Ismail^1^ and Juliza Jamaludin^1^

#### ^1^Faculty of Engineering and Built Environment, Universiti Sains Islam Malaysia, Nilai, Negeri Sembilan, Malaysia; Institute for Research in Molecular Medicine (INFORMM), Universiti Sains Malaysia Health Campus Kubang Kerian, Kelantan, Malaysia

##### **Correspondence:** Irneza Ismail (dr.irneza@usim.edu.my)

From The International Conference on Molecular Diagnostics & Biomarker Discovery 2022 (MDBD 2022)

Penang, Malaysia. 11 – 13 October 2022

**Background**

Honey is a natural sweetener made by honeybees from plant nectar that has been consumed by humans for thousands of years [1]. There are two kinds of honey which are monofloral honey and multifloral honey. Monofloral honey is made from a single plant nectar that has been transformed into honey. It includes Acacia, Sidr, and Kelulut honey. Meanwhile, multifloral honeys like Tualang, Manuka, and Kelulut honey are made from a blend of several different plant nectar types found in deep forests or mixed by the beekeeper before being converted into honey [2]. Honey consumption in Malaysia is increasing due to its various nutrient contents that are beneficial to one’s health. Different types of honey contain varying amounts of sucrose, fructose, and glucose, which contribute to the sweetness of honey.

**Methodology**

In this study, pure Tualang, Kelulut, Acacia, Manuka, and Sidr honey samples were used. Optical spectroscopy in the ultraviolet-visible-near infra-red (UV-VIS-NIR) range was used to observe the optical properties of all of the honey, including absorbance and transmission. The wavelength range covered during the measurement was 350 nm to 1662 nm. The Ocean View software was used to capture the spectra of all honey. To improve the accuracy of the results, the spectra was collected 15 times for each honey. The average of the results were plotted, and boxplot analysis was performed for all spectra results.

**Results and Discussion**

The spectra results were taken based on the wavelength of the optical properties, which were absorbance and transmittance. Based on the observed results, the peak absorbance spectra for all honey in both UV-VIS-NIR were similar. Further analyses were done using boxplot analysis to differentiate the characteristics for all type of honey. Sidr honey has the highest mean absorbance value compared to other honeys. The current study found that all Malaysian raw honey met the international standard because the sum of their fructose and glucose contents were less than 60% [3]. But since Manuka and Sidr honey originated outside of Malaysia, it was assumed that both honeys do not adhere to the standard and thus consisted a higher sugar content compared to other honey. According to the Beer Lambert Law equation, increasing the sugar content will increase the number of molecules in the solution, which will increase the absorbance value. Based on previous research, an increasing pattern in absorbance value was highly noticeable as sugar concentration increased. Maximum transmission spectra for all honey in both ranges were close for both UV-VIS and VIS-NIR ranges. From the transmission boxplot analysis, Sidr honey recorded the lowest mean compared to other honeys. As a result, when the concentration of the molecules increased, the transmission value decreased, giving Sidr honey the lowest mean among the others.

**Conclusion**

Two optical properties, which are absorbance and transmission, were studied for all of the honey using optical spectroscopy in both the UV-VIS and VIS-NIR ranges. A boxplot analysis was performed on the spectra results to compare the mean value of the peak spectra. Sidr honey recorded the highest mean absorbance value compared to other honeys since it has the highest sugar content. According to Beer Lambert’s law, as the concentrations of molecules increase, the absorbance value increases but the transmission value decreases. Therefore, Sidr honey has the lowest transmission mean value compared to other honeys.

**References**

[1] Mărgăoan, Rodica, E. Topal, R. Balkanska, B. Yücel, T. Oravecz, M. Cornea- Cipcigan and a. D. C. Vodnar, “Monofloral honeys as a potential source of natural antioxidants, minerals and medicine,” Antioxidants 10, p. no. 7, 2021

[2] Mureșan, C. Ioana, M. Cornea-Cipcigan, R. Suharoschi, S. Erler and a. R. Mărgăoan, “Honey botanical origin and honey-specific protein pattern: Characterization of some European honeys,” LWT 154, p. 112883, 2022.

[3] T.K. Zae, A. Azlan, A.B. Sajak, E.K. Hock, L. C. Nyuk, M. Noh, M. Khalid, “Comparison of selected local honey with Manuka honey based on their,” Food Research 4, pp. 205-213, 2020.

## P-4 The effects of small molecules chaperone on cell viability in primary neonatal fibroblast cell line: Toxicity indicator for pharmacological chaperone therapy

### Affandi Omar^1, 3^, Muhammad Nor Farhan Saat^2^, Fatimah Diana Amin Nordin^1^, Salina Abdul Rahman^1^, Balqis Kamarudin^1^, Nur Jannaim Muhamad^1^, Mohd Shihabuddin Ahmad Noorden^3^ and Julaina Abdul Jalil^1^

#### ^1^Inborn Errors of Metabolism & Genetics Unit, Institute for Medical Research, National Institutes of Health, Ministry of Health Malaysia, Shah Alam, Selangor, Malaysia; ^2^Bioassay Unit, Institute for Medical Research, National Institutes of Health, Ministry of Health Malaysia, Shah Alam, Selangor, Malaysia; ^3^Faculty of Pharmacy, Universiti Teknologi Mara, Puncak Alam Campus, Selangor, Malaysia

##### **Correspondence:** Affandi Omar (fendi.omar@moh.gov.my)

From The International Conference on Molecular Diagnostics & Biomarker Discovery 2022 (MDBD 2022) Penang, Malaysia. 11 – 13 October 2022

**Background**

Hunter syndrome (HS) is a lysosomal storage disorder (LSD) caused by a mutation in the gene *IDS*, which produces the defective enzyme iduronate-2-sulphatase (IDS). Due to the limitations of enzyme replacement therapy for the treatment of HS, the use of small molecule compounds as pharmacological chaperone therapy (PCT) has recently been widely investigated [1]. Usually, small molecules with higher binding affinity that can be used at low concentrations level will be chosen as pharmacological chaperone candidates [2]. As a result, *in vitro* assays such as methyl tetrazolium test (MTT) assay can be used to determine the cytotoxicity of small molecules before proceeding with *in vivo* studies. Therefore, the aim of this study is to investigate the cytotoxic effects of selected small molecules on the viability of primary neonatal fibroblast cell line.

**Methodology**

Selected small molecules of heparin tetrasaccharide, (H004), heparin octasaccharide (H008), heparin decaoctasaccharide (H018), heparin disaccharide standard (HD001) and chondroitin dermatan trisulphate (CD007) were diluted in minimum essential medium (MEM) with several concentrations (0.16-500 μM). The BJ CRL-2522 fibroblast cell lines were seeded into 96-well cell culture plate at a density of 1x104 cells/well before the medium was removed from the cells after 24 hours. Subsequently, the cell lines were treated with the medium containing the respective small molecules for 72 hours (5% CO_2_, 37oC). The cell viability was assessed by MTT assay, and the half maximal inhibitory concentration (IC_50_) was calculated to determine the toxicity of small molecules.

**Results and Discussion**

The cell viability was decreased in a dose- and time-dependent manner when treated with the respective small molecules. HD001 has significantly exhibited concentration and time- dependent inhibitory effect to the cells with the highest IC_50_ of 239 μM. H018 showed IC_50_ of 18.82 μM making it the most toxic among all small molecules tested. The IC_50_ of H004, H008 and CD007 were 83.52 μM, 68.13 μM and 72.03 μM, respectively. Despite having non-toxic outcomes, HD001’s chaperone action is inadequate to serve as a pharmacological chaperone because it binds to the IDS at a high concentration (inhibition constant, K_i_ 431.4 μM). [2]. On other hand, the high binding affinity of CD007 has a greater chaperone effect (K_i_, 21 μM) compared with HD001 with a compromised moderate toxicity level towards the cell lines.

**Conclusion**

This study concludes that CD007 could be considered as potential pharmacological chaperone for PCT in HS based on its safety profile.

**Acknowledgement:** This work was funded by a research grant from the Ministry of Health, Malaysia (NMRR-20-669-54509).

**References**

[1] Parenti Giancarlo, *et al*. Pharmacolohical chaperone Therapy: Preclinical development, Clinical translation, and prospects for the treatment of Lysosomal Storage Disorders. Molecular Therapy. 2015; 23(7): 1138-1148.

[2] Muntau Ania C, *et al*. Innovative strategies to treat protein misfolding in inborn errors of metabolism: pharmacological chaperones and proteostasis regulators. J Inheri Metab Dis. 2014; 37(4): 505-523

## P-5 Decrease in mitochondrial dynamics in a patient with energy deficiency disorder

### Fatimah Diana Amin Nordin^1, 2^, Pung Yuh Fen^2^, Affandi Omar^1^, Nur Azian Aziz^1^, Izyan Mohd Idris^1^, Rosnani Mohamed^1^, Balqis Kamarudin^1^, Nur Jannaim Muhamad^1^ and Julaina Abd Jalil^1^

#### ^1^IEM & Genetics Unit, NMCRC, Institute for Medical Research, National Institutes of Health, Ministry of Health, Shah Alam, Malaysia; ^2^Division of Biomedical Science, Faculty of Science and Engineering, University of Nottingham Malaysia, Semenyih, Malaysia

##### **Correspondence:** Fatimah Diana Amin Nordin (fatimahdiana@moh.gov.my)

From The International Conference on Molecular Diagnostics & Biomarker Discovery 2022 (MDBD 2022)

Penang, Malaysia. 11 – 13 October 2022

**Background**

Mitochondrial dynamics refers to the coordination of fission and fusion process in mitochondria for the maintenance of their distribution, shape and size. Mitochondrial dynamics is crucial in the regulation of cell cycle, immunity, apoptosis and mitochondrial quality control of various processes such as biogenesis and mitophagy. Defective mitochondrial dynamics due to mutations were reported to be linked with human diseases [1]. For example, patients with energy deficiency are suspected of having primary mitochondrial disorders that can be inherited through defects of either nuclear (autosomal-dominant, autosomal recessive, X-linked) or mitochondrial (mtDNA) genes [2]. In this study, we aim to characterize the mitochondrial dynamics of a patient with energy deficient disorder.

**Methodology**

Fibroblasts from a 2-month-old male patient with clinical symptoms of encephalopathy, seizure, cardiomyopathy, severe metabolic acidosis, pulmonary hypertension idiopathic and low level of lactate were cultured and harvested for western blot experiment. Fibroblast from American Type Culture Collection (BJ CRL-2522™) was used as normal control. Total protein was extracted, and the concentration was measured by Bradford assay. Next, samples were probed antibodies with GTPases of the dynamin superfamily. Antibodies used were Mitofusin 1 (Mfn1), Mfn2, Optic Atrophy 1 (Opa1) and Dynamin-related Protein 1 (Drp1). Anti-glyceraldehyde 3-phosphate dehydrogenase (GAPDH) monoclonal antibody was used as normalization control.

**Results and Discussion**

Mitochondrial fusion protein levels of Mfn1; Mfn2; and Opa1 were found to be significantly decreased in this patient’s cells as compared to wild type (WT) (p<0.05). Meanwhile, there were no differences observed in mitochondrial fission protein level of Drp1 (p>0.05) as compared to WT cells. From these results, the reduced fusion proteins may contribute to energy deficiency in this patient. Previous studies suggested that mitochondrial fusion is known to allow functional complementation of mitochondrial DNA, protein and metabolites, whereas mitochondrial fission facilitates mitochondrial transport, mitophagy, and apoptosis [3].

**Conclusion**

Our study suggested a defective mitochondrial dynamic that appeared to affect the fusion process. Further studies such as immunofluorescent staining is highly recommended in order to gain complementary insights into mitochondrial dynamics as well as its involvement in many primary mitochondrial disorders.

**Acknowledgement:** This work was funded by a research grant from the Ministry of Health, Malaysia (NMRR-20-664-54506).

**References**

[1] Tilokani, et al. Mitochondrial dynamics: overview of molecular mechanism. Essays in Biochemistry. 2018; 62(3):341–360

[2] McInnes, et al. Mitochondrial-associated metabolic disorders: foundations, pathologies and recent progress. Nutrition & Metabolism. 2013; 10(63):1-13

[3] Shirendeb, et al. Abnormal mitochondrial dynamics, mitochondrial loss and mutant huntingtin oligomers in Huntington’s disease: implications for selective neuronal damage. Hum Mol Genet. 2011; 20(7):1438-1455

## P-7 Selectivity and storage stability of the aptamer-modified multi-walled carbon nanotubes screen-printed carbon electrode for direct detection of 25-hydroxyvitamin D_2_

### Balqis Kamarudin, Nur Dalila Rizuan, Mohd Azerulazree Jamilan and Mohd Fairulnizal Md Noh

#### Nutrition, Metabolic & Cardiovascular Research Centre, Institute for Medical Research, National Institutes of Health, Ministry of Health, Selangor, Malaysia

##### **Correspondence:** Balqis Kamarudin (balqis.kamarudin@moh.gov.my)

From The International Conference on Molecular Diagnostics & Biomarker Discovery 2022 (MDBD 2022)

Penang, Malaysia. 11 – 13 October 2022

**Background**

Electrochemical biosensors are some of the promising platforms that are low cost, easy to use with the possibility of being integrated into portable devices for point-of-care use. Recently, aptamer-based electrochemical biosensors have received a significant attention because of their advantages such as good stability, high sensitivity and selectivity. The immobilization of the aptamer onto the working electrode is very important so that the target recognition by the aptamer can be transduced into a measurable electrical signal [1]. Here, we described the selectivity and storage stability of an aptamer-based electrochemical biosensors for direct detection of 25 (OH) vitamin D_2_ on the aptamer-modified multi-walled carbon nanotubes screen-printed carbon electrode (MWCNTs-SPCE).

**Methodology**

Aptamer specific to 25 (OH) vitamin D_2_ was immobilized onto the working electrode surface of the MWCNTs-SPCE. The performance of the modified electrodes was observed to determine their selectivity towards 25 (OH) vitamin D_2_. The long-term storage stability of the aptamer-modified MWCNTs-SPCE was investigated by storing the fabricated devices for a period of up to 30 days at 4°C. Electrochemical measurements were carried out using electrochemical impedance spectroscopy (EIS) in a redox probe (0.1 M KCl containing 5.0 mM Fe (CN)_6_^3-/4-^).

**Results and Discussion**

The immobilization of the aptamer was determined through the differences of resistance to charge transfer (Rct) values obtained. The Rct values increased when aptamer was immobilized onto the surface of MWCNTs-SPCE compared to the bare MWCNTs-SPCE. The specific reaction between aptamer and 25 (OH) vitamin D_2_ causing the Rct value to be decreased. However, there were no changes observed when the assay carried out in the absence of the aptamer. This demonstrates that the signals were due to the specific interaction and not to non-specific adsorptions [2,3]. A good linear response was observed for different 25 (OH) vitamin D_2_ concentrations in the range of 0.1 - 30 ng/ml with a detection limit of 0.72 ng/ml. For the long-term storage stability, the aptamer-modified MWCNTs-SPCE showed a consistent response for the first 14 days, then experiences a drop afterwards. Therefore, the aptamer-modified MWCNTs-SPCE was determined to be stable for 14 days at 4°C.

**Conclusion**

The aptamer-modified MWCNTs-SPCE was shown to be selective towards 25 (OH) vitamin D_2_ and had a reasonable shelf-life of around 2 weeks which can be further improved for a better long-term storage.

**Acknowledgement:** This work was funded by a research grant from the Ministry of Health, Malaysia (NMRR-19-3532-52264).

**References**

[1] Khan N. I., et al. A Low-Cost Inkjet-Printed Aptamer-Based Electrochemical Biosensor for the Selective Detection of Lysozyme. Biosensors. 2018; 8, 7:1-18.

[2] Rohrbach F., et al. Label-free Impedimetric Aptasensor for Lysozyme Detection Based on Carbon Nanotube-modified Screen-printed Electrodes. Anal. Biochem. 2012; 421:454-459.

[3] Kara P., et al. Aptamers Based Electrochemical Biosensor for Protein Detection Using Carbon Nanotubes Platforms. Biosensors and Bioelectronics. 2010; 26:1715-1718.

## P-8 Spectrum of *F9* mutations in Malaysian Haemophilia B patients

### Abdullah Aziz, Nursaedah^1^, Lam, KY^1^, Lukman, Izzatul Nabila^1^, Md Afandi, Faridah^2^, Abdul Karim, Faraizah^2,3^, Mohd Fathullah, Tun Maizura^2^, Mat Yusoff, Yuslina^1^ and Esa, Ezalia^1^

#### ^1^Haematology Unit, Cancer Research Centre, Institute for Medical Research, National Institutes of Health, Selangor, Malaysia; ^2^National Blood Centre, Kuala Lumpur, Malaysia; ^3^Department of Pathology, Ampang Hospital, Selangor, Malaysia

##### **Correspondence:** Abdullah Aziz, Nursaedah (nursaedah@moh.gov.my)

From The International Conference on Molecular Diagnostics & Biomarker Discovery 2022 (MDBD 2022)

Penang, Malaysia. 11 – 13 October 2022

**Background**

Haemophilia B (HB) is a rare X-linked bleeding disorder characterized by deficiency in coagulation factor IX (FIX) due to mutation within Factor 9 (*F9*) gene [1]. *F9* gene is located at Xq27.1-q27.2, consists of 8 exons and 7 introns, dispersed across 34 kb length [2]. Severity of HB patients can be classified into mild, moderate and severe based on the coagulation FIX level. Patients with FIX level <1% are classified as severe whereas patients with FIX level 1%-5% and >5% to <40% are classified as moderate and mild, respectively [3]. In Malaysia, there are limited studies on *F9* gene mutations. Thus, this study aimed to identify the mutations within a representative cohort of HB patients in Malaysian population.

**Methodology**

A total of 41 non-familial related HB patients were studied. The patients’ blood samples and clinical details which contained information on factor level, disease severity and inhibitor status were obtained from National Blood Centre and hospitals all over Malaysia. Genomic DNA was extracted from the blood samples using QIAamp® DNA Blood Midi Kit (Qiagen) and subjected to polymerase chain reaction (PCR). Eight different primer sets were used to amplify exon/intron borders and all eight exons of *F9* gene. PCR products were then sequenced using ABI 3730XL DNA analyzer. Sequencing data of patients were aligned against reference sequences, NG_007994 and NM_000133.4 using CLC Main Workbench to identify the mutation and deduce amino acid changes.

**Results and Discussion**

*F9* gene mutations were successfully detected in all patients, which comprise 32 point mutations (26 missense, 5 nonsense and 1 silent), 6 deletions, 1 insertion, 1 insertion-deletion (indel) and 1 splice site mutation. These mutations were distributed throughout all exons, except exon 3 with most of the mutations located in exon 8 (~42%). Five of the discovered mutations in Malaysian HB patients, c.874C>T, c.523_539delinsGAA, c.318_319insGGC, c.230T>G and C.40delC were found to be novel, whereas the remaining variants have been reported by various countries in several online Haemophilia B databases. In our study, two patients developed inhibitor against FIX and the mutations detected in these patients are nonsense mutation (c.1150C>T) and deletion (c.40delC).

**Conclusion**

Our study identified heterogenous mutation profile in HB patients in Malaysian population. These findings are important for genetic confirmation of HB patients, genetic counselling, prediction of inhibitor development and also for carrier screening among family members.

**Acknowledgement:** The work was funded by the Operational Budget from the Ministry of Health, Malaysia.

**References**

[1] Goodeve AC. Hemophilia B: Molecular pathogenesis and mutation analysis. J Thromb Haemost. 2015; 13:1184–95.

[2] Bowen DJ. Haemophilia A and haemophilia B: Molecular insights. Mol Pathol. 2002; 55:1-18.

[3] Rallapalli PM, et al. An interactive mutation database for human coagulation factor IX provides novel insights into the phenotypes and genetics of hemophilia B. J Thromb Haemost. 2013; 11:1329-40.

## P-9 Small RNA signatures from isoniazid-resistant *Mycobacterium tuberculosis*

### Mohd Iskandar Jumat^1^, Jaeyres Jani^2^ and Kai Ling Chin^1^

#### ^1^Faculty of Medicine and Health Science, Universiti Malaysia Sabah, Sabah, Malaysia; ^2^Borneo Medical and Health Research Center, Universiti Malaysia Sabah, Sabah, Malaysia

##### **Correspondence:** Kai Ling Chin (chinkl@ums.edu.my)

From The International Conference on Molecular Diagnostics & Biomarker Discovery 2022 (MDBD 2022)

Penang, Malaysia. 11 – 13 October 2022

**Background**

Tuberculosis (TB) caused by *Mycobacterium tuberculosis* (MTB), remains a significant public health concern worldwide. The emergence of multidrug-resistant TB (MDR-TB) (resistance to at least isoniazid or rifampicin) has significantly jeopardized the treatment and control of TB. Isoniazid-resistant TB (Hr-TB) is much more common than rifampicin-resistant TB (RR-TB) [1]. The regulatory mechanism underlying the emergence of Hr-TB remain to be fully elucidated. Small non-coding RNAs (sRNAs) regulate bacteria essential functions such as survivability and metabolism, among others. Also, the sRNA has been implicated as one of the caused for development of drug resistance [2]. The present study investigates the expression profile of sRNAs in drug-susceptible TB (DS-TB) and Hr-TB strains using next generation sequencing (NGS) with Illumina HiSeq2500 Deep Sequencing Technology for better understanding of the mechanism of resistance to isoniazid in MTB.

**Methodology**

Three DS-TB (SBH49, SBH149, and SBH372) and three Hr-TB strains (SBH365, SBH438, and SBH509) isolated from pulmonary TB patients were cultured in BD BACTEC^TM^ MGIT^TM^ (Becton Dickinson, USA) and their drug susceptibility to first-line antibiotics, i.e., streptomycin, isoniazid, rifampin, and ethambutol were tested with BACTEC^TM^ MGIT^TM^ 960 SIRE kit (Becton Dickinson, USA). Resazurin microtiter assay (REMA) was performed to determine the minimum inhibition concentration (MIC) for isoniazid. Next, RNA from both susceptible and resistant strains were extracted using Masterpure^TM^ Complete DNA and RNA purification kit (Lucigen, USA). The integrity of RNA was determined by Agilent RNA 6000 Nano kit (Agilent Technologies, USA). Small RNA libraries preparation was carried out using NEBNext® Small RNA Library Preparation kit (NEB, UK). Samples were sequenced by single-end reads of 50 base pairs generated through Illumina HiSeq 2500^TM^ in Rapid Run mode. RNA sequences were trimmed for adapter sequence and low-quality reads were filtered out. The sequences were annotated to reference genome (MTB H37Rv) and differential analysis were carried out with CLC Bio Genomic Workbench (version 8). sRNAs target prediction was performed by using TargetRNA2.

**Results and Discussion**

DS-TB strains were susceptible to all first-line antibiotics, while Hr-TB strains only resistance to isoniazid with MIC of 0.125μg/mL for SBH365, and 2mg/mL for SBH 438 and SBH509. A total of 63,252,209, 63,636,812, and 61,148,224 qualified Illumina reads were obtained from DS-TB strains and 75,296,115, 58,965,389, and 67,935,496 reads from Hr-TB strains. The overall *de novo* assembly of sRNA sequence data generated 255, 255, and 255 for all DS-TB strains and 310, 359, and 403 for Hr-TB strains with sRNA length of 18-30bp. Comparative analysis revealed that 728 sRNAs were differentially expressed in the Hr-TB compared with the DS-TB, of which 422 were upregulated and 306 were downregulated. sRNA target prediction using computational method reveals these sRNAs target wide range of mRNAs which involve in toxin production, survivability, and others [3].

**Conclusion**

sRNAs play a major role in the regulation of gene expression and mediates the cellular processes in bacteria. This data demonstrates that sRNA may serve as an invaluable resource for revealing the molecular basis of the regulation of expression associated with the mechanism of isoniazid resistance in MTB.

**Acknowledgement:** The work was funded by the Malaysia Ministry of Education (grant no. RACER/1/2019/SKK08/UMS//1) and Universiti Malaysia Sabah (grant no. GUG0502-2/2020).

**References**

[1] World Health Organization (WHO), Global Tuberculosis Control Report 2020, 2020, 1-25.

[2] Chan H., et. al., Potential and use of bacterial small RNAs to combat drug resistance: a systematic review, Infect. Drug. Resist., 2017, 10:521-532.

[3] Ostrik A. et. al., Small noncoding RNAs and their role in the pathogenesis of *Mycobacterium tuberculosis* infection, Biochemistry, 2021, 86:109-119.

## P-11 Determining an optimal DNA isolation method for rodent fecal samples by 16S rDNA bacterial diversity identification

### Nik Amirah Auni Binti Nik Mohd Asri, Zarina Amin and Yew Chee Wei

#### Biotechnology Research Institute, Universiti Malaysia Sabah, Sabah, Malaysia

##### **Correspondence:** Nik Amirah Auni Binti Nik Mohd Asri (nikamirahauni@gmail.com)

From The International Conference on Molecular Diagnostics & Biomarker Discovery 2022 (MDBD 2022)

Penang, Malaysia. 11 – 13 October 2022

**Background**

The choice of an optimum DNA isolation method from fecal samples is a constant challenge as ineffective lyses of diverse bacterial cells may lead to bias detection in the representation of a bacterial community in a sample. In addition, degraded DNA and presence of PCR inhibitors such as humic acid and polysaccharides carried over from fecal samples have been known to further reduce PCR efficiency. The purpose of this study is to determine an optimal DNA extraction method for rodent samples by assessing the general bacterial diversity based on PCR products amplified from the partial 16S rDNA gene. The PCR-DGGE technique separates the identical size of PCR amplicons by their differential mobility on gel based on sequence variations.

**Methodology**

In this study, five (5) fecal samples from wild rodents were collected and added into the Zymo Research DNA/RNA Shield reagent as preservative. Six DNA extraction methods i.e. QIAamp PowerFecal Pro DNA Kit (QIAGEN), the QIAamp AllPrep PowerViral DNA/RNA Kit (QIAGEN), the QIAamp UCP Pathogen Kit (QIAGEN), the ZymoBIOMICS DNA Miniprep Kit (ZYMO Research), and two conventional methods using different component in lysis buffer which were guanidium thiocyanate and CTAB were tested using between 10 to 15 mg of feces. The samples were extracted according to manufacturer’s protocol and previous article [1] with some modifications, respectively. The extracted DNA were then subjected to amplification of V2 to V3 region of the bacterial 16S rDNA gene and DGGE separation of the amplicons. The DGGE banding pattern was analysed using the GelCompar II software (Applied Maths, Belgium) to compare the microbial diversity by using the Shannon-Weaver index [2].

**Results and Discussion**

The results showed that DNA yield varied with the extraction method; where the conventional method using guanidium thiocyanate showed a higher yield (average 324.22 ng/ul) than the other methods (average 45.33 ng/ul for the QIAamp PowerFecal Pro DNA Kit, 51.92 ng/ul for the QIAamp AllPrep PowerViral DNA/RNA Kit, 220.74 ng/ul for the QIAamp UCP Pathogen Kit, 42.4 ng/ul for ZymoBIOMICS DNA Miniprep Kit, and 165.74 ng/ul for the conventional method using CTAB). Majority of DNA extracted showed degradation when checked by gel electrophoresis. The QIAamp PowerFecal Pro DNA Kit was observed to show intact DNA. The DGGE profiles showed that the QIAamp PowerFecal Pro DNA Kit and QIAamp AllPrep PowerViral DNA/RNA Kit extracted the highest number of DNA bands (total of 168 and 167), while QIAamp UCP Pathogen Kit, ZymoBIOMICS DNA Miniprep Kit, and both conventional methods using guanidium thiocyanate and CTAB recorded 161, 160, 147, and 152 total of bands, respectively.

**Conclusion**

As a conclusion, QIAamp PowerFecal Pro DNA kit is the optimum DNA isolation method for rodent fecal samples as it provides better quality of DNA and microbial diversity in their DGGE profiles. Furthermore, this method is much time efficient QIAamp PowerFecal Pro DNA Kit and QIAamp AllPrep PowerViral DNA/RNA Kit produced more bands on their DGGE profiles than the other methos due to their use of bead-containing lysing matrix and vigorous homogenization.

**References**

[1] Chomczynski P, et al. The single-step method of RNA isolation by acid guanidium thiocyanate-phenol-chloroform extraction: twenty-something years on. Nature Protocols, 2006, 1:2:581-585.

[2] Boon N, et al. Evaluation of nested PCR-DGGE (denaturing gradient gel electrophoresis) with group-specific 16S rRNA primers for the analysis of bacterial communities from different wastewater treatment plants. FEMS Microbiology Ecology, 2002, 39:2:101-112.

## P-12 The toxic effects of *p*-Cresyl Sulfate on bone metabolism

### Ahmad Faizal Bin Jelani^1^, Gabrielle Ruth Anisah Fromming^2^, Dayang Erna Zulaikha Binti Awang Hamsin^3^ and Mohammad Zulkarnaen Bin Ahmad Narihan^1^

#### ^1^Department of Pathology, Faculty of Medicine and Health Sciences, Sarawak, Malaysia; ^2^Department of Basic Medical Sciences, Faculty of Medicine and Health Sciences, Sarawak, Malaysia; ^3^Department of Paraclinical Sciences, Faculty of Medicine and Health Sciences, Sarawak, Malaysia

##### **Correspondence:** Mohammad Zulkarnaen Bin Ahmad Narihan (anmzulkarnaen@unimas.my)

From The International Conference on Molecular Diagnostics & Biomarker Discovery 2022 (MDBD 2022)

Penang, Malaysia. 11 – 13 October 2022

**Background**

*p*-Cresyl Sulfate (*p*CS) is a uremic toxin that has been implicated in kidney disease, cardiovascular risks, endothelial dysfunction and neuropathies. In chronic kidney disease (CKD), *p*CS progressively accumulates in the body as the dysfunctional kidneys have a reduced ability to excrete toxins normally. *p*CS accumulated as a consequence of CKD cannot be removed from th body through dialysis, hence leading to further accumulation. This ultimately leads to bone loss which correlates with the worsening of CKD. As such, *p*CS could play a part in the development of bone loss with CKD. The objective of this review is to further understand the comprehensive effects pCS has on the bone.

**Methodology**

An extensive literature search was conducted in PubMed using the following keywords, ‘*p*-Cresyl Sulfate’ OR ‘*p*-Cresol Sulfate’ OR ‘*p*-Cresyl Sulphate’, ‘uremic toxin’ OR ‘uraemic toxin’ AND ‘bone’ OR ‘osteoblast’ OR ‘osteoclast’ OR ‘osteocyte’. From 2013 to 2022, 54 papers were found that contained the following keywords. Out of the 54 papers, 27 papers with significance were selected. The inclusion criteria for the study are in vivo and in vitro studies that examined the effects of *p*CS on bone. The exclusion criteria for the study is as follows: review article. After reviewing each article, 5 papers were selected for this review.

**Results and Discussion**

*p*CS is a prototype protein-bound uremic toxin associated with a multitude of toxic biological and biochemical effects. *p*CS is a substrate for human organic anion transporters (hOAT) 1 and 3 which is expressed in osteoblasts and other tissues. hOAT1 and hOAT3 may have a physiological role as large-capacity *p*CS transporters which could be a factor in the accumulation of *p*CS to toxic levels in osteoblasts [1]. Accumulation of *p*CS affects sclerostin production. As an inhibitor of the Wnt-signalling pathway, sclerostin prevents osteoblast formation and osteoprotegerin (OPG) production, inhibits osteoblast-mediated bone formation, and stimulates bone resorption by stimulating RANKL expression in osteocytes [2]. *p*CS also increase apoptosis and reduce osteoblast proliferation and viability with or without increased oxidative stress. This manifests by downregulation of the parathyroid hormone receptor (PTHR) on osteoblasts and a decrease in PTH-stimulated cAMP production through activation of c-Jun N-kinase (JNK) and p38 mitogen-activated protein kinase (MAPK) [3]. and triggers JNK phosphorylation by attenuating PTH responsiveness in the bones under CKD conditions.

**Conclusion**

This review evaluates the toxic effects *p*CS induces on bone. However, further studies must be conducted to understand the mechanism of action and full effects *p*CS has on bone metabolism. In the future, *p*CS could potentially become a new therapeutic target for the management of bone disorders.

**Acknowledgment:** The authors acknowledged the financial support from the Ministrv of Higher Education Malavsia through Fundamental Research Grant Scheme (FRGS) with grant number of FRGS/1/2021/SKKO/UNIMAS/01/2

**References**

[1] Watanabe, Hiroshi et al. “Human organic anion transporters function as a high-capacity transporter for p-cresyl sulfate, a uremic toxin.” *Clinical and experimental nephrology*vol. 18,5 (2014): 814-20. doi:10.1007/s10157-013-0902-9

[2] Desjardins, Lucie et al. “Uremic toxicity and sclerostin in chronic kidney disease patients.” *Nephrologie & therapeutique* vol. 10,6 (2014): 463-70. doi:10.1016/j.nephro.2014.04.002

[3] Tanaka, Hisae et al. “p-Cresyl sulfate induces osteoblast dysfunction through activating JNK and p38 MAPK pathways.” *Bone* vol. 56,2 (2013): 347-54. doi:10.1016/j.bone.2013.07.002

## P-13 Characterization of ssDNA aptamers against ACE2 protein as therapeutic targets for COVID-19 infection

### Zi Yuan Chang^1^, Falah Abbas Muhamad Salih^1, 2^ and Kai Ling Chin^1^

#### ^1^Department of Biomedical Sciences, Universiti Malaysia Sabah, Sabah, Malaysia; ^2^Department of Medical Laboratory Science, Cihan University, Erbil, Iraq

##### **Correspondence:** Kai Ling Chin (chinkl@ums.edu.my)

From The International Conference on Molecular Diagnostics & Biomarker Discovery 2022 (MDBD 2022)

Penang, Malaysia. 11 – 13 October 2022

**Background**

Coronavirus disease 2019 (COVID-19), a highly contagious and rapidly spreading disease with significant fatality in the elderly population has swept across the world for the past three years since 2019. The enormity scale of this pandemic has resulted in the emergence of several SARS-CoV-2 variants with Omicron, the current main circulating variant of concern [1]. Nonetheless, all the variants identified to date share the same cell entry mechanism where the process is initiated when the spike protein of the viruses attach to the angiotensin-converting enzyme 2 (ACE2) receptors on their host cells [2]. Therefore, blocking the spike protein-ACE2 interaction using a biological binder targeting the ACE2 is a viable strategy for COVID-19 treatment. Aptamer is a short length of nucleic acid [RNA or single-stranded DNA (ssDNA)] which is selected through an *in vitro* selection called systematic evolution of ligands by exponential enrichment (SELEX). The selected aptamers have a 3D conformation that can specifically bind to their target with high affinity and pose many superior properties such as being smaller in size, thermally stable, and nearly non-immunogenic.

**Methodology**

Single-stranded DNA library of 76-bp oligonucleotides containing a randomized core region of 40 nucleotides, flanked by primer binding regions of 18 nucleotides on each side (5’-ATACCAGCTTATTCAATT-N40-AGATAGTAAGTGCAATCT-3’) was synthesized. Recombinant His-tagged ACE2 protein (BBI Life Sciences, China) was immobilized to Ni-NTA Magnetic Beads (Gold Biotechnology, USA). Aptamers which showed high affinity towards the ACE2 were then selected from the initial library using SELEX. The isolated aptamers were cloned using the PCR Cloning Kit (NEB, USA) and the plasmids were extracted using DNA-spin Plasmid DNA Extraction Kit (iNtRON Biotechnology, Korea). The plasmids were sequenced and the resulting aptamer sequences were subjected to the UNAFOLD web server (http://www.unafold.org/) for ssDNA secondary structure prediction, followed by RNA tertiary structure prediction using RNAComposer (https://rnacomposer.cs.put.poznan.pl/), and finally, converted to equivalent ssDNA tertiary structure using Discovery Studio Visualizer v3.5. The aptamer-ACE2 interaction was predicted using the HDOCK docking program (http://hdock.phys.hust.edu.cn/).

**Results and Discussion**

Aptamers specifically bound to ACE2 were isolated after 13 rounds of magnetic bead-based SELEX. Here, we reported one of the isolated aptamers, Apt15, which potentially binds to the ACE2. The UNAFOLD revealed that this aptamer has a hairpin loop structure and single-stranded region with a Gibbs free energy value of -0.10. The low free energy value indicates that the ssDNA structure is thermodynamically stable [3]. *In silico* 3D molecular docking demonstrated that the single-stranded region of Apt15 binds to the ACE2 at the site recognized by the SARS-CoV-2 spike protein with a confidence score of 0.9894, thus suggesting the potential application of this aptamer as a therapeutic target for COVID-19 infection.

**Conclusion**

In conclusion, one ssDNA aptamer targeting ACE2 with therapeutic potential against COVID-19 was successfully identified in this study. Further investigations are necessary to determine its binding affinity to ACE2 and its ability to block the virus spike protein-ACE2 interaction. In future, this aptamer could serve as a broad-spectrum inhibitor against any existing or future emerging viruses that also use ACE2 for cell entry.

**Acknowledgement:** The work was funded by the Skim Dana Khas (SDK0226-2020) from the Universiti Malaysia Sabah.

**References**

[1] Parums DV. World Health Organization (WHO) Variant of Concern Lineages Under Monitoring (VOC-LUM) in response to the global spread of lineages and sublineages of omicron, or B.1.1.529, SARS-CoV-2. Med Sci Monit. 2022, 28: e937676-1.

[2] Han P, et al. Receptor binding and complex structures of human ACE2 to spike RBD from omicron and delta SARS-CoV-2. Cell. 2022, 185:630-640.

[3] Navien TN, et al. In silico molecular docking in DNA aptamer development. Biochimie. 2021, 180: 54-67.

## P-14 Detection of *Campylobacter jejuni* and *Campylobacter coli* from retails broiler chicken by duplex PCR

### Susilahwati Muhammad, Zaidah Abdul Rahman and Nur Syafikah Muhammad Nasir

#### Department of Medical Microbiology & Parasitology, School of Medical Sciences, Universiti Sains Malaysia, Kubang Kerian,16150 Kelantan, Malaysia

##### **Correspondence:** Zaidah Abdul Rahman (drzaidah@usm.my)

From The International Conference on Molecular Diagnostics & Biomarker Discovery 2022 (MDBD 2022)

Penang, Malaysia. 11 – 13 October 2022

**Background**

*Campylobacter* is one of the leading causes of foodborne diarrhoea illness in the developed countries and cause a significant public health concern worldwide. This study was designed to determine the prevalence of *Campylobacter coli and C.jejuni* contamination in poultry retails meat in Kota Bharu by duplex PCR from direct samples.

**Methodology**

A total of 50 fresh and chilled poultry chicken meat were purchased from 13 retail markets in. The samples were put into polyethylene bag and wash with Phosphate-buffered solution, the bacterial lysate was prepared directly from chicken wash, enriched in CCDA broth for 48 hours and the presence of *C.coli* and *C.jejuni* were detected by duplex PCR.

**Result and discussion**

Overall results revealed 22 % contamination of *Campylobacter* occur in poultry retail meat in Kota Bharu, Kelantan. Out of the fifty samples of fresh (n= 16) and frozen/chilled (n=34) chicken thigh, 10/50 (20%) was positive for *C.coli* and/or *C.jejuni*. Majority (9/10, 90%) of contamination were *C.coli* whereas, the remaining (1 sample) was *C. jejuni*. Frozen/chilled samples have lower frequency of *Campylobacter* contamination,11.8% (4/34) as compared to fresh samples, 37.5% (6/16). No *C.jejuni* contamination was observed in fresh samples, and only 2.9% (1/34) in frozen/chilled samples. While *C.coli* detected at rate 6/16(37.5%) in fresh chicken samples and 4/34(11.7%) in frozen/chilled chicken samples. The prevalence of *Campylobacter* spp in retail meat Kota Bharu was 22% (11/50) of fresh and frozen chicken meat is relatively low as compared to other result reported previously in Japan and Egypt who obtained retail level rate varying from 64-68%. Meanwhile, the reported prevalence of *Campylobacter* spp in broiler chicken in Malaysia even greater up to 97.1%, other studies done in East-cost Malaysia shows 20% of contamination from total of 120 cloaca swabs taken whereas the later obtain 45%-70%. The contamination attributed by the cross-contamination of poultry carcasses during defeathering, evisceration and carcass chillers, which reflected by the contamination rate of cases and meat. The result showed higher prevalence of contamination with *C.coli* and *C.jejuni* in fresh chicken samples compared to frozen/chilled. Previous studies showed the influence of chilled meat and inclusion of skin as determining factors in the prevalence of *Campylobacter* spp. Out of 34 chilled chicken samples, 11.8 % (4/34) were positive with *Campylobacter* spp, this 3-fold higher in fresh chicken samples, 37.5% (6/16). This in consistent with study done in central part of Malaysia, stated that apart from chilled temperature, packing in polyvinylidene film-overwrapped storiform trays may indirectly creating microaerophilic conditions which facilitate the survivability of *Campylobacter*.

**Conclusion**

The prevalence of *C.coli* and *C.jejuni* among poultry retails chicken meat in Kota Bharu, Kelantan is low as compared to limited data in other part of Malaysia. However, more studies with a larger sample sizes and involvement of retail market are required.

**Acknowledgement:** Special thanks to Universiti Sains Malaysia for the Geran Penyelidikan Sarjana Perubatan PPSP (GPSP) 1001/PPSP/8070011.

## P-15 The efficacy and safety of PD-1/PD-L1 immunotherapy in endometrial cancer: a meta-analysis

### Mohd Nazzary Mamat @ Yusof, Kah Teik Chew, Nirmala Kampan, Nor Haslinda Abd. Aziz and Mohamad Nasir Shafiee

#### Gynaecologic-Oncology Unit, Department of Obstetrics and Gynaecology, Universiti Kebangsaan Malaysia Medical Centre, Kuala Lumpur 56000, Malaysia

##### **Correspondence:** Kah Teik Chew (drchewkt@gmail.com)

From The International Conference on Molecular Diagnostics & Biomarker Discovery 2022 (MDBD 2022) Penang, Malaysia. 11 – 13 October 2022

**Background**

The programmed cell death protein 1 (PD-1)/Programmed cell death ligand 1 (PD-L1) pathway has a crucial role in the immune escape mechanism and growth of cancer cells in various cancer types, including endometrial cancer [1]. In exploring better treatment options, immune checkpoint inhibitor studies of PD-1/PD-L1 in endometrial cancer are actively conducted in clinical trials with promising findings in other cancers. However, the efficacy of those immunotherapy remains inconclusive, and guidelines are inconsistent [2]. Therefore, this meta-analysis aims to provide an updated and robust analysis of the effectiveness and safety of PD-1/PDL1 immunotherapy in endometrial cancer on the objective response rate (ORR), disease control rate (DCR), and adverse events.

**Methodology**

A literature search was conducted using the database of PubMed, Scopus, and Web of Science databases by using the keywords of “endometrial cancer” OR “PD-1 inhibitor” OR “PD-L1 inhibitor” OR “immunotherapy” OR “immune checkpoint inhibitor” OR “atezolizumab” OR “avelumab” OR “dostarlimab” OR “durvalumab” OR “nivolumab” OR “pembrolizumab”. Meta-analysis of proportion and association was pooled using STATA version17 and RevMan version 5.4 software, respectively. The effect of immunotherapy on outcome parameters was estimated by effect size (ES) or odds ratio (OR) with 95% confidence intervals (CIs) for each study.

**Results and Discussion**

Five studies between 2017 and 2022 met the inclusion criteria, with 480 endometrial cancer cases undergoing PD-1/PD-L1 immunotherapy clinical trials. The pooled proportion of ORR undergo PD-1/PDL-1 inhibitor treatment was 26.47% [95% CI = 16.68–37.52]. Subgroup meta-analysis showed the pooled ORR of the proficient mismatch repair (pMMR) group was 8.80% [95% CI = 0.20–24.39], and which was 37.03% [95% CI = 23.22–51.95] of the deficient mismatch repair (dMMR) group. Meta-analysis proportion of DCR undergo PD-1/PD-L1 inhibitor treatment was 41.44% [95% CI = 36.38–46.60]. Subgroup meta-analysis showed the pooled proportion DCR of the pMMR group was 24.97% [95% CI = 14.87–36.49] and dMMR group was 45.02% [95% CI = 39.26–50.85]. The efficacy of the PD-1/PD-L1 inhibitor showed a significantly higher ORR in dMMR than pMMR [OR = 6.30; 95% CI = 3.60–11.03]. Results were the same for DCR, a significantly higher in dMMR than pMMR [OR = 2.57; 95% CI = 1.66–3.99]. The safety pooled proportion of patients with at least one adverse event was 69.19% [95% CI = 62.56–75.46], and grade three or higher facing adverse events was 15.38% [95% CI = 12.01–19.04]. Based on the MSI status subgroup analysis of ORR and DCR, the efficacy of PD-1/PD-L1 immunotherapy in endometrial cancer was significantly better in dMMR patients.

**Conclusion**

PD-1/PD-L1 immunotherapy in endometrial cancer shows a promising outcome in early clinical trials, found to have better efficacy in the dMMR population. Thus, patients with dMMR are more suitable for this treatment. Further studies on targeted immunotherapy approaches need to be fully explored before it can be applied for a better treatment outcome.

**Acknowledgement:** The work was funded by the Fundamental Research Grant Scheme (FRGS/1/2020/SKK0/UKM/03/2) from the Ministry of Higher Education, Malaysia.

**References**

[1] Zhang C, Yang Q. Predictive Values of Programmed Cell Death-Ligand 1 Expression for Prognosis, Clinicopathological Factors, and Response to Programmed Cell Death-1/Programmed Cell Death-Ligand 1 Inhibitors in Patients with Gynecological Cancers: A Meta-Analysis. Front Oncol 2020; 10:572203.

[2] Maiorano BA, Maiorano MFP, Cormio G, Maglione A, Lorusso D, Maiello E. How Immunotherapy Modified the Therapeutic Scenario of Endometrial Cancer: A Systematic Review. Front Oncol 2022;12.

## P-17 Assessment of the NGS data using FastQC for quality control in the study of antiproliferative effects of dichloromethane *Clinacanthus nutans* fractions extracts on human breast cancer cell lines for biomarker discovery

### Zaleha Md Toha^1^, Nor Hasyimah Haron^1^, Kristine Sandra Pey Adum^1^, Nik Nur Syazni Nik Mohamed Kamal^1^, Melati Khairuddean^2^ and Hasni Arsad^1^

#### ^1^ Advance Medical and Dental Institute, Universiti Sains Malaysia, Bertam, Penang, Malaysia; ^2^ School of Chemistry, Universiti Sains Malaysia, Penang, Malaysia

##### **Correspondence:** Hasni Arsad (hasniarsad@usm.my)

From The International Conference on Molecular Diagnostics & Biomarker Discovery 2022 (MDBD 2022)

Penang, Malaysia. 11 – 13 October 2022

**Background**

In Malaysia, *Clinacanthus nutans* (CN) leaves are traditionally used as a treatment for breast cancer. However, scientific approval of the mode of action of the CN extract on MCF-7 cell lines at the molecular level is still lacking. High throughput Next-Generation Sequencing technology (NGS) to obtain large sequence numbers that benefit genetic medical research was used in this experiment [1, 2]. Bioinformatics tools such as FastQC and Picard were used to evaluate sequence data for quality control as an essential method during the analytical pipeline [3] that is important to identify challenges of systematic bias challenge in this study. The objective of this study was to evaluate the quality of NGS data sequence using FastQC in the early part of transcriptomic analysis pipelines of antiproliferative effects of dichloromethane CN fractions extracts on human breast cancer cell lines for biomarker discovery.

**Methodology**

Dichloromethane *CN* leaves extract with 50% inhibitory concentration (IC_50_) value of 108μg/mL was exposed to the human breast cancer cells, MCF-7 for 72hrs chosen from a previous study. RNA was extracted from the treated and untreated cells for transcriptomic sequence, NGS technology using the Illumina HiSeq4000 platform. The sequencing output was the raw data that must be removed from the adapter sequences by the short read trimmed using FastQC (Babraham Bioinformatics, UK). This analysis uses the Linux operating system, Ubuntu 18.0v and the adapter was removed by a barcode tool, FlexbarFlexbarth its command line and script. All results were presented in multiQC which aggregated the results and generated a single HTML report with plots to visualize and compare various metrics between the samples involved.

**Results and Discussion**

The output of multiQC was filed from FastQC. In the summary report of FastQC status, important attention was made based on sequence quality and sequence length distribution. After RNA sequencing using the paired ends modules samples generated GC overall percentage of 54% and a length of 150bp showing no difference in GC composition and no biased library complexity, differences in amplification, or library specific causes. The sequence quality resulted in a pass or good result in the green area and more than 30 phred scores for all samples. As a result, the total overrepresented sequences found in each library make up more than 0.1% of the total that was passed and good quality which does not affect the biological consideration. Overall, the quality of RNA-seq data was high and the sequence quality was also good.

**Conclusion**

Multi QC report using FastQC is an advantageous tool because it is relatively quick to generate and provides a clear method for comparing samples to determine consistency and identify problematic samples. From this study, the multiQC report contains high quality clean data that was downstream in all future transcriptomic analyses for biomarker discovery in breast cancer research.

**Acknowledgement:** The work was funded by the Fundamental Research Grant Scheme (FRGS/203/CIPPT/6711340) from the Ministry of Higher Education, Malaysia and USM Brifging Grant (304/CIPPT/6316239) to PM Dr Hasni Arsad.

**References**

[1] Taemook Kim, et al. Octopus-toolkit: a workflow to automate mining of public epigenomic and transcriptomic next-generation sequencing data. Nucleic Acid Research. 2018, 46:9.

[2] Guilherme de Sena Brandine and Andrew D.Smith. Falco: high-speed FastQC emulation for quality control of sequencing data. F1000Research. 2021, 8:1874.

[3] Christoper M.Ward, et al. ngsReports; an R package for managing FastQC report and other NGS related log files.bioRxiv. 2018.

## P-18 IGF-1 and IGFBP-1 expressions as the potential prognostic biomarkers in women with Endometrioid Endometrial Cancer (EEC)

### Abdul Muzhill Hannaan Abdul Hafizz^1, 2^, Reena Rahayu Md Zin^1^, Nor Haslinda Abd Aziz^2^, Muaatamarulain Mustangin^1^, Nirmala Chandralega Kampan^2^, Norfilza Mohd Mokthar^3^, Kah Teik Chew^2^ and Mohamad Nasir Shafiee^2^

#### ^1^Department of Pathology, Faculty of Medicine, Universiti Kebangsaan Malaysia Medical Centre, Kuala Lumpur, Malaysia; ^2^Department of Obstetrics and Gynaecology, Faculty of Medicine, Universiti Kebangsaan Malaysia Medical Centre, Kuala Lumpur, Malaysia; ^3^Department of Physiology, Faculty of Medicine, Universiti Kebangsaan Malaysia Medical Centre, Kuala Lumpur, Malaysia

##### **Correspondence:** Mohamad Nasir Shafiee (nasirshafiee@hotmail.com)

From The International Conference on Molecular Diagnostics & Biomarker Discovery 2022 (MDBD 2022)

Penang, Malaysia. 11 – 13 October 2022

**Background**

Insulin-like growth factor-1 (IGF-1) and the IGF binding protein 1 (IGFBP-1) expressions have been shown to play a vital role in cancer biology, including endometrioid endometrial cancer (EEC). We examined the prognostic value of these locally expressed biomarkers in endometrial biopsies and correlated them with clinicopathological data of EEC.

**Methodology**

mRNA expression of *IGF-1* and *IGFBP-1* in the endometrial biopsies were analysed in patients with EEC (n=25) and control (n=25) cases using quantitative polymerase chain reaction (qPCR) method. These data were then validated using immunohistochemistry (IHC) analysis and combined with EEC cases form a separate cohort (n=71) comprised of consecutive patients who underwent hysterectomy at UKMMC, between the year of 2014 to 2019. Overall survival was evaluated using the Kaplan-Meier method, with differences compared using the log- rank test. Independent relationships between these biomarkers and clinicopathological data were assessed using multivariate logistic regression models.

**Results and Discussion**

The *IGF-1* and *IGFBP-1* mRNA expressions were not significantly different between both groups, EEC vs. control. However, IGF-1 expression in IHC analysis was observed to be highly expressed in the EEC compared to the control group, while IGFBP-1 had a low expression in the EEC cases (*P*<0.05). IGF-1 was significantly associated with prognostic features ofEEC (*P*<0.05), while no associations were found in the IGFBP-1 expression. In our sub-analysis, high IGF-1 and negative IGFBP-1 expression were significantly correlated with poor progression-free survival (PFS) in advanced stage of EEC (*P*<0.05). Univariate and multivariate analyses showed that IGF-1 served as a predictive biomarker in EEC survival. Therefore, we postulate the continuous high expression of local IGF-1 protein in EEC cells could lead to poor outcomes. Our findings support that the circulating estrogen and IGF-1 were independently associated with a higher risk of recurrence in patients with stage III and IV of EC (Merritt et al. 2021). We also propose that a shift in the local expression of IGFBP-1 and IGF-1 may serve as a biomarker for the prognosis of EEC development.

**Conclusion**

Local expression of IGF-1 and IGFBP-1 may serve as prognostic biomarkers for EEC.

**Acknowledgement:** The work was funded by the Dana Fundamental PPUKM (FF-2019-492) from Universiti Kebangsaan Malaysia.

**Reference**

[1] Merritt, M. A., Strickler, H. D., Hutson, A. D., Einstein, M. H., Rohan, T. E., Xue, X., Sherman, M. E., Brinton, L. A., Yu, H. & Miller, D. S. 2021. Sex Hormones, Insulin, and Insulin-Like Growth Factors in Recurrence of High-Stage Endometrial Cancer. *Cancer Epidemiology and Prevention Biomarkers* 30(4): 719-726

## P-19 Pharmacological consequence of inhibiting MAPK p38 using Ralimetinib dimesylate on lipopolysaccharide-induced E-selectin and VCAM-1 expression in HUVEC

### Dayang Erna-Zulaikha

#### Department of Paraclinical Sciences, Universiti Malaysia Sarawak, 94300, Sarawak, Malaysia

##### **Correspondence:** Dayang Erna-Zulaikha (ahdezulaikha@unimas.my)

From The International Conference on Molecular Diagnostics & Biomarker Discovery 2022 (MDBD 2022)

Penang, Malaysia. 11 – 13 October 2022

**Background**

Endothelial dysfunction plays a prominent role in the pathogenesis of sepsis and is associated with life-threatening organ dysfunction. Lipopolysaccharide (LPS), a Gram-negative bacterial component, is an important sepsis-associated mediator that induces the expression of adhesion molecules such as E-selectin and VCAM-1 upon its binding to dedicated pattern recognition receptors on endothelial cells (EC) [1]. Endothelial E-selectin and VCAM-1 expression promotes leukocyte adhesion, and high leukocyte infiltration in various organs is associated with a poor prognosis in sepsis patients. As the expression of E-selectin and VCAM-1 is partly driven by the activation of the MAPK p38 signaling pathway [2], it is unknown whether Ralimetinib dimesylate (RM), a selective p38 MAPK inhibitor, can be used as a treatment to reduce E-selectin and VCAM-1 expression once LPS-driven activation of EC has started. RM treatment was previously shown to reduce TNF-α production in LPS-induced macrophages *in vitro* [3]. In this study, I investigated the pharmacological effect of RM treatment on E-selectin and VCAM-1 expression in LPS-stimulated HUVEC was investigated.

**Methodology**

Ten μM of RM was added into the HUVEC medium at 0.5, 1, 1.5, and 2 hours after HUVEC was exposed to 1 μg/ml of LPS. After 2, 4, 5, and 6 hours of LPS exposure, the cells were trypsinized and subjected to flow cytometry analyses. The Mean Fluorescence Intensity (MFI) of E-selectin and VCAM-1- conjugated fluorochromes were determined in the treatment groups and statistically compared to LPS-stimulated HUVEC controls using one-way ANOVA and Bonferroni post-hoc test. Each group was represented by three biological replicates. The results were reported as mean + SD. Viability of HUVEC was monitored microscopically.

**Results and Discussion**

LPS induced the expression of E-selectin and VCAM-1 in HUVEC in a time-dependent manner. E-selectin and VCAM-1 were maximally expressed on HUVEC at 4 and 6 hours after LPS exposure, respectively. E-selectin expression was attenuated throughout the 6 hours’ duration of LPS exposure upon post-LPS treatment with RM at 0.5, 1, 1.5, and 2 hours after LPS exposure. VCAM-1 expression was not affected upon post-LPS treatments with RM. These findings suggest that MAPK p38 to be an important signaling cascade mediating LPS-induced E-selectin expression and can be pharmacologically targeted to attenuate E-selectin, but not VCAM-1 expression.

**Conclusion**

RM can be used to pharmacologically target the p38 MAPK signaling pathway to attenuate the expression of E-selectin, but not VCAM-1, in LPS-activated EC. Follow-up *in vivo* study should be done to investigate the effect of RM on the expression of E-selectin and VCAM-1 in animal model of experimental sepsis.

**References**

[1] J. Moser et al. Intracellular RIG-I Signaling Regulates TLR4-Independent Endothelial Inflammatory Responses to Endotoxin. J. Immunol., 2016; 196: 4681–4691.

[2] E.-Z. Dayang et al. Identification of LPS-Activated Endothelial Subpopulations With Distinct Inflammatory Phenotypes and Regulatory Signaling Mechanisms. Front. Immunol., 2019; 10: 1–12.

[3] Y. Yang et al*.* Functional Roles of p38 Mitogen-Activated Protein Kinase in Macrophage-Mediated Inflammatory Responses. Mediators Inflamm., 2014; 1–13.

## P-20 AST, ALT, Bilirubin and AST/ALT Ratio Role; COVID-19 Patients

### Nor Amirah Mohammad Nazri ^1^, Wan Norlina Wan Azman^1^, Norsyuhadah Musa^1^, Tuan Salwani Tuan Ismail^1^, Azian Harun^2^, Najib Majdi Yaacob^3^, Sarina Sulong^4^, Sirajudeen K.N.S^5^, Mahaya Che Mat^6^ and Hani Ajrina Zulkeflee^7^

#### ^1^ Department of Chemical Pathology, School of Medical Sciences, Universiti Sains Malaysia (Health Campus) Kelantan, Malaysia; ^2^ Department of Medical Microbiology and Parasitology, Universiti Sains Malaysia (Health Campus) Kelantan, Malaysia; ^3^ Unit of Biostatistic and Research Methodology, Universiti Sains Malaysia (Health Campus) Kelantan, Malaysia; ^4^ Human Genome Centre, Universiti Sains Malaysia (Health Campus) Kelantan, Malaysia; ^5^ Department of Basic Medical, Kuliyyah of Medicine, International Islamic University Malaysia, Kuantan Campus, Malaysia; ^6^ Department of Pathology, Hospital Raja Perempuan Zainab II, Kelantan, Malaysia; ^7^ Department of Medical Sciences II, Faculty of Medicine and Health Sciences, Universiti Sains Islam Malaysia

##### **Correspondence:** Nor Amirah Mohammad Nazri (ciknoramirah@gmail.com)

From The International Conference on Molecular Diagnostics & Biomarker Discovery 2022 (MDBD 2022)

Penang, Malaysia. 11 – 13 October 2022

**Background**

Impaired liver function upon admission has been linked to the severity of COVID-19 infection, yet the data is debated [1]. Therefore, this retrospective study aimed to evaluate the liver function among COVID-19 patients during hospitalization and its association with the disease severity.

**Methodology**

Patients aged 18 to 80 years with positive COVID-19 at Hospital Raja Perempuan Zainab II (HRPZ II), Kota Bharu, Kelantan, with available AST, ALT, Bilirubin, and AST/ALT ratio data on admission, were retrospectively evaluated from March 2021 until March 2022. Disease severity was categorized based on the Annex 2e guidelines by Ministry of Health Malaysia, which further classified them into mild to moderate disease (Stage 1-3) and severe to critical illness (Stage 4-5). The AST, ALT, Bilirubin, and AST/ALT ratio levels on Day 1 admission were archived from the electronic medical record system and compared between the two groups. Statistical analysis was performed using SPSS version 27. This study was approved by (JEPeM-USM) with protocol code USM/JEPeM/21100691 and the Ministry of Health Malaysia NMRR-21-762-58458 (IIR).

**Results and Discussion**

The study involved a total of 168 COVID-19 patients with a mean (SD) age of 46.67(16.10) for mild to moderate and 56.66(12.41) for severe to critical. There was a significant age group for both groups (p-value=0.002). During hospitalization, 16(14.41%) patients progressed to death from severe to critically ill patients. Upon admission, the median (IQR) of AST and ALT were significantly higher in the severe to critical group compared to the mild to moderate group, [AST; 39.0(49.0) and 24.0(14.0), ALT 38.0(43.0) and 21.0(18.0)], p<0.05. However, there were no significant differences between both groups for bilirubin level and AST/ALT ratio. Non-survivors had higher AST and ALT levels compared to survivors, with median (IQR) of [AST 98.0(88.0) and 32.0 (26.0), ALT of 67.5(90.0) and 28.0(31.0), (p<0.05). Similarly, there were no significant differences between non-survivors and survivors for bilirubin and AST/ALT ratio. Our study supports the idea that abnormal liver function at admission has been shown to be associated with the disease severity and mortality of COVID-19 infection. Therefore, there is a need to observe hepatobiliary sequelae in COVID-19 survivors as there are dynamic changes in liver function following hospital discharge.

**Conclusion**

Abnormal AST and ALT level at admission has been shown to be associated with the disease severity and mortality of COVID-19 infection. Further study needed to evaluate liver damage in COVID-19 post-discharge.

**Acknowledgements:** This study is funded by the Ministry of Higher Education Malaysia for the Fundamental Research Grant Scheme with project code: FRGS/1/2021/SKK06/USM/03/6 and External Agencies UTAS MAJU SDN BHD, 304.PPSP.6150238.U167.

**Reference**

[1] Liao X, Li D, Ma Z, Zhang L, Zheng B, Li Z, Li G, Liu L, Zhang Z. 12-Month Post-Discharge Liver Function Test Abnormalities Among Patients With COVID-19: A Single-Center Prospective Cohort Study. Frontiers in Cellular and Infection Microbiology. 2022:431.

## P-21 MALDI-TOF mass spectrometry-based strategy in discovery reproducible *N*-glycans in human fibroblast

### Salina Abdul Rahman, Affandi Omar, Nur Jannaim Muhamad and Julaina Abdul Jalil

#### Inborn Errors of Metabolism and Genetics Unit, Nutrition, Metabolism and Cardiovascular Research Centre, Institute for Medical Research, National Institutes of Health, Ministry of Health Malaysia, Bandar Setia Alam, 40170 Shah Alam, Selangor, Malaysia

##### **Correspondence:** Salina Abdul Rahman (sar@moh.gov.my)

From The International Conference on Molecular Diagnostics & Biomarker Discovery 2022 (MDBD 2022)

Penang, Malaysia. 11 – 13 October 2022

**Background**

Mass spectrometry (MS) has evolved over the years into a powerful tool that facilitates the identification of glycoproteins and *N*-glycans. *N*-glycans are essential for controlling metabolic pathways and cellular communication between cells, tissues, and organs. Because of this, there is a lot of interest in the study of glycosylation and *N*-glycans as biomarkers for several diseases [1]. Thus, the reproducible release and identification of *N*-glycans from tissue are important criteria for biomarker discovery. The aim of this study is to explore a general out-look on *N*-glycans reproducibility in fibroblast using matrix assisted laser desorption ionization time-of-flight (MALDI/TOF) mass spectrometry.

**Methodology**

Two different batches (batch 1 and 2) of the human fibroblast cell lines were grown to confluence subjected to FANGS protocol [2] and permethylated before mass spectrometry analysis. Sample from these two batches (Batch 1: A1, B1 and C1; Batch 2: A2, B2 and C2) were processed on different days and the released permethylated glycans were analyzed on different days using MALDI/TOF. Triplicate spots from each sample were spotted on a MALDI plate and analyzed three times. The resulting [M+Na] ^+^ glycan intensities from the spectra corresponding to the same sample were averaged and used to prepare bar charts.

**Results and Discussion**

Results showed that there were no significant differences between different spots of the same sample in all samples (*p*>0.05). This finding is similar to the recently published paper [3], in which the plasma *N*-glycan analysis of control samples was used to evaluate the reproducibility of sample processing. Reproducibility of sample processing on different days showed a few sialylated glycans were missing in A2 and B2 compared to A1 and B1 samples. This is possibly due to the low levels of the collected sialylated glycans thatmay be lost during sample processing or possibly due to biological variation during cell growth. There were differences in the signal intensities of the Hex5HexNAc2 and NeuAcFucHex5HexNAc4 species between the two batches. Most of the signal intensities are similar in the two batches prepared from the C cells.

**Conclusion**

In conclusion, this technique of *N*-glycan identification from fibroblast using mass spectrometry can be applied in investigations using human fibroblasts. Further features must be improved such as to reduce the variability of *N*-glycans obtained during sample processing or cell growth.

**References**

[1] Haan, et al. Clin Mass Spectrometry. 2020; 18:1-12.

[2] Abdul Rahman, et al, J Proteome Res. 2014;13(3):1167-76.

[3] Guillard et al. Clin Chem. 2011; 57(4), 593–602.

## P-22 Spray-dried andrographolide nanoparticles for the treatment of Alzheimer’s disease: Preparation and cytotoxicity evaluation

### Subashini Raman^1^, Vikneswaran Murugaiyah^1, 2^ and Thaigarajan Parumasivam ^1^

#### ^1^ School of Pharmaceutical Sciences, Universiti Sains Malaysia, 11800 Malaysia; ^2^ Centre for Drug Research, Universiti Sains Malaysia, 11800, Penang, Malaysia

##### **Correspondence:** Thaigarajan Parumasivam (thaigarp@usm.my)

From The International Conference on Molecular Diagnostics & Biomarker Discovery 2022 (MDBD 2022)

Penang, Malaysia. 11 – 13 October 2022

**Background**

Andrographolide is a major bioactive constituent found in *Andrographis paniculate* (Hempedu bumi in Malay). It has been reported to possess a promising neuroprotective effect that has the potential for the treatment of Alzheimer’s disease [1]. However, the penetration of andrographolide into the central nervous system via the blood-brain barrier is one of the main obstacles for the treatment of Alzheimer’s disease. Oral administration of *A. paniculata* extract has been reported to limit the reach of andrographolide to the brain owing to high plasma protein binding (55%) [2-3]. Andrographolide also readily degraded in acidic and alkaline environments of the gastrointestinal tract. Hence, it is hypothesised that if andrographolide in the nanoparticle form is delivered to the brain, the poor bioavailability limitations could be overcome. The current study aimed to produce spray-dried andrographolide nanoparticles and investigate their cytotoxicity in vitro using a human neuroblastoma cell line.

**Methodology**

Andrographolide nanoparticles were produced using a nano-spray dryer (Buchi Nano Spray Dryer B-90 HP). The particle size was measured using laser diffraction technology and electron microscopy was used to observe the morphology of the particles. The cytotoxicity of the nanoparticles was tested on SH-SY5Y neuroblastoma cells. The nanoparticle suspension at various concentrations was added into the cells in two-fold dilutions and incubated for 48 hrs. 0.05% resazurin was then added and further incubated for 4 hrs. Fluorescence readings were taken at a wavelength of 590nm following excitation at 544nm using a microplate reader. The half-maximal inhibitory concentration (IC_50_) was determined by comparing the inhibition to the untreated cells. In addition, lactate dehydrogenase (LDH) assay, nitrite oxide (NO) level and reactive oxygen species (ROS) level were determined using the supernatant of the cells following the assay kit guidelines. An andrographolide solution was included as a control. Three independent experiments were performed in triplicate.

**Results and Discussion**

The spray-dried andrographolide nanoparticles were spherical with a rough surface with a hydrodynamic diameter of 724 ± 39 nm with a polydispersity index of 0.3. The spray-dried nanoparticles showed lower toxicity (IC50 of 15.22 ± 0.61 μg/ml) as compared to the solubilised andrographolide control (IC50 of 10.82 ± 0.61 μg/ml). As anticipated, cells treated with solubilised andrographolide showed a higher amount of LDH release compared to the formulated nanoparticles, indicating that the formulation slightly reduced the toxicity of the andrographolide. The nitrate level in the supernatant did not increase dramatically compared to the untreated control, indicating that the cellular death did not involve the release of NO level in the cells. Similarly, the cells did not show any differences in ROS between the treated and untreated cells, suggesting that cellular death might not be induced through the ROS pathway.

**Conclusion**

These findings warrant further in vivo studies to establish the efficacy of the spray-dried andrographolide nanoparticles for the treatment of Alzheimer disease.

**Acknowledgement:** The work was funded by the Technology Development fund 1 (TDF03211038) from the Ministry of Science, Technology and Innovation, Malaysia.

**References**

[1] Patel et al. Protective effect of andrographolide against STZ induced Alzheimer’s disease in experimental rats: possible neuromodulation and Aβ((1-42)) analysis. Inflammopharmacology; 2021, 29: 1157-1168.

[2] Bera et al. Pharmacokinetic analysis and tissue distribution of andrographolide in rat by a validated LC-MS/MS method. Pharma Biol; 2014, 52: 321-329.

[3] Panossian et al. Pharmacokinetic and oral bioavailability of andrographolide from *Andrographis paniculata* fixed combination Kan Jang in rats and human. Phytomedicine; 2000, 7: 351-364.

## P-23 Cross-reactivity between Leptospira Serovars in microscopic agglutination test for Leptospirosis

### Nur Nadia Jamil, Murnihayati Hassan, Fairuz Amran, Rohaidah Hashim, Anira Abdul Hadi and Noor Hadirah Wahid

#### Infectious Disease Research Centre, Institute for Medical Research (IMR), Shah Alam, Malaysia

##### **Correspondence:** Nur Nadia Jamil (nurnadiajamil@gmail.com)

From The International Conference on Molecular Diagnostics & Biomarker Discovery 2022 (MDBD 2022)

Penang, Malaysia. 11 – 13 October 2022

**Background**

Leptospirosis is a zoonotic disease resulting in high morbidity and mortality in Malaysia and other endemic countries. Microscopic agglutination test (MAT) is the gold standard method in diagnostic leptospira armamentarium. MAT is a laborious serological test performed by observing agglutination reaction of patient sera when incubated against a panel of live Leptospira serovars. This study aimed to analyze the degree of cross-reactivity of sera for MAT conducted in Institute for Medical Research (IMR).

**Methodology**

The study included all sera for MAT in 2019. MAT was performed using an extended WHO panel comprising 20 serovars and 4 strains of *Leptospira species.* A titer of more than or equal to 1:400 is considered seropositive. Data analysis for serological cross reactivity was performed using Microsoft Excel for descriptive statistics.

**Results and Discussion**

96 sera (2.4%) out of 3965 sera were tested positive for MAT. Single seropositivity was observed in 40.6% of the sera and the remaining demonstrated cross-reactivity ranging from 2 to 9 serovars (average of 3 serovars). The high degree of cross reactivity observed may be a unique interplay between acute febrile illness (AFI) sera reacted to an extended MAT panel. Single serovar seropositivity involving serovar Fugis, Whitcombi and Lai Langkawi, *L. meyeri, L. borgpetersenii* serovar Batavae, may reflect a convalescent serum reacting to specific infecting serovar. Our finding is in parallel with other studies that support the optimization of extended MAT panels to suit locally dynamic circulating serovar. The limited number of paired sera in this study hinders the optimal determination of important infecting serovars.

**Conclusion**

An extended MAT panel tailored for local endemic serovar displays a high degree of cross-reactivity in AFI sera that serves as a more sensitive tool for the diagnosis of leptospirosis.

**Acknowledgements:** This work was funded by the Ministry of Health, Malaysia

**References**

[1] Siti Khairani Bejo, et al. Manual for Laboratory Diagnosis of Leptospirosis: One Health approach. 2017; p. 7-20

[2] Chintana Chirathaworn, et al. Interpretation of microscopic agglutination test for leptospirosis diagnosis and seroprevalence. Asian Pac. J. Trop. Biomed. 2014, 4:S162-164.

[3] Jayasundra D, et al. Optimizing the microscopic agglutination test (MAT) panel for the diagnosis of Leptospirosis in a low resource, hyperendemic setting with varied microgeographic variation in reactivity. PLoS Negl Trop Dis. 2021, 15.

## P-24 Susceptibility of human lung-derived cell lines MRC-5 and A549 to Malaysian SARS-CoV-2 isolate

### Siti Nur Zawani Rosli, Sitti Rahmawati Dimeng, Farah Shamsuddin, Mohammad Ridhuan Mohd Ali, Nur Afrina Muhamad Hendri, Hana Farizah Zamri, Jeyanthi Suppiah, Rozainanee Mohd Zain, Ravindran Thayan and Norazah Ahmad

#### Infectious Disease Research Center (IDRC), Institute for Medical Research (IMR), National Institutes of Health (NIH), Ministry of Health Malaysia (MOH)

##### **Correspondence:** Siti Nur Zawani Rosli (sn.zawani@moh.gov.my)

From The International Conference on Molecular Diagnostics & Biomarker Discovery 2022 (MDBD 2022)

Penang, Malaysia. 11 – 13 October 2022

**Background**

Severe Acute Respiratory Disease 2 (SARS-CoV-2) has been identified as the causal factor to the recent COVID-19 pandemic [1]. One of the capacities required to study this virus is to isolate and propagate them according to the requirement of the studies. To date, the majority of studies utilized monkey-derived Vero cells to rapidly propagate the virus. However, due to its highly susceptibility to SARS-CoV-2 infection and limited capacity to metabolize drugs, this cell line is not suitable for pathophysiology and anti-viral research. Vero cells are not preferred for investigation of pathological mechanism of host cell’s response to virus infection because they are not derived from human lung tissue. Furthermore, they are difficult to be used in the study to assess cytopathic effects as they do not express type 1 interferon genes [2]. In this study, we adapted the SARS-CoV-2 isolates into selected human lung-derived cell lines, i.e., MRC-5 and A549, to investigate the capacity of these cell lines as a platform for the characterization of SARS-CoV-2 isolated in Malaysia. MRC-5 has already been identified to be highly susceptible to the infection of various human coronaviruses, including HCoV-OC43, HCoV-229E and Middle East respiratory syndrome coronavirus (MERS-CoV) [3]. Meanwhile, A549 was selected due to its origin of lung derived.

**Methodology**

To determine the growth profile of SARS-CoV-2 in human-derived cell lines, a local SARS-CoV-2 clinical isolate obtained from the IMR archive was first adapted into Vero E6, thus resulting in the generation of passage 1 (P1). The P1 isolate was used to study the growth profile of the virus in MRC-5 and A549 cell lines. P1 isolate was subjected to Whole Genome Sequencing (WGS) to identify the genomic sequence of the isolate. The replication kinetics of these isolates in MRC-5 and A459 were evaluated based on the on the formation of cytopathic effect (CPE), Cq value, plaque forming unit (pfu) as well as observation by electron microscope (EM). Whole Genome Sequencing (WGS) was repeated on the propagated SARS-CoV-2 passages to examine the genomic stability.

**Results and Discussion**

There was no formation of CPE observed after inoculation of SARS-CoV-2 into MRC-5 and A549 cells after 7 days of culture. In parallel, the qRT-PCR results suggested that the virus did not multiply well in these cells. Plaque assay and EM images further supported these findings. In terms of genome stability, WGS data revealed several genetic polymorphisms in the genome of the virus adapted in MRC-5 and A549 cells.

**Conclusion**

MRC-5 and A549 are not susceptible to SARS-CoV-2 infection.

**Acknowledgements:** The work was funded by the NIH Research Grant NMRR-20-709-54523 from the Ministry of Health, Malaysia.

**References**

[1] MacKenzie JS, Smith DW. COVID-19: a novel zoonotic disease caused by a coronavirus from China: what we know and what we don’t. Microbiol Aust. 2020;41(1):45-50. doi:10.1071/MA20013

[2] Konishi K, Yamaji T, Sakuma C, et al. Whole-Genome Sequencing of Vero E6 (VERO C1008) and Comparative Analysis of Four Vero Cell Sublines. Front Genet. 2022;13:276. doi:10.3389/FGENE.2022.801382/BIBTEX

[3] Shirato K, Kawase M, Matsuyama S. Middle East Respiratory Syndrome Coronavirus Infection Mediated by the Transmembrane Serine Protease TMPRSS2. J Virol. 2013;87(23):12552-12561. doi:10.1128/JVI.01890-13/ASSET/915812EE-6A2E-404B-B80E-6370BB66EEF8/ASSETS/GRAPHIC/ZJV9990983100010.JPEG

## P-27 Effects of kaempferol on cell morphology, migration and microtubule functions in colorectal cancer cell HT-29

### Nurhuda Mohamad Ansor^1^, Fan Xu^1^, Nozlena Abdul Samad^1^ and Norfarazieda Hassan^2^

#### ^1^Department of Toxicology, Advanced Medical and Dental Institute, Universiti Sains Malaysia, 13200 Kepala Batas, Penang, Malaysia; ^2^Department of Biomedical Science, Advanced Medical and Dental Institute, Universiti Sains Malaysia, 13200 Kepala Batas, Penang, Malaysia

##### **Correspondence:** Nurhuda Mohamad Ansor (nurhuda.ansor@usm.my)

From The International Conference on Molecular Diagnostics & Biomarker Discovery 2022 (MDBD 2022)

Penang, Malaysia. 11 – 13 October 2022

**Background**

Kaempferol, a naturally-occurring flavonoid, exhibits similar structure to estrogen and exerts estrogen-like activity. Extensive studies have been done to investigate its anticancer properties against various cancers [1,2]. Here we report effects of kaempferol on cellular activities that are closely regulated by microtubules. We also reveal our finding on expression of main microtubules components following kaempferol treatment in colorectal cancer cell line HT-29.

**Methodology**

We carried out cell viability assay to examine whether kaempferol inhibits HT-29 cell growth by estimating viable cell numbers. Next, we performed cell spreading and scratch wound assay to investigate the effects of kaempferol on HT-29 cell morphology and migration activity. To obtain a clearer understanding of how kaempferol causes alterations in microtubule network and cell motility, we performed whole cell microtubule analysis by flow cytometry.

**Results and Discussion**

Kaempferol decreased viable HT-29 cell numbers in a dose-dependent manner with almost 66% decrease in the viable cell number within 24 hours after the addition of 240 μM kaempferol. During cell spreading, HT-29 cells treated with IC_50_ of kaempferol (143 μM) were found to be significantly reduced in cell area and increased circularity. This suggests kaempferol inhibits cell spreading and promotes a more circular-shaped cells. Further investigation on microtubule organization revealed that kaempferol suppressed normal microtubule spreading in HT-29 cells, consistent with reduction in the cell area. Kaempferol-treated cells were having intense anti-tubulin staining around the cell edge almost identical to the effects seen in taxol-treated HT-29 cells. Taxol treatment resulted in a more intense anti-tubulin staining, in agreement with its well-known role in promoting microtubule stabilization. More detailed microtubule analysis revealed that expression of α-tubulin and β-tubulin in kaempferol-treated cells was significantly lesser than untreated cells. Given the important roles of microtubule cytoskeleton in controlling cellular shape and cell motility, altered cell morphology and inhibition of cell migration found in kaempferol-treated HT-29 cells could be attributed to perturbation in microtubule functions.

**Conclusion**

Collectively, our findings show that kaempferol leads to alteration in cell spreading and hinders cell migration of cancer cells. These changes could be associated with decrease expression of α-tubulin and β-tubulin. Further work will be required to elucidate underlying mechanisms of kaempferol effects on microtubule dynamics.

**Acknowledgement:** Special thanks to Universiti Sains Malaysia for the financial assistance through Short Term Grant (304/CIPPT/6315481).

**References**

[1] Wang X, et al. The mechanism of anticancer action and potential clinical use of kaempferol in the treatment of breast cancer. Biomed Pharmacother. 2019, 117:109086.

[2] Wang F, et al. Kaempferol induces ROS-dependent apoptosis in pancreatic cancer cells via TGM2-mediated Akt/mTOR signaling. BMC Cancer. 2021, 21(1):396.

## P-28 Genomic characterization of the first case of extensively drug-resistant, travel-related *Salmonella enterica* Serovar Typhi in Malaysia

### Nor Hazrin Abd Hazis and Rohaidah Hashim

#### Institute for Medical Research (IMR), National Institute of Health (NIH), Ministry of Health (MOH), Selangor, Malaysia

##### **Correspondence:** Nor Hazrin Abd Hazis (norhazrin@moh.gov.my)

From The International Conference on Molecular Diagnostics & Biomarker Discovery 2022 (MDBD 2022)

Penang, Malaysia. 11 – 13 October 2022

**Background**

Typhoid fever, caused by *Salmonella enterica* serovar Typhi (*S*. Typhi) remains a serious global health concern. The effectiveness of antimicrobial therapy has been threatened by the emergence of Extensively Drug-resistant (XDR) *S*. Typhi exhibiting resistance to the first line and second-line antibiotic options. The aim of this study was to characterize an XDR *S*. Typhi isolate from a foreign worker with a history of recent travelling to Pakistan.

**Methodology**

Whole-genome sequencing (WGS) was performed on the isolate with Illumina MiSeq and data was analysed using Resfinder tool to detect the presence of genes associated with antimicrobial resistant profile. Pathogenwatch was used to characterize the genomic clonality in relation to the XDR outbreak of *S*. Typhi.

**Results and Discussion**

WGS analysis detected 10 antimicrobial resistance genes including aminoglycoside resistant gene (*aph(3”)-Ib, aph(6)-Id*, *aac(6’)-Iaa*), trimethoprim resistant gene (*dfrA7*), sulphonamide resistant gene (*sul1, sul2*), beta-lactam resistant gene (*blaCTX-M-15*, *blaTEM-1B*), phenicol resistant gene (*catA1*) and quinolone resistant gene (*qnrS*). Similar gene profiles were seen for the XDR *S*. Typhi from Pakistan. A core genome phylogeny tree constructed using selected genomes from Pathogenwatch collection clustered Malaysia XDR *S*. Typhi isolate with XDR isolates from Pakistan, United Kingdom and Italy.

**Conclusion**

This first case of typhoid fever due to XDR *S*. Typhi detected in Malaysia highlights the need to be vigilant for future cases. Genomic characterization of *S*. Typhi using WGS as a tool in surveillance program is important to manage outbreak investigation by studying the pathogen population structure, identification and tracking of potential source both local as well as global.

**Acknowledgements:** This work was funded by the Ministry of Health, Malaysia

## P-29 Suggestive SNPs of dengue pathogenesis towards severity in single-centre case-control study

### Norfarhana Khairul Fahmy^1^, Tengku Nurainna Fatihah Tengku Abdullah^2^, Jamiila Ismail^1^, Ching Yee Ming^2^, Nurhanani Md Nor^2^, Erina Faizati Kadri^2^, Koay Bee Tee^1^, Muhammad Zhafri Md Zakariah^1^, Masita Arip^3^ and Norhazlin Mustafa^1^

#### ^1^Transplantation Immunology Unit, Allergy & Immunology Research Centre, Institute for Medical Research (IMR), National Institutes of Health Malaysia (NIH), Ministry of Health Malaysia, Selangor, Malaysia; ^2^Autoimmune Unit, Allergy & Immunology Research Centre, Institute for Medical Research (IMR), National Institutes of Health Malaysia (NIH), Ministry of Health, Selangor, Malaysia; ^3^Allergy & Immunology Research Centre, Institute for Medical Research (IMR), National Institutes of Health Malaysia (NIH), Ministry of Health, Selangor, Malaysia

##### **Correspondence:** Norfarhana Khairul Fahmy (norfarhana.kf@moh.gov.my)

From The International Conference on Molecular Diagnostics & Biomarker Discovery 2022 (MDBD 2022)

Penang, Malaysia. 11 – 13 October 2022

**Background**

Dengue has persistently served as a major public health concern especially in the tropical and sub-tropical areas. In Malaysia itself, dengue is a hyperendemic disease and the incidence rate continued to increase exponentially since the past ten years [1]. Manifestations of the dengue symptoms can range from asymptomatic, mild or severe. Dengue fever that progresses to severe dengue, either dengue haemorrhagic fever (DHF) or dengue shock syndrome (DSS), are usually life-threatening. The mechanisms of how this disease progresses to severity is incompletely understood. Early identification of cases that are likely to progress to worse health conditions remains challenging. Multiple studies have highlighted the role of host genetics as a crucial factor towards severity. Thus, this study aimed to identify single nucleotide polymorphism(s) that predisposes dengue patients to severe disease outcomes among the Malaysian dengue cohort.

**Methodology**

This is a case-control study that includes a total of 188 dengue patients; 86 with dengue fever and 102 with severe dengue. Patients were recruited from Hospital Kuala Lumpur from year 2018 till 2020. Classification into dengue fever (DF) or severe dengue (SD) were made based on the WHO 2009 classification. DNA extracted from whole blood samples, were genotyped using Infinium™ Asian Screening Array. Single variant association analysis was performed on 617,245 single nucleotide polymorphisms (SNPs). Both sample- and SNP-levels data were subjected to stringent quality control checking for high quality genotype data.

**Results and Discussion**

We found genetic variations that are suggestively associated with dengue severity, namely rs9872672 and rs148681490. These SNPs reached the p-values of ~10^-6^, with their odds ratio of 4.574 (rs9872672) and 0.092 (rs148681490) were mapped to chromosome 3p14.2 and 11p11.2, respectively. Our findings at position 3p14.2 indicate a change of allele from C to T that occurred at a frequency of 0.143 globally [2]. Suggestive allele at this position identified an intronic region of the synaptoporin (SYNPR) gene that is predicted to be an integral component of synaptic vesicle membrane, whose probable function is linked with vesicular channel protein. Likewise, suggestive SNP found at chromosome 11 showed a change from A to G. The alleles at 11p11.2 encode for tetratricopeptide repeat domain 17 (TTC17) protein that are involved in the actin filament polymerization and cilium organization.

**Conclusion**

In conclusion, the findings from this study support the notion that host genetic factor contributes to the disease pathogenicity of dengue severity. Although the potential roles of the suggestive SNPs are still uncertain, further validation is highly warranted.

**Acknowledgement:** This work was funded by the Ministry of Health, Malaysia for a project coded NMRR-18-263-40377.

**References**

[1] Ministry of Health Malaysia. Clinical Practice Guidelines: Management of Dengue Infections in Adults 3rd Edition (2015).

[2] Phan, L. et. al ALFA: Allele Frequency Aggregator. National Center for Biotechnology Information, U.S. National Library of Medicine, 10 Mar. 2020. Retrieved from: www.ncbi.nlm.nih.gov/snp/docs/gsr/alfa/

## P-30 Probiotics Alter Biofilm Formation of Periodontal Pathogen, *Porphyromonas gingivalis* virulence-associated genes

### Nurul Szawani Mohd Zubri, Rohazila Mohamad Hanafiah, Siti Aisyah Abdul Ghafar and Nor Zaihana Abdul-Rahman

#### Faculty of Dentistry, Universiti Sains Islam Malaysia, 55100, Kuala Lumpur, MALAYSIA

##### **Correspondence:** Nor Zaihana Abdul-Rahman (zaihana@usim.edu.my)

From The International Conference on Molecular Diagnostics & Biomarker Discovery 2022 (MDBD 2022)

Penang, Malaysia. 11 – 13 October 2022

**Background**

Probiotics, a group of beneficial live microorganisms are suggested as an adjunct therapy to control the colonization of periodontal pathogens. Periodontal pathogens, especially *Porphyromonas gingivalis*, can form biofilm with other pathogens in the oral cavity and exhibit virulent factors that are detrimental to human gum health [1]. This study was carried out to assess the anti-biofilm activity of probiotics isolated from local fermented food and to investigate the underlying molecular activity behind the anti-biofilm activity.

**Methodology**

Cell-free supernatant (CFS) was prepared by centrifugation and filter sterilisation of the overnight culture of *Lactobacillus plantarum* FT 5. The probiotics culture was isolated from local fermented food and is kept as a private collection [2]. Anti-biofilm assay was performed against *P. gingivalis* where the optical density of *P. gingivalis* biofilm stained with crystal violet solution in the treated and untreated groups was measured. Sterile MRS and 0.2% chlorhexidine acted as negative and positive control respectively. Then, RT-qPCR procedure involving *fimA, mfa1, kgp,* and *rgp* genes was carried out on the treated and untreated *P. gingivalis.* Additionally, the 16S rRNA gene was employed as the normalization gene.

**Results and Discussion**

The anti-biofilm assay showed a significant reduction of *P. gingivalis* biofilm with a 90.5% (p-value < 0.05) reduction at the highest concentration of *L. plantarum* FT 5 CFS. The RT-qPCR revealed that biofilm reduction might be related to the downregulation of two biofilm-related genes, *fimA* and *mfa1*. Interestingly, *P. gingivalis* treated with *L. plantarum* FT 5 CFS showed upregulation of *kgp* and *rgp* genes. The results implicate that the inhibition of *P. gingivalis* biofilm formation by *L. plantarum* FT 5 CFS are due to the downregulation of fimbriae-related genes, *fimA* and *mfa1* genes. Aside from being vital virulent genes, *kgp* and *rgp* also contribute to the maturation of fimbrial protein [3]. Thus, the upregulation of *kgp* and *rgp* in *P. gingivalis* is probably necessary to overcome the downregulation of fimbriae-related genes.

**Conclusion**

In conclusion, *L. plantarum* FT 5 isolated from fermented foods can reduce the biofilm formation by *P. gingivalis* and the underlying mechanism might be related to biofilm and virulent-related genes regulation.

**Acknowledgment:** The authors acknowledged the financial support from the Ministry of Higher Education Fundamental Research Grant Scheme for Research Acculturations of Early Career Researchers FRGS-RACER [grant number: USIM/FRGS-RACER/FPG-50419].

**References**

[1] M. A. Curtis, P. I. Diaz, and T. E. Van Dyke, “The role of the microbiota in periodontal disease,” *Periodontol. 2000*, vol. 83, no. 1, pp. 14–25, Jun. 2020, doi: 10.1111/prd.12296.

[2] N. Z. Abdul Rahman, R. M. Hanafiah, S. A. Abd Ghafar, N. Abdullah, and N. N. Azman, “Isolation and Antimicrobial Activity of Lactic Acid Bacteria against Streptococcus Mutans,” *J. Int. Dent. Med. Res.*, vol. 13, no. 2, pp. 417–421, Aug. 2020.

[3] J. Y. Lee, D. P. Miller, L. Wu, C. R. Casella, Y. Hasegawa, and R. J. Lamont, “Maturation of the Mfa1 Fimbriae in the Oral Pathogen Porphyromonas gingivalis,” *Front. Cell. Infect. Microbiol.*, vol. 8, p. 137, May 2018, doi: 10.3389/fcimb.2018.00137.

## P-31 Survival analysis of TNBC patients: A retrospective study from Hospital Universiti Sains Malaysia (HUSM)

### Nuramira Syazreen Suhaimi, Nur Amira Khairil Anwar, Elis Rosliza Mohd Adzmi and Noor Fatmawati Mokhtar

#### Institute for Research in Molecular Medicine (INFORMM), Health Campus, Universiti Sains Malaysia, Kelantan, Malaysia

##### **Correspondence:** Noor Fatmawati Mokhtar (fatmawati@usm.my)

From The International Conference on Molecular Diagnostics & Biomarker Discovery 2022 (MDBD 2022)

Penang, Malaysia. 11 – 13 October 2022

**Background**

Triple-negative breast cancer (TNBC), characterised by the absence of ER/PR expression with lack of HER2 overexpression, has drawn more attention both clinically and experimentally due to its aggressive biological characteristics and lack of effective treatment methods. There are very limited population-based breast cancer survival and prognostic factors studies for Malaysian population, particularly that focus on TNBC patients. Hence, the purpose of this study was to determine the survival of TNBC patients at HUSM and to identify potential prognostic factors for their overall survival (OS).

**Methodology**

This retrospective study was conducted using primary data of 50 women diagnosed with malignant breast cancer at HUSM between January 2017 and December 2019 (USM/JEPeM/18120775). The patients were divided into two groups: TNBC and non-TNBC. Overall survival curves were estimated using the Kaplan-Meier method and a log-rank test was used to compare the survival functions among the two groups. Simple Cox’s proportional hazard regression model was employed to identify the important prognostic factors of death.

**Results and Discussion**

The OS for TNBC was significantly lower than that for non- TNBC, at 45.0% vs 83.3% (*p* = 0.003). The mean survival time for TNBC was 38.7 months compared to 73.4 months for non- TNBC. Molecular subtype was a significant prognostic factor for malignant breast cancer patients whereby diagnosis with non- TNBC reduces the death hazard by a factor of 0.23 or 77.0% (95% CI 0.079–0.664; *p* = 0.007). Only staging status was statistically significant for OS of TNBC patients.

The OS for early-stage TNBC was significantly higher than that for advanced-stage TNBC, at 66.7% vs 27.3% (*p* = 0.021). The median survival time was 57.0 months (95% CI 50.5–63.5 months) for early-stage TNBC vs 24.0 months (95% CI 19.7–28.3 months) for advanced stage TNBC patients. Advanced stage TNBC patients had 5.3 times increased hazard to death compared to early-stage TNBC patients (95% CI 1.096–25.647; *p* = 0.038). Since no other variables showed statistical significance on OS of TNBC or non- TNBC patients, multivariate analysis was not conducted. The OS and important prognostic factors reported in this study are in accordance with other studies [1,2] that have recorded worse OS for TNBC patients diagnosed at advanced stage. Nonetheless, other factors including patient’s age, surgical treatment methods and family history of malignancy have been shown to be important prognostic factors for breast cancer [1,2], which was not the case for this study.

**Conclusion**

At HUSM, TNBC patients, particularly those diagnosed at advanced stage, have worse prognosis compared to non-TNBC patients. Future studies with larger sample size and comparative studies with other hospitals in Malaysia are necessary to enable the identification of more potential prognostic factors for TNBC and non-TNBC patients, with the aim to improve breast cancer management in this region.

**Acknowledgment:** This work was funded by Agilent Technologies (304/CIPPM/6150161/A146).

**References**

[1] Gonçalves H Jr, et al. Survival Study of Triple-Negative and Non-Triple- Negative Breast Cancer in a Brazilian Cohort. *Clin Med Insights Oncol*. 2018, 12

[2] Hsu JY, et al. Survival, treatment regimens and medical costs of women newly diagnosed with metastatic triple-negative breast cancer. *Sci Rep.* 2022, 12: 729

## P-32 The shark VNAR single-domain antibody as potential binder for SARS-CoV-2

### Hui Ting Lim^1^, Boon Hui Kok^1^, Chin Peng Lim^1,2^, Chiuan Yee Leow^2^ and Chiuan Herng Leow^1^

#### ^1^Institute for Research in Molecular Medicine (INFORMM), Universiti Sains Malaysia, 11800 Gelugor, Penang, Malaysia; ^2^School of Pharmaceutical Sciences, Universiti Sains Malaysia, 11800 Gelugor, Penang, Malaysia

##### **Correspondence:** Chiuan Herng Leow (herng.leow@usm.my)

From The International Conference on Molecular Diagnostics & Biomarker Discovery 2022 (MDBD 2022)

Penang, Malaysia. 11 – 13 October 2022

**Background**

Severe acute respiratory syndrome coronavirus 2 (SARS-CoV-2) as an emergent zoonotic virus since December 2019, has caused coronavirus disease 2019 (COVID-19) as the first coronavirus pandemic in history [1]. Neutralizing antibodies against SARS-CoV-2 is useful, however with the large size (~150 kilodaltons (kDa)), complex hetero-tetrameric structure and susceptibility to extreme ambient temperature for the conventional antibody may limit their performance in therapeutic and diagnostic applications [2]. Antibody surface display technology allows the exploration of antibody fragments from other organisms, including VNAR (variable domain of immunoglobulin new antigen receptor (IgNAR)) from shark with better thermostability [3]. The advantages of shark VNAR single-domain antibody are due to possessing smaller size (~12 kDa) and simpler structure, with its antigen-binding capability comparable to conventional antibody [2, 3]. The aim of this study is to evaluate the potential of shark VNAR as binders towards SARS-CoV-2 as target antigen.

**Methodology**

A proprietary phage-displayed semi-synthetic shark VNAR library was subjected to 3 rounds of biopanning. The commercial wild-type SARS-CoV-2 receptor binding domain (RBD) diluted in various concentration was used as target antigen in this study. The biopanning steps per round involved immobilizing antigen on immunotube, blocking immunotube with skimmed milk, incubating phages from antibody library in immunotube, washing off unbound phages while eluting bound phages, followed by the re-propagation of specific phage clones in *Escherichia coli* TG1. The phage particles harvested from culture supernatant were applied in the next round of biopanning. Polyclonal phage pool and monoclonal phage clones from each biopanning round were analysed by agarose gel electrophoresis, to detect the presence of VNAR insert.

**Results and Discussion**

Throughout rounds of biopanning, the amplification of phage clones was observed in Round 3, with 11-fold enrichment as compared to Round 1. Based on agarose gel electrophoresis result, the presence of VNAR insert increased from 60% (Round 1) to 100% (Round 3), indicated that SARS-CoV-2 RBD-targeted phage clones have been enriched throughout subsequent rounds of biopanning. The identification and functional characterization of potential clones are in process.

**Conclusion**

Shark VNAR has been proven as potential binders targeting towards SARS-CoV-2. Thus, further biological characterization is required to determine the specificity and sensitivity of the potential binders.

**Acknowledgement:** We would like to thank funding sponsored by Ministry of Higher Education for Fundamental Research Grant Scheme (FRGS) with project code: FRGS/1/2021/SKK06/USM/02/12 and FRGS/1/2022/ SKK0/USM/02/5 to complete this work.

**References**

[1] Liu YC, et al. COVID-19: The first documented coronavirus pandemic in history. *Biomed. J*. 2020, 43(4): 328-333.

[2] Cheong WS, et al. Diagnostic and therapeutic potential of shark variable new antigen receptor (VNAR) single domain antibody. *Int. J. Biol. Macromol*. 2020, 147: 369-375.

[3] Leow CH, et al. Single domain antibodies as new biomarker detectors. *Diagnostics*. 2017, 7(4): 52.

## P-33 Selecting antibacterial aptamers against an essential outer membrane protein in *Pseudomonas aeruginosa* via aptamer repurposing approach

### Rupany Selvam^1^, Yap Michelle Khai Khun^1^ and Tan Hock Siew^1, 2^

#### ^1^ School of Science, Monash University Malaysia, Bandar Sunway, Selangor, Malaysia; ^2^ Tropical Medicine and Biology Multidisciplinary Platform, Monash University Malaysia

##### **Correspondence:** Tan Hock Siew (tan.hocksiew@monash.edu)

From The International Conference on Molecular Diagnostics & Biomarker Discovery 2022 (MDBD 2022)

Penang, Malaysia. 11 – 13 October 2022

**Background**

Antimicrobial resistance is one of the major threats to global health, resulting in an increasing number of people suffering from severe illness or dying due to infections that were once easily curable with antibiotics. *Pseudomonas aeruginosa* represents one of the most concerning pathogens involved in antibiotic resistance where the World Health Organisation has classified this gram-negative bacterium as an ESKAPE organism, and it is also listed in the Priority 1: Critical list. Hence, in this project, we opted for a novel intervention by using aptamers to inhibit the growth of *Pseudomonas aeruginosa,* which can be cost-effective and less laborious. Besides, antimicrobial agents targeting the outer membrane protein (OMP) in Gram-negative bacteria can also be effective at killing or inhibiting bacterial growth as these proteins play an important role in bacteria survival as well as being exposed to the surface, which facilitates direct binding. Aptamer repurposing also reduces the cost and length of new drug development.

**Methodology**

Unmodified functional DNA aptamers from the Aptagen database were docked with the essential OMP using various docking tools such as HADDOCK 2.4 and HDOCK web server to determine the binding site and binding score of the various aptamers. Aptamers that bind to the active site with a good binding score were synthesised and folded into their 3D structures. *P. aeruginosa* cells were incubated with the aptamers overnight and the inhibitory effects on the bacterial growth curve were investigated.

**Results and Discussion**

Apt31 had the best HADDOCK score, and was found to bind near the active site of the OMP. Interestingly, this aptamer also exhibited significant antibacterial activity from the early stationary growth phase onwards. The addition of the aptamer after the late log phase also exhibited a similar effect. Besides, the antibacterial activity of apt31 was also dose-dependent. Hence, we can deduce that apt31 binds to the active site region and blocks the OMP activity. The major role of this OMP is to fold and insert β-barrel proteins into the outer membrane layer. As apt31 hinders the OMP activity, we postulate that the bacteria cells eventually die due to a lack of OMPs, which are essential for survival and virulence.

**Conclusion**

We show that the apt31, an aptamer that binds to an antitumor, is capable of binding to OMP in *P.aeruginosa.* The aptamer-OMP complex exhibits a significant antibacterial effect in a dose-dependent manner. Future experiments will include the expression of the recombinant OMP and determination of the dissociation constant (K_d_) of the aptamer-OMP complex.

**Acknowledgement:** This work was funded by the School of Science Strategic Funding Scheme 2021, Monash University Malaysia.

## P-35 Production of recombinant shark V_NAR_ single domain antibody specific against DENV Type 2 NS1

### Boon Hui Kok^1^, Hui Ting Lim^1^, Chin Peng Lim^1,2^, Chiuan Yee Leow^2^ and Chiuan Herng Leow^1^

#### ^1^Institute for Research in Molecular Medicine (INFORMM), Universiti Sains Malaysia, Penang, Malaysia; ^2^School of Pharmaceutical Sciences, Universiti Sains Malaysia, Penang, Malaysia

##### **Correspondence:** Chiuan Herng Leow (Chiuan Herng Leow (herng.leow@usm.my)

From The International Conference on Molecular Diagnostics & Biomarker Discovery 2022 (MDBD 2022)

Penang, Malaysia. 11 – 13 October 2022

**Background**

Dengue virus (DENV)) infection is one of the main global health issues leading to high morbidity and death rate. The accuracy of early diagnosis is the key for effective treatment. However, early diagnosis for DENV infection is a great challenge due to the similar onset symptoms of Dengue with other flaviviruses, concurrent infection and cross-reactivity among dengue serotypes and other flaviviruses [1]. Besides, the limitations of conventional monoclonal antibodies (mAbs) also affect the efficiency of diagnostic kits [2]. Alternatively, the variable new antigen receptor (V_NAR_) found in shark, has recently been identified as a promising diagnostic tool due to its excellent thermostability and capability to target on cryptic epitopes [3]. In this study, a potential clone that recognizing DENV Type 2 NS1 has been isolated and expressed. This recombinant protein was determined to be soluble and potentially developed as a novel reagent for DENV immunodiagnostic platform.

**Methodology**

A semi-synthetic library of shark V_NAR_ was used for antibody selection. Total of 3 rounds of biopanning was undertaken in respect to isolate potential binders which can specifically recognize DENV Type 2 NS1. The eluted phage from each round of biopanning were subject to polyclonal and monoclonal phage ELISA. After verified through DNA sequencing, the selected clone was expressed using bacteria expression system, followed by immobilized metal affinity chromatography (IMAC) purification process. The biological functions of recombinant V_NAR_ will then be characterized, including thermostability, specificity and sensitivity towards DENV Type 2 NS1.

**Results and Discussion**

For monoclonal phage ELISA, G3, Z3 and Z8 clones were identified to possess good affinity towards NS1 and lower cross reaction towards BSA. Thus, Z8 clone was selected to express as a 12 kDa protein with some optimization during protein expression. To verify target protein, the recombinant antibody reacted with anti-His6 antibody was performed using Western Blot. The functional assays of recombinant protein Z8 are currently underway.

**Conclusion**

Z8 clone isolated from biopanning has been identified as a potent binder. The recombinant anti-NS1 V_NAR_ Z8 antibody was successfully produced in a bacteria expression system. This new binder can be developed as new reagent for DENV immunodiagnostic platform.

**Acknowledgement:** We would like to thank funding sponsored by Ministry of Higher Education for Fundamental Research Grant Scheme (FRGS) with project code: FRGS/1/2021/SKK06/USM/02/12 and FRGS/1/2022/SKK0/USM/02/5 to complete this work.

**References**

[1] Wiwanitkit, V. Challenges in the diagnosis of dengue. Future Virol. 2020, 15(8):485-487.

[2] Cheong, W. S., et al. Diagnostic and therapeutic potential of shark variable new antigen receptor (VNAR) single domain antibody. Int J Biol Macromol. 2020, 147:369–375.

[3] Liu, J. L., et al. Isolation of anti-toxin single domain antibodies from a semi-synthetic spiny dogfish shark display library. BMC Biotechnol. 2007, 7:78.

## P-37 A fatal case of cerebral malaria complicated with *Gemella bergeri* bacteremia: Role of *Plasmodium* mitochondrial *cox3* gene and MALDI-TOF Mass Spectrometry for species identification

### Ummu Afeera Zainulabid^1, 2^, Nor Diyana Dian^*3*^, Jauhary Effendy Juma’at^4^, Petrick Periyasamy^2^, Najma Kori^2^, Cheah Saw Kian^5^, Asrul Abdul Wahab^4^ and Zulkarnain Md Idris^3^

#### ^1^Department of Internal Medicine, Faculty of Medicine, National University of Malaysia; ^2^Department of Internal Medicine, Kulliyyah of Medicine, International Islamic University Malaysia; ^3^Department of Parasitology and Medical Entomology, Faculty of Medicine, National University of Malaysia; ^4^Department of Microbiology and Immunology, Faculty of Medicine, National University of Malaysia; ^5^Department of Anesthesiology, Faculty of Medicine, National University of Malaysia

##### **Correspondence:** Ummu Afeera Zainulabid (ummuafeera@iium.edu.my)

From The International Conference on Molecular Diagnostics & Biomarker Discovery 2022 (MDBD 2022)

Penang, Malaysia. 11 – 13 October 2022

**Background**

Here, we described a fatal case of a 37-year-old returning traveller from Burkina Faso, West Africa, who presented with an acute fitting episode later diagnosed as cerebral malaria induced by *Plasmodium falciparum* based on microscopic examination and *Plasmodium* mitochondrial cytochrome c oxidase III (*cox3*) gene PCR target. Unfortunately, the patient passed away due to severe malaria with multiorgan failure complicated with *Gemella bergeri* bacteremia. *G. bergeri* was identified using MALDI-TOF mass spectrometry.

**Methodology**

Preliminary evaluation of blood film malaria parasite showed coinfection of *P. falciparum* and *Plasmodium malariae*. Further PCR-based detection analysis using the *Plasmodium* mitochondrial *cox3* gene was used to identify the malaria species. The patient’s blood culture revealed gram-positive cocci in clusters, and further identification using MALDI-TOF Mass Spectrometry was done. Informed consent to publish had been obtained.

**Results and Discussion**

The patient blood sample was positive for *P. falciparum* based on analysis using a PCR assay targeting the *cox3* gene. Further, analysis done at Makmal Kesihatan Awam Kebangsaan confirmed *P. falciparum* infection. His blood culture revealed gram- shown in our case, cerebral malaria induced by *P. falciparum* causes coma, long-term positive cocci in clusters, which was later identified as *G. bergeri* using MALDI-TOF mass spectrometry. Despite the initiation of intravenous artesunate and subsequent parasite count responding and showing parasite clearance, the complication is detrimental. As neurological consequences and death. The patient also had concomitant fatal *G. bergeri* bacteremia. Initial examination revealed that the patient had poor oral hygiene and a missing tooth which could be the source of *Gemella* infection, which is common in the oral cavity and a causative organism of septic shock.

**Conclusion**

This report is the first fatal case of cerebral malaria caused by *P. falciparum* coinfection with *G. bergeri* bacteremia. Therefore, it is critical to determine the exact aetiology for appropriate medical management. *Plasmodium* mitochondrial *cox3* gene and MALDI-TOF Mass Spectrometry are useful for species identification, as illustrated in this case.

## P-38 Association of *MTHFR* polymorphism in stroke risk and rehabilitation outcomes

### Eric Tzyy Jiann Chong^1^, Kok Yeow Phneh^2,#^, Syahiskandar Sybil Shah^3^, Yuen Kang Chia^4^, Dayang Maryama Awang Daud^5^, Elyana Jalil^3^, Chek Siang Kelvin Cheng^3^ and Ping-Chin Lee^1, 2^

#### ^1^Biotechnology Research Institute, Universiti Malaysia Sabah, Jalan UMS, 88400 Kota Kinabalu, Sabah; ^2^Faculty of Science and Natural Resources, Universiti Malaysia Sabah, Jalan UMS, 88400 Kota Kinabalu, Sabah; ^3^Department of Rehabilitation Medicine, Queen Elizabeth Hospital, Jalan Penampang, 88200 Kota Kinabalu, Sabah; ^4^Neurology Unit, Queen Elizabeth Hospital, Jalan Penampang, 88200 Kota Kinabalu, Sabah; ^5^Faculty of Medicine and Health Sciences, Universiti Malaysia Sabah, Jalan UMS, 88400 Kota Kinabalu, Sabah

##### **Correspondence:** Eric Tzyy Jiann Chong (eric_ctj@ums.edu.my; leepc@ums.edu.my)

From The International Conference on Molecular Diagnostics & Biomarker Discovery 2022 (MDBD 2022)

Penang, Malaysia. 11 – 13 October 2022

**Background**

There are 12.2 million new stroke cases annually and about 101 million people worldwide are living with stroke aftermath [1]. In Malaysia, stroke is the third leading cause of death. The rs1801133 single nucleotide polymorphism (SNP) in the methylenetetrahydrofolate reductase (*MTHFR*) gene has been linked to stroke pathogenesis in a recent meta-analysis [2]. To date, there is a lack of study addressing this SNP towards stroke risk and rehabilitation outcomes in the Malaysian population. Hence, this study aims to investigate the association of *MTHFR* rs1801133 SNP with the risk and rehabilitation outcomes in Malaysian stroke patients.

**Methodology**

The peripheral blood sample was collected from 251 age-matched individuals (113 stroke patients and 138 healthy controls without a stroke history) with written consent. The genomic DNA was extracted from these blood samples and the rs1801133 SNP was genotyped using a TaqMan® assay approach. The odds ratio and 95% confidence interval were calculated for risk association analysis. The stroke patients were subjected to a special-designed rehabilitation exercise and their pre- and post-exercise assessments including the Barthel index, Fugl-Meyer assessment-upper extremity, and Fugl-Meyer assessment-lower extremity were compared based on their genotypes.

**Results and Discussion**

This study showed that the presence of a T allele of the rs1801133 SNP had at least a 0.4-fold reduced risk of stroke. This suggests that the T allele of the rs1801133 SNP is protective against a stroke in the Malaysian population. Surprisingly, the finding of this study is in contrast with those reported in the meta-analyses [2,3]. Out of the 31 stroke patients who had completed their special-designed rehabilitation exercises, patients with a homozygous (C/C) genotype showed significant improvement in the post-rehabilitation assessments including the Barthel index, Fugl-Meyer assessment-upper extremity, and Fugl-Meyer assessment-lower extremity.

**Conclusion**

In conclusion, this study suggests that the *MTHFR* rs1801133 SNP is a potential biomarker for stroke risk and rehabilitation outcome predictions in Malaysian stroke patients. Data of this study could be useful for stroke management in the country.

**Acknowledgement:** This study was supported by the Ministry of Higher Education, Malaysia (TRGS0006-SG-2/2014) and Universiti Malaysia Sabah (SBK0180-SG-2014).

**References**

[1] World Stroke Organization. Global Stroke Fact Sheet 2022. Geneva, World Stroke Organization Press, 2022; p. 1-25.

[2] Zou XL, et al. A systematic review and meta-analysis expounding the relationship between methylene tetrahydrofolate reductase gene polymorphism and the risk of intracerebral hemorrhage among populations. Front. Genet. 2022, 13:829672.

[3] Luo Z, et al. Associations of the *MTHFR* rs1801133 polymorphism with coronary artery disease and lipid levels: a systematic review and updated meta-analysis. Lipids Health Dis. 2018, 17:191.

## P-39 RNU6B and miR-16 as stable normalisation control for relative RT-PCR of Urinary microRNA in patients with colorectal polyps

### Faizah Ahmad^1, 2^, Nurul Atiqah Muhamad Fauzi^1^, Mowaffaq Adam Ahmed Adam^1^, Muhammad Radzi Abu Hassan^2, 3^, Nil Amri Mohamed Kamil^4^ and Muhammad Amir Yunus^1^

#### ^1^Advanced Medical & Dental Institute (AMDI), Universiti Sains Malaysia, Penang, Malaysia; ^2^Clinical Research Centre, Hospital Sultanah Bahiyah, Kedah, Malaysia; ^3^Department of Medicine, Hospital Sultanah Bahiyah, Kedah, Malaysia; ^4^Department of Surgery, Hospital Sultanah Bahiyah, Kedah, Malaysia

##### **Correspondence:** Faizah Ahmad (faizah@crc.moh.gov.my)

From The International Conference on Molecular Diagnostics & Biomarker Discovery 2022 (MDBD 2022)

Penang, Malaysia. 11 – 13 October 2022

**Background**

Relative real-time polymerase chain reaction (RT-PCR) is an experimental technique widely used in quantifying the expression of a particular gene of interest. However, the reliability of gene expression data obtained through this technique is highly dependent on the stability of the housekeeping genes being used. Housekeeping genes act as a normaliser or endogenous reference in the relative quantification of a target gene and its expression may vary between different types and sources of samples. Studies on urinary microRNA as a potential disease biomarker are expanding, and the suitable housekeeping genes are yet to be confirmed. In this study, we assessed the stability of three commonly used references for microRNA i.e., hsa-miR-26b, hsa-miR-16, and RNU6B.

**Methodology**

This study was registered (NMRR-19-1188-47227), approved by the Medical Review and Ethics Committee (MREC) of the Ministry of Health Malaysia, and the Human Research Ethics Committee of Universiti Sains Malaysia (JEPeM, USM), and conducted following the Good Clinical Practice Guideline. The urine samples were collected from 24 patients (12 with colonic polyps and 12 without colonic polyps) scheduled for colonoscopy at Hospital Sultanah Bahiyah, Kedah, Malaysia. Colonic polyps’ diagnosis was confirmed by colonoscopy and histopathological report. MicroRNA was extracted from the urine supernatant based on the acid guanidinium thiocyanate-phenol-chloroform-potassium acetate-lithium chloride method described by Zununi Vahed et al. (2016) with a slight modification. Reverse transcription and primer design for RT-PCR were performed following protocol by Balcells et al. (2011) and Busk (2014), respectively. We performed RT-PCR with cycling conditions of initial denaturation (95°C, 5 min) followed by 45 cycles of denaturation (95°C, 15 sec) and annealing (60°C, 30 sec). The analysis was extended with melting curve analysis (60°C to 95°C) to assess RT-PCR efficiency. The stability of Ct values was evaluated using three algorithms i.e., BestKeeper, DeltaCq, and NormFinder. T-test was used to analyse whether a significant difference in mean Ct values of hsa-miR-26b, hsa-miR-16, and RNU6B existed between those two groups of patients.

**Results and Discussion**

There is no significant difference in mean Ct values of RNU6B, hsa-miR-26b, and hsa-miR-16 between the group of patients with colonic polyps and without colonic polyps (P-value = 0.204, 95% CI, -2.56, 0.58). This suggests that these three microRNAs are suitable candidates for normalisation control for relative RT-PCR. Analysis with all three algorithms showed that hsa-miR-16 alone had the best stability among the three microRNAs tested, while the stability improved when miR-16 and RNU6B were combined.

**Conclusion**

The findings in this study could be used as a guide to other research applying the relative RT-PCR technique in measuring gene expression of microRNA in human urine samples.

**References**

[1] Balcells, I., et al. Specific and sensitive quantitative RT-PCR of miRNAs with DNA primers. BMC Biotechnol. 2011; 11: 1-70.

[2] Busk, P. K. A tool for design of primers for microRNA-specific quantitative RT-qPCR. BMC Bioinformatics. 2014.; 15: 1-29.

[3] Zununi Vahed, S., et al. A microRNA isolation method from clinical samples. BioImpacts. 2016; 6 (1): 25-31.

## P-41 A novel mutation of the *NOTCH3* Gene in Malaysian patient with Cerebral Autosomal Dominant Arteriopathy with Subcortical Infarcts and Leukoencephalopathy (CADASIL)

### Ilia Nazihah Mohamad Ayob^1^, Amelia Azman^1^, Law Wan Chung^2^ and Yusnita Yakob^1^

#### ^1^Unit of Molecular Diagnostics, Specialised Diagnostics Centre, Institute for Medical Research, National Institutes of Health, Ministry of Health, Malaysia, ^2^Neuromedical Clinic, Sarawak General Hospital, Sarawak, Ministry of Health, Malaysia

##### **Correspondence:** Ilia Nazihah Mohamad Ayob (nazihah.ma@moh.gov.my)

From The International Conference on Molecular Diagnostics & Biomarker Discovery 2022 (MDBD 2022)

Penang, Malaysia. 11 – 13 October 2022

**Background**

Mutations in the *NOTCH3* gene are responsible for cerebral autosomal dominant arteriopathy with subcortical infarcts and leukoencephalopathy (CADASIL), adult-onset hereditary angiopathy leading to ischemic episodes, vascular dementia, and other neurologic deficits. Most *NOTCH3* mutations reported so far are rigidly stereotyped, involving the gain or loss of a cysteine residue in a specific *NOTCH3* epidermal growth factor (EGF)-like repeats on chromosome 19.p13.12 [1, 3].

**Methodology**

More than 100 blood samples of unrelated Malaysian patients with clinical/neuroimaging pictures suggestive of CADASIL were referred to our laboratory for molecular investigation. Genomic DNA was extracted from whole blood using Prepito DNA Blood250 Kit (PerkinElmer). Genetic diagnosis of CADASIL was performed by targeted PCR amplification of exons 3, 4, 5, 6, 11, 16 and 19, including exon-intron boundaries of the *NOTCH3* gene, followed by Sanger sequencing to detect causative mutations. Sequence analysis was performed using 3500 Genetic Analyzer (Applied Biosystems). To confirm the pathogenicity of new nucleotide variants, the identified sequence variants were assessed against several public databases and *in silico* prediction tools such as VarSome in accordance with ACMG guidelines.

**Results and Discussion**

Out of the 108 samples tested, 23 samples were found to harbour variants suggestive of CADASIL. Using PCR-directed sequence analysis, a total of seven mutations were identified. One was a novel missense mutation, C260S and six previously reported mutations, R110C, R141C, R182C, C194S, R544C and R1006C were listed in the Human Genome Mutation Database (HGMD). In this study, a 51-year-old female patient with novel missense mutation C260S is presented with sudden onset of left-sided weakness and MRI brain scan findings suggestive of CADASIL. VarSome predicted C260S as pathogenic, located in a mutational hotspot and two different missense changes determined to be pathogenic have been seen before. The nucleotide position was strongly conserved and the variant was not found in gnomAD, a population database for normal individuals. Furthermore, the pathogenicity score was 95% from multiple *in silico* predictors. This result is consistent with the CADASIL diagnosis for this patient.

**Conclusion**

Careful assessments of genealogical, clinical, and neuroimaging data in patients with lacunar stroke can aid in selecting patients with a high probability of finding mutations by genetic screening [2]. In our samples, 21% of Malaysian patients with ‘clinically suspected’ CADASIL received the definitive molecularly proven diagnosis. In contrast, if clinical suspicion is high, the remaining undiagnosed patients should be recommended for further testing of NOTCH3 exons, which were not included in this study. In addition, the discovery of new mutations expands the genetic spectrum of *NOTCH3*-related diseases, which will contribute to further study of this disease in the future. Correct diagnosis is not only important for the management of the patient but also crucial for genetic counselling and early diagnosis of at-risk family members.

**Acknowledgements:** The authors would like to thank the Director General of Health Malaysia for the approval to present these findings and the Director of the Institute for Medical Research (IMR) for all the support given towards this presentation. We would like to express our gratitude to the Head of SDC and all staff of Molecular Diagnostics Unit for their contribution. This work was funded by IMR.

**References**

[1] Youngho Kim, et al. Two novel mutations of the NOTCH3 gene in Korean patients with CADASIL, Mutation Research/Fundamental and Molecular Mechanisms of Mutagenesis, 2006, Volume 593, Issues 1–2, Pages 116-120.

[2] Abramycheva N, et al. New mutations in the Notch3 gene in patients with cerebral autosomal dominant arteriopathy with subcortical infarcts and leucoencephalopathy (CADASIL). *J Neurol Sci*. 2015;349(1-2):196-201.

[3] Kim Y, et al. Novel Characteristics of Race-Specific Genetic Functions in Korean CADASIL. *Medicina (Kaunas)*. 2019;55(9):521.

## P-42 Screening of *Lactobacillus* species in stingless bee honey

### Nur Baizura Sadom, Norra Ismail and Yangmurni Zamani

#### Food Science & Technology Research Centre, Malaysian Agriculture Research and Development Institute Selangor, Malaysia

**Correspondence:** Norra Ismail (baizura@mardi.gov.my)

From The International Conference on Molecular Diagnostics & Biomarker Discovery 2022 (MDBD 2022)

Penang, Malaysia. 11 – 13 October 2022

**Background**

Stingless bee honey is a natural substance produced by the stingless bee of genera *Meliponini*. Compared to honeybee honey, stingless bee honey has a significantly higher moisture content, water activity, and ash content, while the pH and total soluble solid content are slightly lower. Other components such as proteins, amino acids, enzymes, organic acids, mineral elements, and vitamins are also present in small amounts. Being rich in nutrients, honey provides a suitable environment for microflora growth. Microflora in honey includes yeasts, moulds, and bacteria with bacteria being the major microbes associated with stingless bee honey. Many studies have proven the presence of probiotics amongst the microflora, which includes *Lactobacillus, Lactococcus*, and *Bifidobacterium*. This finding has provided additional commercial value to stingless bee honey. Hence, this study aims to screen the presence of *Lactobacillus spp.,* one of the most accepted probiotics, in stingless bee honey of *Heterotrigona itama* collected from a plantation in Mantin, Negeri Sembilan.

**Methodology**

Samples were randomly obtained from July to November 2018 from the same plantation area. The samples were cultivated in lactobacilli de Man, Rogosa, Sharpe (MRS) broth before being subjected to isolation on MRS agar. This step was carried out 3 times to further isolate the potentially different species by differentiating them according to their colony morphology. 5 colonies isolated from each month were subjected to DNA extraction using QiaAmp DNA kits. The colonies were identified via polymerase chain reaction (PCR) using a set of primer pairs targeting the region of 16S rRNA; LF 5’-AGCAGTAGGGAATCTTCCA-3’ and LR 5’-ATTACACCGCTACACATG-3’, targeting amplicon sizing 430bp. PCR products were sent to 1st Base Laboratories for sequencing followed by sequence alignment using BLAST on the NCBI website.

**Results and Discussion**

Growth on MRS agar showed the growth of a few species that could be differentiated by their colony morphologies. Colonies colour ranged from whitish to brownish yellow, while other characteristics observed were sliminess, size, and shape; where there were round and slightly oval shape. Most samples subjected to PCR gave out clear single band sizing around 400bp during gel electrophoresis, while some other gave two to three, clear but faint bands ranging around the same size; 400 - 500bp. This could be explained by mixed species that co-inhibit the same honey pot. Sequencing and alignment of PCR products in the database showed that several *Lactobacillus* species repeatedly emerged on the list. This suggested that these species are dominant in the stingless bee colony of that plantation. Said species were *Lactobacillus kefiri* strain JK5, *Lactobacillus timberlakei* strain HV, *Lactobacillus malefermentans* JCM 12497, *Lactobacillus kefiranofaciens* strain IMAU98303 and *Lactobacillus salivarius* strain JCM1046.

**Conclusion**

This finding confirms that stingless bee honey provides a good variety of beneficial bacteria especially from *Lactobacillus* strains.

**Acknowledgement:** MInistry of Agriculture and Food Industry, MAFI (previously known as MOA) and Malaysian Agricultural Research and Development Institute (MARDI) for providing the funding and support.

## P-43 Interaction of UV-VIS optical properties towards enterovirus 71 RNA for detection of hand foot mouth disease using optical spectroscopy

### Ahmad Aiman Zuhaily Ahmad Munawar^1^, Irneza Ismail^1^, Fatin Hamimi Mustafa^2^, Wan Zakiah Wan Ismail^1^ and Juliza Jamaludin^1^

#### ^1^Faculty of Engineering and Built Environment, Universiti Sains Islam Malaysia, Nilai, Negeri Sembilan, Malaysia; Institute for Research in Molecular Medicine (INFORMM), Universiti Sains Malaysia Health Campus Kubang Kerian, Kelantan, Malaysia

##### **Correspondence:** Irneza Ismail (dr.irneza@usim.edu.my)

From The International Conference on Molecular Diagnostics & Biomarker Discovery 2022 (MDBD 2022)

Penang, Malaysia. 11 – 13 October 2022

**Background**

Hand-foot-mouth disease (HFMD) is a common infectious disease that occurs in children under 5 years old. The disease is transmitted through nasal fluids, saliva and contact act with surfaces contaminated with HFMD patients. HFMD is caused by viruses known as Enterovirus A71 (EV71). Generally, all viruses are biological things that contain specific genes of deoxyribonucleic acid (DNA) and ribonucleic acid (RNA). In this project, RNA extraction is used instead of DNA. RNA extraction is safer, stays inactivated at room temperature, and has resistance to ultraviolet sensitivity compared to DNA. Various types of light with different wavelengths are represented in the electromagnetic spectrum, and this research used Ultraviolet (UV) until Visible (VIS) spectra. This project aims to study the optical properties of EV71 RNA via UV-VIS spectroscopy for HFMD detection and to differentiate the UV-VIS light response between various samplings of EV71 RNA through spectra analysis. UV-VIS spectroscopy requires specialized methodologies to be performed. This project was implemented using absorption, transmission, and reflectance methodologies.

**Methodology**

In this study, EV71 RNA with different concentrations were used. Optical spectroscopy in the ultraviolet-visible (UV-VIS) range was used to observe the optical properties of all the RNAs, including absorbance, transmission and reflectance. The Ocean View software was used to capture the spectra of all honey. The spectra were collected more than 10 times for each concentration to improve the accuracy of the results. The average of the results was plotted, and analysis was performed for all spectra results.

**Results and Discussion**

As a result of this project, the value of absorbance and reflectance is directly proportional to the concentration whereas the value of transmittance is inversely proportional to the concentration. The results of this experiment were supported by theoretical and previous studies. The data of this research need continue to be discussed and improved from time in developing a diagnostic instrument that is responsive, mobile, lowcost, and accurate. A rapid, adaptive test, such as a “test kit” capable of identifying the EV71, will significantly improve the detection of HFMD. Thus, the annual number of HFMD cases can be controlled since the appropriate treatment can be administered due to the early detection of HFMD in the early stages.

**Conclusion**

Three optical properties, which are absorbance, transmission, and reflectance were studied for all EV71 RNA using optical spectroscopy in the UV-VIS ranges. An analysis was performed on the spectra results to compare the mean value of the peak spectra. Absorption and reflection have demonstrated a similar relationship between the sample concentration used and the absorbance or reflectance values. This means, when the sample concentration was high, the absorbance or reflectance value will also be increased. Contrarily, the association between sample concentration and transmittance were different. This signifies that the transmission value was low when the sample concentration was high.

**Acknowledgement:** The work was funded by the Internal Grant Scheme from the Universiti Sains Islam Malaysia (PPPI/USIM-RACER_0120/FKAB/051000/12820)

**References**

[1] Guerra, A. M.; Orille, E.; Waseem, M., Hand foot and mouth disease. In StatPearls [Internet], StatPearls Publishing: 2021.

[2] Zhou, Y.; Qiu, Q.; Luo, K.; Liao, Q.; Li, Y.; Cui, P.; Liang, L.; Cheng, Y.; Wang, L.; Wang, K., Molecular strategy for the direct detection and identification of human enteroviruses in clinical specimens associated with hand, foot and mouth disease. Plos one 2020, 15, (11), e0241614.

## P-44 Analysis of microsatellite instability in colorectal cancer patients from Hospital USM

### Nurfadhlina Musa^1^, Marahaini Musa^1^, Wan Faiziah Wan Abdul Rahman^2^, Tengku Ahmad Damitri Al-Astani^3^, Ahmad Aizat Abdul Aziz^1^, Mohamad Ros Sidek^1^, Rosline Hassan^4^, Zaidi Zakaria^5^ and Sarina Sulong^1^

#### ^1^Human Genome Centre, School of Medical Sciences, Universiti Sains Malaysia, Kubang Kerian, Kelantan, Malaysia; ^2^Department of Pathology; ^3^Department of Chemical Pathology; ^4^Department of Haematology; ^5^Department of Surgery, School of Medical Sciences, Universiti Sains Malaysia, Kubang Kerian, Kelantan, Malaysia

##### **Correspondence:** Sarina Sulong (ssarina@usm.my)

From The International Conference on Molecular Diagnostics & Biomarker Discovery 2022 (MDBD 2022)

Penang, Malaysia. 11 – 13 October 2022

**Background**

Microsatellites are short, repeating DNA segments vulnerable to mismatches during replication. Mismatch repair (MMR) protein complexes often correct DNA mismatch errors. DNA errors build-up due to the MMR system’s loss of function, causing microsatellite instability (MSI), a genetic hypermutability disorder. MSI is a molecular phenotype due to a defective DNA MMR system. Recent years have seen a rise in interest in DNA MMR deficiency as a critical biomarker to predict the therapeutic efficacy of immune checkpoint inhibitors for various malignant neoplasms. The study aims to investigate the profile of MSI status in colorectal cancer (CRC) patients from Hospital USM.

**Methodology**

The formalin-fixed, paraffin-embedded tissue blocks from archival colon cancer samples and adjacent normal tissue (n = 43) were randomly selected between 2018 and 2020. Genomic DNA extracted from tumors and corresponding normal tissues were used for MSI analysis using the Promega panel (5 mononucleotide markers: BAT-25, BAT-26, NR-21, NR-24, and MONO-27; and 2 pentanucleotide markers: Penta C and Penta D).

**Results and Discussion**

Of the 43 tissue samples studied, 21 samples came from men and 22 came from women. The median age of patients in the study group was 62 years. Four tumors (9.3%) demonstrated high MSI (MSI-H), one demonstrated low MSI (2.3%), and the remaining 38 (88.4%) tumors were microsatellite stable (MSS). Our finding was comparable with other studies which show that the incidence of MSI in tumors in CRC varies from 8.8% to 20.3%. Interestingly, patients in the MSI-H group were younger, with a median age of 39 years old compared with the study population with a median age of 62 years old. In constrast, a median age of 63 years was observed in the MSS group. According to a study, patients with CRC who had MSI-H were diagnosed earlier than those with MSS (1). Most selected tissue samples arise from the distal colon whereas smaller percentage were from proximal colon (16%). Notably, the incidence of proximal lesions in the MSI-H group (75%, 3/4) was higher than in the MSS group (9%, 4/39). The findings of researcher from several countries have demonstrated that MSI is more frequent in patients with tumors localization in the proximal of the colon (2), which is also in agreement with our findings. It has been reported that MSI-H tumors respond well to immunotherapy, PD-L1 (programed cell death ligand 1), with FDA approved to treat MSI-H/MMR patients.

**Conclusion**

This is an exploratory study employing a Promega panel to look at the MSI profile in our CRC patients. To associate clinical characteristics with MSI status, it is necessary to do further research with larger sample size. It would be beneficial to screen patients with MSI-H status which has a potential role in treatment management.

**Acknowledgement:** The work was funded by Newcastle University Medicine Malaysia (NUMED) (304/PPSP/6150173).

**References**

[1] Obert R, et al. The New England Journal of Medicine Tumor Microsatellite Instability And Clinical Outcome In Young Patients With Colorectal Cancer A Bstract Background Colorectal cancer can arise through. 2000;342:69.

[2] Bläker H, et al. The Association Between Mutations in BRAF and Colorectal Cancer–Specific Survival Depends on Microsatellite Status and Tumor Stage. Clin Gastroenterol Hepatol [Internet]. 2019;17(3):455-462.e6. Available from: https://doi.org/10.1016/j.cgh.2018.04.015

## P-46 Antimicrobial activity of astaxanthin extracts of wild type and mutant *Xanthophyllomyces dendrorhous*

### N. Sathiabalan, S.Y. Khaw and A.L. Chew

#### Institute for Research in Molecular Medicine (INFORMM), Universiti Sains Malaysia, Penang, Malaysia

##### **Correspondence:** A.L. Chew (chew@usm.my, ai_lan@hotmail.com)

From The International Conference on Molecular Diagnostics & Biomarker Discovery 2022 (MDBD 2022)

Penang, Malaysia. 11 – 13 October 2022

**Background**

There is worldwide concern about the increase in antimicrobial resistance which affects both developed and developing countries. It is a public health issue with immense societal and economic implications [1]. Antibiotic-resistant pathogens pose an enormous threat to the treatment of a wide range of serious infections. A periodic replacement of the existing antibiotic is necessary to prevent this exponential emergence. *X. dendrorhous* extracts were signified on the benefits of astaxanthin in antioxidant activity, but less emphasized for its antimicrobial activity. This study aims to assess the antimicrobial ability of astaxanthin extract of wild type and mutant *X. dendrorhous* obtained in our research.

**Methodology**

Antimicrobial activity of astaxanthin extracts was evaluated through the zone of inhibition using paper disc assay against seven microorganisms of industrial importance Gram-negative bacteria: *Shigella boydii* and *Escherichia coli*, Gram positive bacteria: *Bacillus subtilis* and *Staphylococcus aureus*, yeasts: *Saccharomyces cerevisiae* and *Candida albicans*, and fungus: *Aspergillus niger*. The wild type and mutant astaxanthin extracts were prepared at a concentration of 30ug/mL and dotted on autoclaved paper discs. The experiment also included a positive control of 30ug/mL of chloramphenicol for antibacterial drugs and Miconazole as a standard antifungal drug. All the plates containing bacteria were incubated at 37°C for 24 h and that of yeast and fungi at 28°C for 48 h.

**Results and Discussion**

The standard antibiotic chloramphenicol produced inhibition zones of 12, 14, 9 and 8mm against *B. subtilis*, *S. aureus*, *S. boydii* and *E. coli* respectively. Meanwhile positive control Miconazole produced inhibition zones of 28, 25 and 23 mm against *S. cerevisiae*, *C. albicans* and *A. niger* respectively. The antimicrobial activity of both wild type and mutant extracts showed significant growth inhibition against pathogenic microbial strains. The mutant astaxanthin extract showed a slightly better inhibition effect on all the pathogens than the wild type extract, but the difference was not significant. The extracts exhibited an excellent inhibitory effect against Gram-positive *B. subtilis* and *S. aureus*, nearly as good as positive control. They exerted a milder antimicrobial capability against Gram negative *S. boydii* and *E. coli*. Compared to the positive control, the extracts showed moderate inhibition in *S. cerevisiae, C. albicans* and *A. niger* for yeast and fungus. Limited antimicrobial reports on astaxanthin are mainly extracted from sources like microalga *H. pluvialis* and crustaceans. Some contradictions can be found in the literature due to the different strains used in the determination of antimicrobial activity, extraction methods, extraction solvents and extract concentration used in the assays [2].

**Conclusion**

Based on controls, both the wild type and mutant astaxanthin extracts showed impressive inhibition on Gram positive and Gram negative bacteria compared to yeast and fungus. The twofold use of astaxanthin extracts as antioxidants and antimicrobials has good prospects in response to the consumer demand for more sustainable and natural ingredients and products.

**Acknowledgement:** The authors would like to express their gratitude to Universiti Sains Malaysia for Research University Grant (RUI Grant no: 1001/CIPPM/8012377)

**References**

[1] Dhingra S, et al. Microbial Resistance Movements: An Overview of Global Public Health Threats Posed by Antimicrobial Resistance, and How Best to Counter. Front. Public Health. 2020, 8: Article 535668.

[2] Irna C, et al. Antioxidant and antimicrobial activities of astaxanthin from Penaeus monodon in comparison between chemical extraction and High Pressure Processing (HPP). Int. Food Res. J. 2017. 24(Suppl): S508-S513.

## P-47 The potential of *Xanthophyllomyces dendrorhous* wild type and mutant strains in producing UV absorbing compounds

### N. Sathiabalan and A.L. Chew

#### Institute for Research in Molecular Medicine (INFORMM), Universiti Sains Malaysia, Penang, Malaysia

##### **Correspondence:** A.L. Chew (chew@usm.my; ai_lan@hotmail.com)

From The International Conference on Molecular Diagnostics & Biomarker Discovery 2022 (MDBD 2022)

Penang, Malaysia. 11 – 13 October 2022

**Background**

Solar ultraviolet radiation is the culprit for molecular and genetic changes that occur to the skin. It generates reactive oxygen species and free radicals that cause skin oxidative damage leading to skin cancers, photosensitivity, and photo-aging problems. These contribute to synthetic sunscreens formulated with UVR filters that mostly show side effects on human health and ecology. Hence, research investigations were initiated upon natural resources that can produce UV-absorbing compounds known as mycosporine-like amino acids and mycosporine-glutaminol-glucoside. These molecules are commonly used by organisms growing at high altitudes to protect themselves from extreme UV exposure [1]. In this study, a basidiomycetous yeast, *Xanthophllomyces dendrorhous* wild type and its mutant strains were analyzed for their ability to produce MAA/MGGs**.**

**Methodology**

*X. dendrorhous* wild type and mutant strains were revived and maintained on yeast malt agar at 4°C. Synthesis of mycosporines was induced by transferring young cultures (24hr) to YM agar plates and incubated for 4 days at 22°C in an incubator (LabCompanion SIF-6000R) with a 12:12 light: dark photoperiod. For light conditions, culture plates were left exposed to PAR+UVR while for dark conditions, plates were covered with aluminium foil. After exposure, the colonies were harvested and transferred to distilled water, centrifuged, and conserved at -20° until mycosporines extraction. Samples were extracted with 10ml of DMSO solution (2%) for 24hrs at 4°C. After 24hrs, glass beads were added to samples and vigorously agitated in a vortex mixer. Later, they were incubated for 24hrs at 45°C in a water bath and centrifuged using a microcentrifuge. The resulting supernatant was immediately measured spectrophotometrically (UV-visible) at 325 nm for mycosporines quantification. Experiments were performed in triplicate.

**Results and Discussion**

With the aid of a UV spectrum, the mycosporine production rate was found to be the highest at 325 nm for all four strains leading to a fixed wavelength for further measurements. Yeast colonies exposed to PAR+ UVR showed higher absorbance rates concluding more mycosporines production than those grown in a dark environment. It showed that strains frequently exposed to high UV radiation have an increased tendency of mycosporine production than those with lower intensity of UV exposure. By comparing the wild type and 3 mutant strains, the results indicated that the red, white and yellow mutant colonies initially showed almost equal mycosporines production rate as the wild strain. The production rate will be further improved by introducing different inducers, growth media and culture conditions.

**Conclusion**

All the four strains tested showed the detection of MAA/MGGs in the DMSO extracts. It is concluded that after photostimulation, both *X. dendrorhous* wild type and mutant strains have the ability to synthesize UV-absorbing compounds and further investigation should be done to discover its potential as a natural molecular sunscreen.

**Acknowledgement:** The authors would like to express their gratitude to Universiti Sains Malaysia for Research University Grant (RUI Grant no: 1001/CIPPM/8012377)

**Reference**

[1] S. Davinelli, M. E. Nielsen, and G. Scapagnini, “Astaxanthin in Skin Health, Repair, and Disease: A Comprehensive Review.,” *Nutrients*, vol. 10, no. 4, Apr. 2018, doi: 10.3390/nu10040522.

## P-50 Comparative evaluation of three methods for protein extraction efficiency from formalin-fixed paraffin-embedded tissues for proteomic analysis

### Wan Muhammad Shafiq^1^, Gaayathri Kumarasamy^1^, Mohd Nazri Ismail^1, 2^ and Gurjeet Kaur^1^

#### ^1^Institute for Research in Molecular Medicine (INFORMM), Universiti Sains Malaysia, 11800 Minden, Pulau Pinang, Malaysia; ^2^ Analytical Biochemistry Research Centre (ABrC), Universiti Sains Malaysia, 11900 Bayan Lepas, Pulau Pinang, Malaysia

##### **Correspondence:** Gurjeet Kaur (gurjeet@usm.my)

From The International Conference on Molecular Diagnostics & Biomarker Discovery 2022 (MDBD 2022)

Penang, Malaysia. 11 – 13 October 2022

**Background**

Formalin fixation and paraffin embedding of tissues (FFPE) have become the most available biopsies in the hospital setting due to their ability to be stored for long periods and may be kept at room temperature without concern of deterioration or decay. However, this fixation process causes a protein alteration known as “cross-links.” Consequently, it made it challenging to extract proteins and characterize individual proteins. The study of proteomics is growing quickly because it has the potential to enhance disease diagnosis, risk assessment, prognosis, and therapy targeting. Consequently, as a researcher, it is important to investigate strong and efficient protein identification approaches and improve all aspects of protein analysis, such as the protein extraction method from FFPE tissue and protein digestion for mass spectrometry.

**Methodology**

For this research, FFPE human tonsillitis blocks were chosen for this research to compare the efficiency of three different protein extraction methods, including sonication, RapiGest, and Sodium Deoxycholate, from Formalin Fixed Paraffin Embedded (FFPE) tissue for proteomic analysis. These sections were cut into 4 μm thin slices of tissue to facilitate the deparaffinization process. Three sets of unstained FFPE tonsil tissue sections were excised into 1.0 cm2 for the triplicate results. For the quantification analysis, Nanodrop 2000 was used to measure the protein concentration from the extracted protein. While the determination of the protein quality, SDS-PAGE analysis was done with three different protein extraction methods. The proteins of interest are digested with an enzyme, trypsin, and the resultant peptides are examined using mass spectrometry. Protein identification will be accomplished using LC-MS/MS analysis of protein extracted from three sets of each extraction technique obtained from FFPE tonsillitis samples.

**Results and Discussion**

The protein concentration of the samples was determined using the nanodrop 2000c. From the sonication method, the protein concentration was extremely low and inconsistent. The sodium deoxycholate method produced a greater protein concentration than RapiGest. Next, the protein quality was evaluated using SDS-PAGE following the application of the three protein extraction methods for sample fragmentation. The intensity of the band for RapiGest and Sodium Deoxycholate can be observed at 1 cm^2^. However, a very faint band was observed for the sonication method. This shows that the highest abundance of protein loaded into the gel is from the RapiGest and Sodium Deoxycholate, while the lowest is from the sonication method. All the proteins were then analysed via LC/MS and identified using Peak 7.5 software. A total of 668 proteins were successfully identified using the sodium deoxycholate method. In addition, a total of 387 protein samples were obtained using the sonication method. However, for the RapiGest method, the number of proteins acquired from the samples was only 89, which was the lowest. Based on the results obtained from the software, 14 proteins were found to overlap in all three protein extraction methods.

**Conclusion**

In conclusion, the most efficient protein extraction method is sodium deoxycholate because the protein concentration is the highest. Adding to that, the number of proteins identified using this method is also the highest.

## P-51 Optimization of expression and purification of soluble *E. bieneusi* B7XHF2 recombinant protein in *E.coli*

### Muhammad Danial Hakim Muhammad Hanif^1^, Putri Sabrina Mohamed Yusoff^2^, Norsyahida Arifin^1^ and Emelia Osman^2^

#### ^1^Institute for Research in Molecular Medicine (INFORMM), Universiti Sains Malaysia, 11800 Penang, Malaysia; ^2^Department of Parasitology and Medical Entomology, Faculty of Medicine, Universiti Kebangsaan Malaysia, 56000 Cheras, Kuala Lumpur, Malaysia

##### **Correspondence:** Emelia Osman (emelia.osman@ukm.edu.my)

From The International Conference on Molecular Diagnostics & Biomarker Discovery 2022 (MDBD 2022)

Penang, Malaysia. 11 – 13 October 2022

**Background**

Worldwide reports of human infections with *Microsporidia* mainly include immunocompromised patients [1]. One of the most prevalent microsporidian species to infect HIV/AIDS patients is *Enterocytozoon bieneusi* [2]. Although stool testing is the most reliable method to diagnose microsporidiosis, the infection is underdiagnosed in patients with disseminated microsporidiosis due to the absence of microsporidia spores in their stool samples. Our previous study has identified two circulating antigens of diagnostic value from the serum of immunocompromised patients. In this study, we aimed to express and purify *E.bieneusi* recombinant protein B7XHF2 in *Escherichia coli* as a potential diagnostic biomarker for the detection of disseminated microsporidiosis.

**Methodology**

The B7XHF2 gene was cloned in the pET-32a(+) vector, and the recombinant plasmid was transformed into C41(DE3) *E.coli* host cell. Small scale expression in 200ml Terrific broth (TB) was performed to optimize the parameters of protein expression namely IPTG concentration and post-induction incubation period. Subsequently, protein expression was carried out in 2L TB medium, at a temperature of 37°C, 200 rpm before the temperature was lowered to 28°C for 4 hours post IPTG induction. The harvested cells were pelleted and lysed by sonication in a lysis buffer containing protease inhibitors and lysozyme. The filtered protein lysate was then subjected to affinity purification using Ni-NTA resin at a ratio of 1:10 (resin:lysate). Contaminating proteins were removed by gradient washing using washing buffer containing 20mM, 30mM and 40mM imidazole concentration, and finally the targeted protein was eluted by adding elution buffer with 250mM imidazole. Eluted fractions were pooled and concentrated to 300ul and the protein concentration was measured by the RCDC method (Biorad^TM^). Anti-histidine western blot was carried out to verify the presence of the targeted protein.

**Results and Discussion**

In this study, the rB7XHF2 protein was highly soluble when induced with 0.5mM IPTG compared to 1.0mM IPTG. There was no significant difference in terms of protein expression when incubation was performed at 2,3,4 and 16 hours post-induction, therefore 4 hours was chosen as the best incubation period. The appearance of a 41-kDa protein on anti-histidine western blot confirmed the expression of rB7XHF2 protein, which was close to the theoretical size of 41.7 -kDa.

**Conclusion**

This preliminary study has successfully expressed the B7XHF2 recombinant protein in *E.coli* C41(DE3) host cells. Further work is needed to improve the yield as well as purity of the target protein for diagnostic applications.

**References**

[1] Zainudin NS et. al., 2016. Diagnosis of disseminated microsporidiosis: detection of circulating Enterocytozoon bieneusi DNA in blood of HIV/AIDS patients. Trop Biomed 33: 761–770

[2] Karimi K et. al., Molecular epidemiology of Enterocytozoon bieneusi and Encephalitozoon sp., among immunocompromised and immunocompetent subjects in Iran. Microb Pathog. 2020 Apr;141:103988. doi: 10.1016/j.micpath.2020.103988. Epub 2020 Jan 21. PMID: 31972268.

## P-54 *In silico* selection of aptamers to cobra venom cytotoxin

### Hiu Jia Jin, Tan Hock Siew, Yap Michelle Khai Khun

#### School of Science, Monash University Malaysia, Bandar Sunway, Malaysia

##### **Correspondence:** Yap Michelle Khai Khun (yap.michelle@monash.edu)

From The International Conference on Molecular Diagnostics & Biomarker Discovery 2022 (MDBD 2022)

Penang, Malaysia. 11 – 13 October 2022

**Background**

Snakebite envenomation has been listed as one of the neglected tropical diseases (NTDs) by the World Health Organization (WHO) since 2017 [1]. Cytotoxin (CTX), one of the most abundant venom components, was deemed responsible for the envenomation’s local dermonecrosis. Several studies have indicated that the CTX induces cell death through different pathways, yet the molecular mechanism is inconclusive [2]. There is also limited treatment against dermonecrosis. Therefore, there is a need to search for biotherapeutics against CTX-induced dermonecrosis. In this study, we exploit the potential of tRNA-based aptamers as alternate toxin-neutralizing molecules using in silico approach.

**Methodology**

GtRNAdb database was used to identify the CTX-binding tRNA-based aptamers for the possible interacting regions with CTX [3]. The sequences of the aptamers were subjected to phylogenetic tree analysis to categorize the tRNAs into different clusters using the Clustal Omega. The tertiary structures of the consensus aptamer sequences were modeled using the RNA Composer. Later, the molecular docking of these aptamers and CTX was performed using HADDOCK web server. In addition, RING analysis was performed on each aptamer-CTX complex to determine the interacting amino acids present on the CTX and the type of interactions.

**Results and Discussion**

Altogether, our results suggested six clusters of consensus aptamer sequences from the phylogenetic tree analysis. Among the six aptamer models generated from their respective consensus sequences, three aptamers demonstrated significant binding to CTX. Additionally, the RING analysis revealed that there were four common interacting regions between the six CTX-aptamer complexes. These four interacting regions were consistent with the CTX’s epitopes in our unpublished work. Moreover, the high binding affinity between the aptamer-CTX complexes also suggested that they were potential aptamers to antagonize CTX’s action.

**Conclusion**

The current work provides insights into *in silico* approach to design aptamers that antagonize CTX-induced dermonecrosis. Three tRNA-based aptamers have been identified as potential aptamers, which showed strong interaction with CTX’s epitopes.

**Acknowledgement:** This work was funded by the Royal Society of Tropical Medicine and Hygiene Small Grants Programme 2021.

**References**

[1] Chippaux JP. Snakebite envenomation turns again into a neglected tropical disease! J. Venom. Anim. Toxins. Incl. Trop. Dis. 2017, 23:38.

[2] Hiu JJ, et al. The myth of cobra venom cytotoxin: More than just direct cytolytic actions. Toxicon X. 2022, 14: 100123.

[3] Chan PP ey al. GtRNAdb 2.0: an expanded database of transfer RNA genes identified in complete and draft genomes. Nucl. Acid. Res. 2016, 44: 184-189.

## P-55 IgG2a subclass response of mice towards vaccination of *Strongyloides stercoralis* antigens

### Matthew Tze Jian Wong, Rahmah Noordin and Gee Jun Tye

#### Institute for Research in Molecular Medicine (INFORMM), Universiti Sains Malaysia, Penang, Malaysia

##### **Correspondence:** Gee Jun Tye (geejun@usm.my)

From The International Conference on Molecular Diagnostics & Biomarker Discovery 2022 (MDBD 2022)

Penang, Malaysia. 11 – 13 October 2022

**Background**

It has been tried and proven that vaccination is still the best strategy to combat infectious diseases. However, till date, there are still no vaccines against strongyloidiasis, caused by *Strongyloides stercoralis*. Approximately 100 million people are estimated to be infected worldwide, most commonly in tropical and subtropical regions. There are very few studies on vaccine candidates for strongyloidiasis. To date, there is only one study that uses recombinant protein from *S. stercoralis*, Ss-IR [1]. Furthermore, another study performed immunoscreenings with anti-human IgE antibody of an *S. stercoralis* complementary DNA (cDNA) library, with the results that the cDNA clone A-133 having the highest diagnostic potential [2]. Seeing the protein Ss-IR and A-133 having high potential as vaccine candidate against strongyloidiasis, this study aims to investigate the effects of vaccination with rSs-IR and rA-133 on levels of serum IgG2a antibody.

**Methodology**

rSs-IR and rA-133 conjugated with histidine tags will be expressed using *Escherichia coli* C41 (DE3) and purified via Ni-NTA affinity column chromatography. The purified antigens will be used for immunization of female Balb/c mice that will be divided into four test groups, namely unvaccinated control (n=6), adjuvant-only control (n=8), rA-133 treatment (n=13) and rSs-IR treatment (n=12). Priming dose will be administered via intraperitoneal route adjuvanted with Complete Freund’s Adjuvant (CFA), whereas subsequent booster doses will be administered via subcutaneous route adjuvanted with Incomplete Freund’s Adjuvant (IFA). Serum samples will be obtained via blood bled via tail snip. After that, ELISA will be performed on serum samples to monitor the level of subclass IgG2a antibodies.

**Results and Discussion**

The IgG2a levels in mice sera increased with each administration of rSs-IR and rA-133 respectively. Furthermore, rA-133 elicited a greater increase in IgG2a serum levels compared to rSs-IR. IgG antibodies are primarily involved in anti-pathogenic adaptive immune response and long-term protection, and IgG isotype antibody production dictates the robustness of immune response following antigen immunization. The finding of a higher IgG2a production stimulated by rA-133 is a favorable outcome for the antigen as vaccine candidate, due to the important roles IgG2a play in other parasitic diseases. For instance, it has been shown that IgG2a can activate macrophages that traps tissue-migrating helminths larvae thus immobilizing it [3].

**Conclusion**

In conclusion, this profile of high IgG2a expression shown by rA133 in this study is an important finding since more studies into parasitic carbohydrate-based vaccines are being conducted in order to generate similar antibody profiles. Furthermore, the result of this study provides a rough clue in figuring out the immune responses towards *S. stercoralis*.

**Acknowledgement:** This study was funded by the Ministry of Higher Education Malaysia, Fundamental Research Grant Scheme 203/CIPPM/6711636.

**References**

[1] Abraham D, Hess JA, Mejia R, Nolan TJ, Lok JB, Lustigman S, et al. Immunization with the recombinant antigen Ss-IR induces protective immunity to infection with Strongyloides stercoralis in mice. Vaccine 2011;29:8134–40. https://doi.org/10.1016/j.vaccine.2011.08.030.

[2] Ahmad H, Arifin N, Nolan TJ, Lok JB, Anuar NS, Noordin R. Strongyloides-specific IgE Phage cDNA Clones and Development of a Novel ELISA for Strongyloidiasis. Diagnostics 2021;11:1–16. https://doi.org/10.3390/diagnostics11060985.

[3] Bieren JE, Volpe B, Kulagin M, Sutherland DB, Guiet R, Seitz A, et al. Antibody-Mediated Trapping of Helminth Larvae Requires CD11b and Fcγ Receptor I. The Journal of Immunology 2015;194:1154–63. 10.4049/JIMMUNOL.1401645.

